# Phenanthrene-Extended Phenazine Dication: An Electrochromic Conformational Switch Presenting Dual Reactivity

**DOI:** 10.1021/jacs.2c00493

**Published:** 2022-04-12

**Authors:** Jacopo Dosso, Beatrice Bartolomei, Nicola Demitri, Fernando P. Cossío, Maurizio Prato

**Affiliations:** † Department of Chemical and Pharmaceutical Sciences, CENMAT, Centre of Excellence for Nanostructured Materials, INSTM UdR Trieste, University of Trieste, via Licio Giorgieri 1, 34127 Trieste, Italy; ‡ Centre for Cooperative Research in Biomaterials (CIC BiomaGUNE), Basque Research and Technology Alliance (BRTA), Paseo de Miramón 194, 20014 Donostia San Sebastián, Spain; § Basque Fdn Sci, Ikerbasque, 48013 Bilbao, Spain; ∥ Elettra—Sincrotrone, Trieste S.S., 14 Km 163.5, Area Science Park, 34149 Basovizza, Trieste, Italy; ⊥ Departamento de Química Orgánica I, Instituto de Innovaciónen Química Avanzada (ORFEO-CINQA), University of the Basque Country (UPV/EHU), Paseo Manuel Lardizabal 3, 20018 Donostia/San Sebastián, Spain; # Donostia International Physics Center (DIPC), Paseo Manuel Lardizabal 4, 20018 Donostia/San Sebastián, Spain

## Abstract

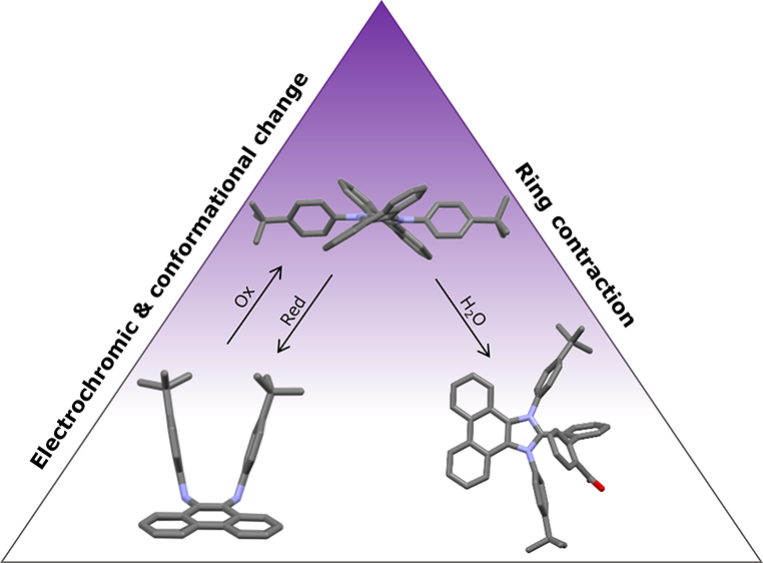

The synthesis and isolation of one of the few examples of a π-extended diamagnetic phenazine dication have been achieved by oxidizing a phenanthrene-based dihydrophenazine precursor. The resulting dication was isolated and fully characterized, highlighting an aromatic distorted structure, generated by the conformational change upon the oxidation of the dihydrophenazine precursor, which is also correlated with a marked electrochromic change in the UV–vis spectrum. The aromaticity of the dication has also been investigated theoretically, proving that the species is aromatic based on all major criteria (structural, magnetic, and energetic). Moreover, the material presents an intriguing dual reactivity, resulting in ring contraction to a π-extended triarylimidazolinium and reduction to the dihydrophenazine precursor, depending on the nature of the nucleophile involved. This result helps shed light on the yet largely unexplored reactivity and properties of extended dicationic polycyclic aromatic hydrocarbons (PAHs). In particular, the fact that the molecule can undergo a reversible change in conformation upon oxidation and reduction opens potential applications for this class of derivatives as molecular switches and actuators.

## Introduction

**Figure 1:**
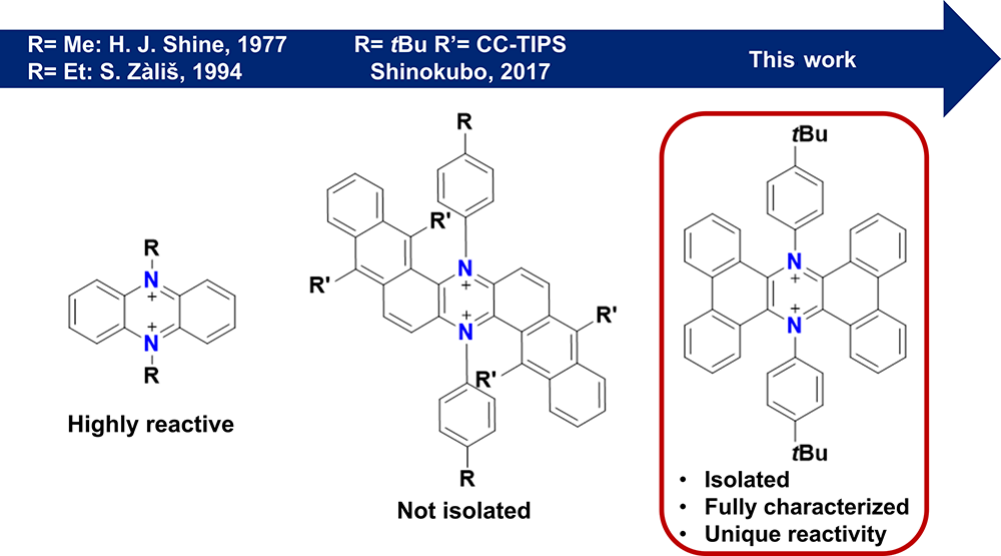
Previous examples of diamagnetic phenazinium dications and the target molecule of this work.

Almost 20 years after the first isolation of graphene,^1^ the bottom-up chemical synthesis of nanographenes and extended polycyclic aromatic hydrocarbons (PAHs) is widely recognized as one of the most thriving fields in chemistry.^2,3^ Along with the tailoring of the size and edges,^4−6^ the introduction of dopants in the aromatic scaffolds has become one of the leading strategies to control the properties of these materials.^2,7^ From this point of view, while many examples including a plethora of elements are present in the literature, the introduction of positively charged heteroatoms is much less explored, mainly due to synthetic difficulties and reactivity. While being challenging, the preparation of positively charged doped PAHs is potentially highly rewarding. In fact, this approach could result not only in semiconducting materials presenting charge transfer states but also in the introduction of highly polar bonds that are useful for self-assembly and sensing.^8−12^ Moreover, the possibility of introducing the heteroatoms in a neutral state and oxidizing them in a later step can result in materials with a behavior depending on the oxidation state and in molecular switching capabilities due to hybridization change.^13,14^ While various examples of PAHs presenting a single charged atom in their structure have been reported in different contributions,^2,7,8,15,16^ doubly charged ones are less explored, with notable examples presenting O, N, S, or combinations of these heteroatoms, using both surface and solution-based chemistry.^17−26^ From this point of view, parent and π-extended dihydrophenazines represent valuable precursors toward the generation of 2^+^ N-doped PAHs. These derivatives are known to be easily oxidized and have been synthesized by various groups,^27−31^ leading to their use as emitters, redox-active materials, or organocatalysts.^32−37^ Interestingly, while the radical cations of such derivatives have been extensively explored,^30,34,38,39^ the aromatic dicationic diamagnetic states have been widely neglected and studied almost exclusively in solution,^39,40^ with some notable exceptions successfully isolating N-alkyl derivatives of the parent phenazinium dication, which, however, were reported as highly reactive (Figure 1).^41,42^ More recently, an elegant work from the group of Shinokubo reported the synthesis of an anthracene-extended dihydrophenazine (Figure 1), which was oxidized in solution to the radical and dicationic states, resulting in a marked electrochromism.^40^ Moreover, from nuclear overhauser effect spectroscopy (NOESY) studies, a conformational change upon oxidation was evidenced in the molecule, possibly paving the way for future molecular actuators.^40^ Building on this, obtaining stable phenazinium dication derivatives is highly desirable because it would allow the study of these material properties and reactivity more in depth, possibly leading to selective chemical oxidation/reduction reactions enabling their future application as molecular switches or electrochromic systems. To obtain phenazinium dications with increased stability, the synthesis of a phenanthrene-based π-extended dihydrophenazine presenting armchair edges and a fully benzenoid Clar resonance structure was carried out. This work resulted in the isolation and complete characterization of an uncommon π-extended phenazinum dication, with a focus on its reactivity.

## Results and Discussion

**Scheme 1:**
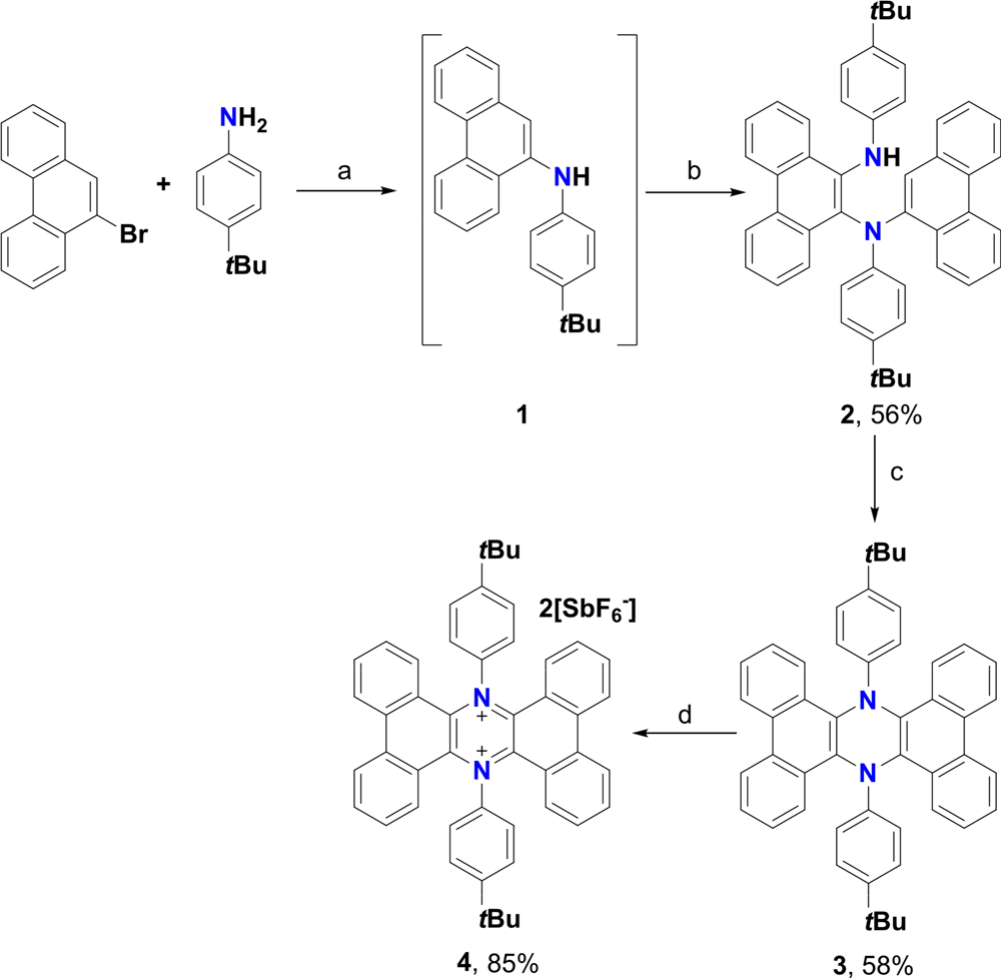
Synthetic Strategy toward **4** (a) Pd(OAc)_2_, dppf, NaOtBu, toluene, 100 °C, 1 h; (b) air bubbling, toluene, 100 °C, 2 h; (c) DDQ, CH_2_Cl_2_, 0 °C to r.t., 2 h; (d) AgSbF_6_, DCE, 0 °C to r.t., 2 h.

In previous contributions, the synthesis of π-extended diphenyl dihydrophenazines has been achieved successfully by using oxidative couplings of either N,N-diarylphenanthrenediamines or N-arylaniline precursors.^27,28,40,43,44^ Based on this consideration, a similar approach for the synthesis of **2** was envisaged, aiming at a one-pot reaction based on Buchwald–Hartwig cross-coupling to generate intermediate **1** followed by direct oxidation to give **2** (Scheme 1).

**Figure 2:**
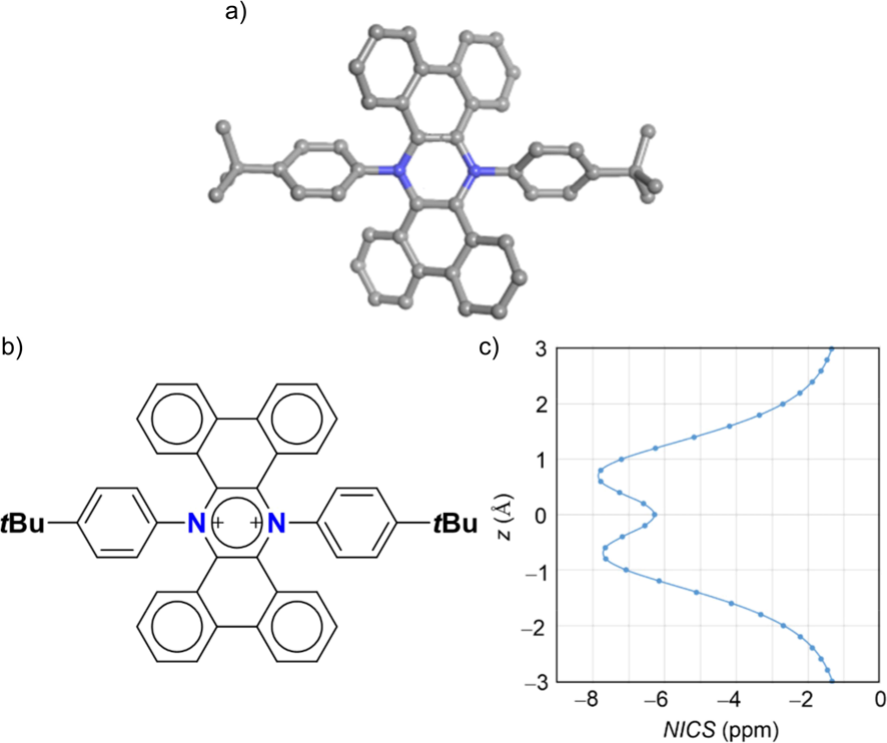
(a) Fully optimized structure (B3LYP-D3BJ/6-31G* level of theory) of half-chair conformation of **4**; (b) molecular scheme of **4** with Clar fully aromatic sextets highlighted; (c) distance in the vertical *z* direction above the central ring of **4** vs NICS values (ppm).

Given the electron-rich nature of **1** and, particularly, of position 10 on the phenanthrene system, obtaining the desired reaction using air as the oxidant seemed a rather convenient option, especially considering that different C–N bond formation reactions involving air (or O_2_) and bases (such as KOtBu) are reported in the literature.^45^ This presents two clear advantages: (i) it allows to generate a complex molecular structure starting from commercially available precursors in a single step and (ii) it avoids the addition of other reagents exploiting the presence of NaOtBu used in the cross-coupling step. At this point, the synthesis started with Buchwald–Hartwig coupling between 9-bromophenanthrene and 4-*t*Bu aniline, resulting in the quantitative formation of **1** after only 1 h (Scheme 1). Bubbling dry air in the reaction mixture at 100 °C smoothly afforded derivative **2** in a 56% yield. Derivative **2** was then treated with 1 equiv of 2,3-dichloro-5,6-dicyano-1,4-benzoquinone (DDQ) in anhydrous degassed CH_2_Cl_2_, giving derivative **3** in a satisfactory 58% yield. To determine the feasibility of the oxidation to dication **4**, cyclovoltammetry experiments were performed in CHCl_3_, resulting in two reversible oxidation waves (+0.04 and +0.27 V vs Fc/Fc^+^, respectively), suggesting that the dicationic derivative **4** could indeed be generated by employing AgSbF_6_ as the oxidant (+0.65 V vs Fc/Fc^+^ in CH_2_Cl_2_).^46^ Because aromaticity is an important factor in determining the stability of PAHs, density functional theory (DFT) studies at the B3LYP-D3BJ/6-31G* level of theory were carried out on the half-chair conformer of **4** (Figure 2a), which, despite some distortion arising from the steric hindrance, was found to be ca. 17 kcal/mol more stable than the boat one (see Figure S34). This investigation was carried out based on geometric, energetic, and magnetic criteria in order to provide a reliable evaluation of the system’s aromatic nature. Considering geometric criteria, DFT-NBO (natural bonding orbital)^47,48^ calculations based on the Bird equation^49,50^ gave an *I_6_* value of aromaticity of 96 (benzene = 100) based on bond order equalization, while the Harmonic Oscillator Model of Aromaticity (HOMA) descriptor^51−53^ resulted in a value of 69. This value is substantially lower than the value computed according to the Bird equation and reflects the departure of the C–N and C–C bond distances in **4** from the optimal values expected for a standard planar aromatic nitrogen-containing heterocycle, rather than a loss of aromaticity. For this reason, in this case, a bond order-based aromaticity index, such as *I*_6_, less dependent on parametric terms, is more appropriate for the quantification of aromaticity, and thus more reliable. Moving to magnetic criteria,^54^ the evolution of the Nucleus Independent Chemical Shift (NICS)^55,56^ was calculated along the Bq points within the axis perpendicular to the average molecular plane that intersects the ring point of electron density at the central charged ring. The analysis of the profile of the NICS vs *z* curve (Figure 2c) shows a strong diamagnetic shielding effect at *z* = 0, with two maxima of −8 ppm at ca. 0.75 Å above and below the average molecular plane. This latter value is close to the covalent radii of carbon and nitrogen and supports a π_2_-aromatic nature, characterized by two diamagnetic ring currents^57,58^ circulating above and below the molecular plane of dication **4**. In contrast, a similar analysis of the NICS profile for **3** (see Figure S38) shows an antiaromatic nature of the 1,4-dihydropyrazine moiety with positive NICS values in the proximity of the ring plane. Finally, also energetically, dication **4** proved to be aromatic, with an estimated aromatic resonance energy of 32.4 (internal energy) or 33.3 (Gibbs energy) kcal/mol. These values, despite being lower than the adiabatic resonance energy calculated for benzene (61.4 kcal/mol with the 6-31G* basis set),^58^ are still indicative of an aromatic system presenting a good degree of stabilization.

**Figure 3:**
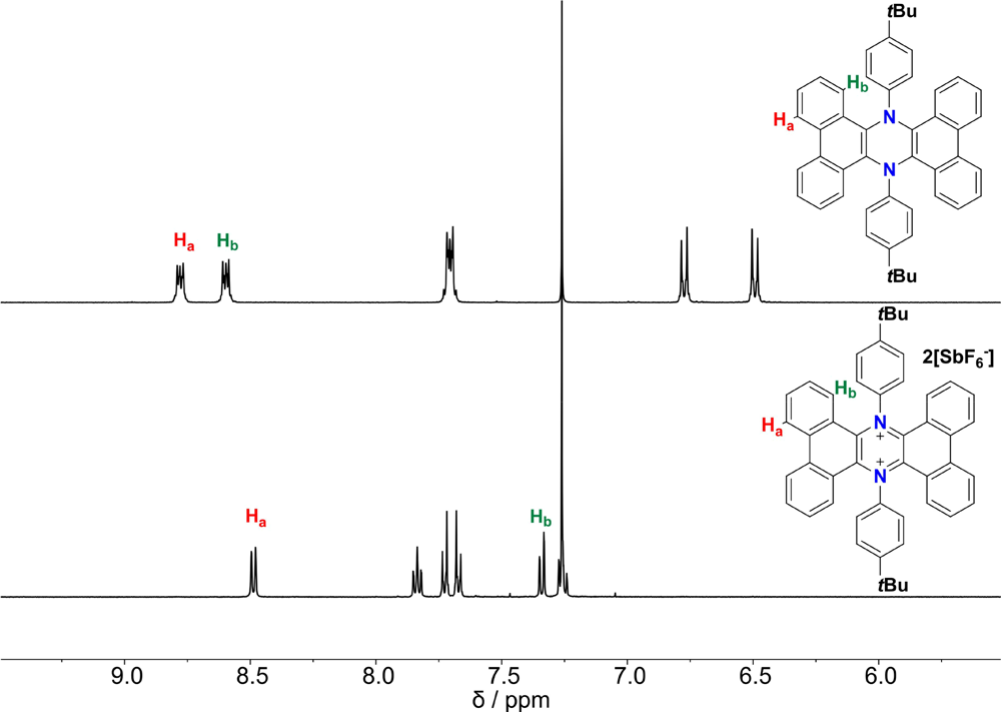
^1^H-NMR (CDCl_3_, 400 MHz, r.t.) comparison of the aromatic regions of **3** and **4**.

Having assessed that **3** can undergo two-electron oxidation in the presence of AgSbF_6_ and that the resulting dication **4** is indeed an aromatic fully benzenoid species (Figure 2b), oxidation was then carried out by treating **3** with 2 equiv of oxidant in dry degassed 1,2-dichloroethane (DCE), resulting in a deep blue solution. As reported for similar systems,^13^ the purification procedure consisted of filtration to remove silver and the precipitation from petroleum ether (PE) to get rid of unreacted materials, affording **4** in a high 85% yield as a clean product. Interestingly, decreasing the amount of oxidants employed only resulted in mixtures of **3** and **4**, preventing the observation and isolation of the radical cation intermediate. As such, the conformation of the radical cation species was studied computationally and is reported in the Supporting Information (see Figure S41). Evidence for the structure of **4** was obtained via ^1^H and ^13^C-NMR, with the first one displaying a highly symmetrical structure and a marked 1.3 ppm upfield shift for the doublet H_b_ (Figure 3). This observation suggests a conformational change in the spatial orientation of the molecule with the H_b_ protons now in the shielding cone of the aniline phenyl rings. When ^13^C-NMR is considered, a signal at 158.8 ppm is visible (whereas in **3**, the most downfield signal was found at 146.2 ppm), which is ascribable to the C=N^+^ carbon atoms in the central charged ring because of a partial iminium character (see Figure S9).

**Figure 4:**
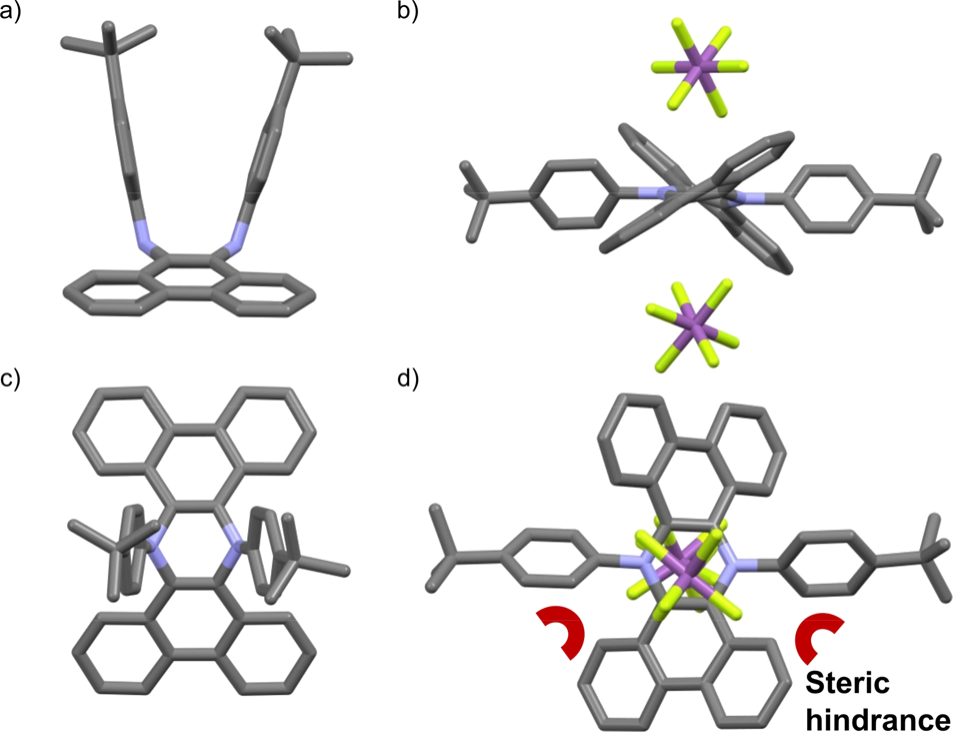
(a) Side and (c) top view of the crystal structure of **3** space group *P* 2_1_/*n* obtained by the diffusion of hexane in DCE; (b) side and (d) top view of the crystal structure of **4** space group *P* 2_1_/*n* obtained by the diffusion of hexane in CHCl_3_, H atoms omitted for clarity, N: blue, F: yellow, and Sb: purple.

To confirm the conformational change occurring upon aromatization of **3**–**4**, crystals suitable for X-ray diffraction were grown for both molecules by the diffusion of hexane in DCE and CHCl_3_, respectively. The resulting molecular structures are displayed in Figure 4. As reported previously for similar extended structures,^27,28^**3** presents a spatial arrangement with the aniline phenyl rings protruding on the same side of the molecule because of the sp^3^ hybridization of the N atoms (Figure 4a,c). Moreover, the extended π system is bent, with a 134° angle and a 1.90 Å deviation from planarity, resulting in a roof-like arrangement. Upon oxidation with AgSbF_6_, a marked conformational change is observed, with the aniline phenyl rings pushed in the plane (as suggested by previous ^1^H-NMR observations) because of the switch to sp^2^ hybridization of the N atoms (Figure 4b,d).

This arrangement generates a highly hindered structure because of the presence of H_b_ and the aniline ring, ultimately resulting in the twisting of the π system observed. Furthermore, the SbF_6_^–^ counterions sit atop the central phenazine ring, confirming the doubly charged nature of the molecule. When bond lengths are considered, the C–N and C–C bonds in the central ring are 1.37 and 1.42 Å long, respectively, whereas in **3**, these values are 1.44 and 1.36 Å. This comparison suggests that some degree of bond length equalization occurs, with an increase in the double-bond character for the C–N bond and a decrease for the C–C bond. In fact, the bond lengths in **4** are much closer to the ones obtained for fully aromatic systems such as benzene (1.40 Å) and pyridine (C–N bond 1.34 Å), with some slight deviation possibly related to the distortion of the system.

**Figure 5:**
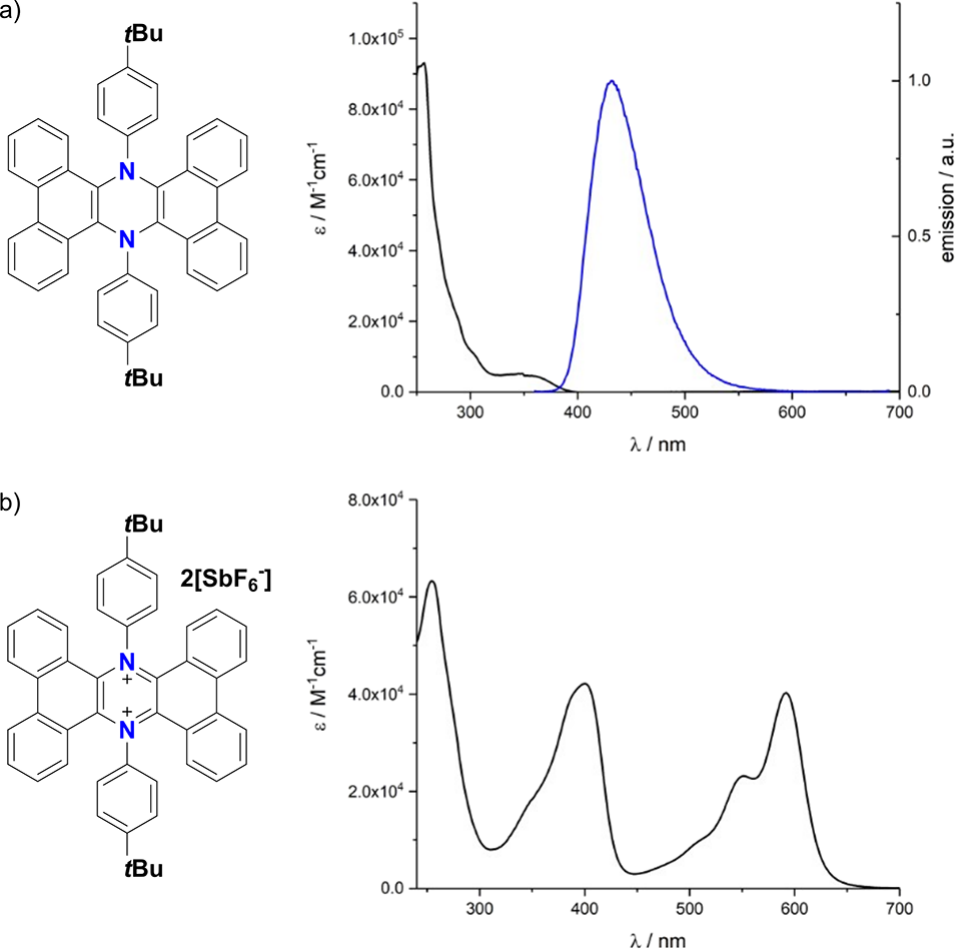
(a) Absorption (black) and fluorescence spectra (blue) of **3** in air-equilibrated CHCl_3_ at room temperature. λ_ex_ = 350 nm; (b) absorption spectrum of **4** in air-equilibrated CHCl_3_ at room temperature.

These results are consistent with the formation of an aromatic 6 π electron system, as suggested by theoretical studies. The formation of a charged extended conjugated system is also proven by a redshifted band in the absorption of **4** (1.45 eV) when compared to **3** (Figure 5). Moreover, the oxidation of **3** is associated with a complete loss in the fluorescence emission of **4**.

**Scheme 2:**
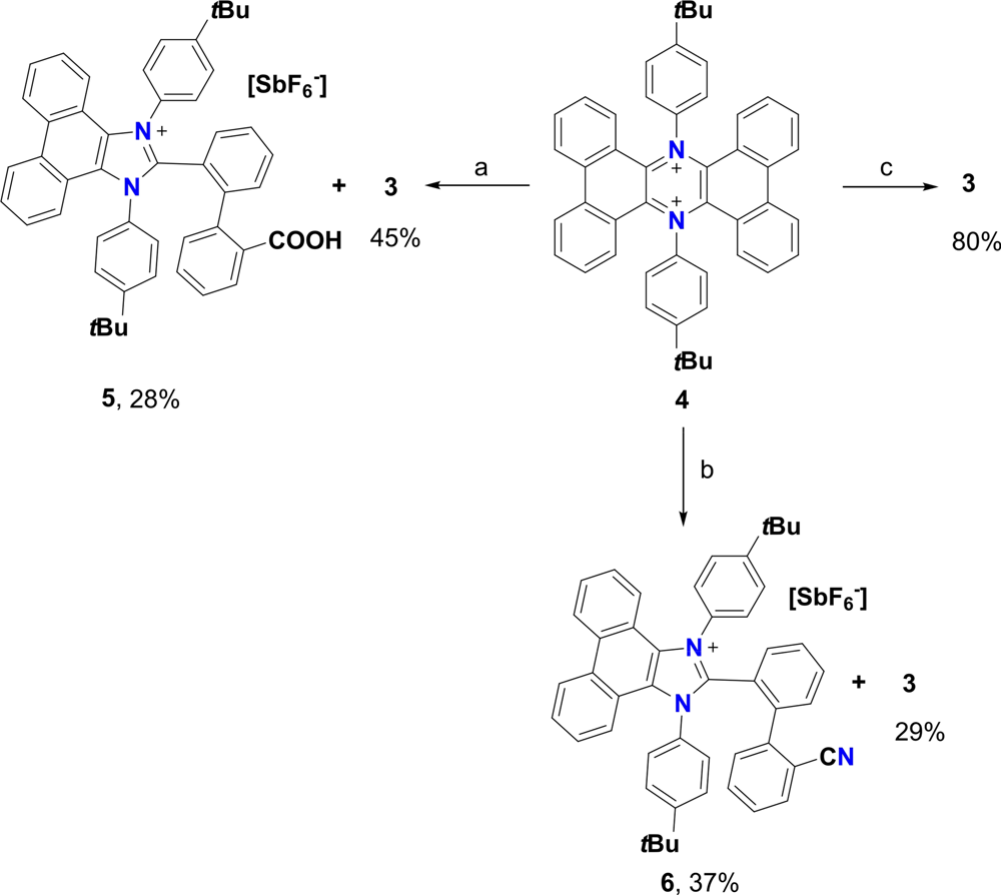
Synthetic Strategy toward **5** and **6** (a) H_2_O, CH_3_CN, r.t., 2 h; (b) NaN_3_, CH_3_CN, r.t., 2 h; (c) PPh_3_, CH_2_Cl_2_, r.t., 2 h.

The aromaticity of the central ring along with the presence of an armchair periphery and a fully benzenoid Clar resonance structure could be at the root of the successful isolation of **4**. Moreover, the small ΔE observed between the first and second oxidation of **3** (see Figure S33) seems to suggest reduced Coulombic repulsion between charges. In fact, from electrostatic potential and collective NBO charge calculations performed on **4** (see Figure S39), an efficient charge delocalization on the aromatic scaffold was demonstrated, thus giving theoretical confirmation to the experimental results. Given the electron-poor and distorted nature of **4**, it could be expected for this molecule to be highly sensitive toward nucleophiles and reductants, as reported in less-extended examples.^42^ Instead, **4** proved to be stable for days in the solid state and for hours in chlorinated solvents, with a half-life in air-equilibrated CHCl_3_ of more than 12 h for a 2 × 10^–5^ M solution (see Figure S21). On the other hand, **4** proved to be much more sensitive in polar hydrophilic solvents, suggesting that the observed stability in the chlorinated solvents is more related to the reduced water content than to specific solvent effects. To prove this, time-dependent absorption spectra were performed on a 2 × 10^–5^ M solution of **4** in CH_3_CN, which resulted in almost immediate degradation. The measurement was then repeated using dry CH_3_CN, and this time, complete disappearance of **4** absorption bands occurred in less than 2 h (with the cuvette open to air, Figure S22). These results strongly suggest that because of the electron-poor and distorted nature of **4**, a nucleophilic attack by water molecules can occur, resulting in the observed degradation. To confirm this assumption and study the degradation products, a batch reaction was carried out on **4** in CH_3_CN by adding 100 μL of water to the solution (Scheme 2). As expected, **4** underwent complete degradation, resulting in the isolation of **3** (45% yield) and, surprisingly, the singly charged imidazolinium derivative **5** (28% yield).

**Figure 6:**
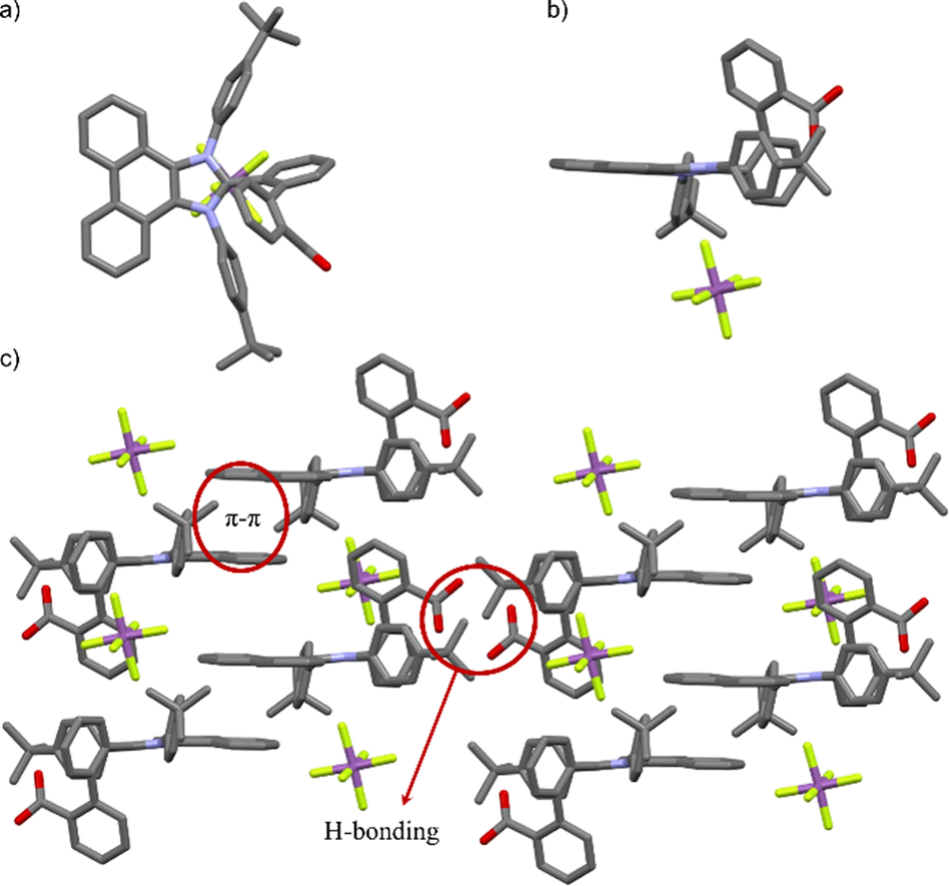
(a) Top and (b) side view of the crystal structure of **5** space group: *P*-1, obtained from the diffusion of hexane in DCE; H atoms omitted for clarity, N: blue, O: red, F: yellow, and Sb: purple; (c) details of the crystal packing of **5** with highlighted intermolecular interactions.

Derivative **5** was characterized completely via NMR, UV–vis, and high-resolution mass spectrometry (HRMS) (see Figures S12–S17), and its structure was confirmed by X-ray diffraction (Figure 6a,b). The presence of a signal at 169.5 and 157.0 ppm in the ^13^C-NMR is ascribable to the carboxylic group and the carbon atom comprised between the two nitrogen atoms of the imidazolinium system. This was undoubtedly confirmed by X-ray structure determination, indeed proving the presence of the expected groups and highlighting the extended imidazolinium structure of **5**. More in detail, the imidazolinium core presents bond equalization, as expected for an aromatic system with C–N bonds of 1.40 and 1.34 Å, and a C–C bond of 1.38 Å. Interestingly, the crystal structure presents couples of molecules held together by π-π interactions arising from the phenanthrene units, and each dimer interacts with the neighboring ones via H bonding provided by the carboxylic groups (Figure 6c).

The reaction of **4** to give **5** represents only the second known synthesis of a phenanthrene-extended triarylimidazolinium ion.^59^ This kind of transformation from a dicationic six-member aromatic ring to a cationic five-member aromatic ring is unprecedented to the best of our knowledge and is highly interesting, especially considering that this process arises from the rupture of a C–C double bond. Because extended imidazolinium ions represent a valuable class of molecules because of their potential applications in optoelectronics and sensing,^59−61^ an investigation on the reaction was carried out. From this point of view, it is reasonable to infer that water can attack the pseudo iminium C=N^+^ bond, leading to the observed product. This assumption is supported by the high lowest unoccupied molecular orbital (LUMO) coefficients on the carbon atom next to the charged nitrogen, as highlighted in the Kohn–Sham orbitals calculated at the B3LYP-D3BJ/6-31G* level of theory (see Figure S36). Moreover, the distortion in the system can facilitate the double-bond rupture, resulting in the formation of **5**. To confirm the origin of the two oxygen atoms in the carboxylic group, a degradation experiment using H_2_^18^O was performed (see Figure S23), and from the HRMS of the reaction mixture, the signal corresponding to derivative **5** presenting two ^18^O atoms was clearly visible as the main peak at *m/z*: 683.3392 (calc. For C_48_H_43_N_2_^18^O_2_: 683.3404). This suggests that two molecules of water are involved in the degradation reaction, with two attacks occurring on the same carbon. To rule out other products and understand the precise stoichiometry of the transformation, the reaction was studied via ^1^H-NMR by treating **4** with 10 μL of D_2_O in acetone-*d*6 and using 1 μL of dioxane as the internal standard. From these experiments, a ratio of 1:1 between **3** and **5** was observed, with a yield of 50% each, and no other products were visible, suggesting an overall degradation yield >95% (see Figures S24–26). This result further confirms that the two attacks by water molecules must occur on the same carbon (and not on different sites of **4**). To shed light on the role of the nucleophile in degradation, an alternative batch reaction was performed by treating **4** with NaN_3_ in CH_3_CN. In this case, a 29% yield of **3** was obtained, along with a different product in a 37% yield, which, after NMR and HRMS characterization, was found to be molecule **6** (Scheme 2). Interestingly, the latter is structurally very similar to **5;** however, it presents a CN group in place of the COOH. Finally, to disclose the effect of steric hindrance and the nature of the nucleophile, the reaction was repeated by treating **4** with PPh_3_ in CH_2_Cl_2_, which resulted in the recovery of **3** in an 80% yield, suggesting an enhancement of the reductive pathway. This reaction, studied via NMR using CDCl_3_ as the solvent, resulted in the exclusive formation of **3** with a yield >95%. This result suggests that in the presence of hindered, easily oxidizable nucleophiles, dication **4** is preferentially reduced to **3**, while the conversion to the imidazolinium derivatives occurs only in the presence of small and less oxidizable nucleophiles. This result is important also for potential molecular switch applications of these derivatives because by using PPh_3_ as the reductant, it is possible to make the conversion and conformational change between **3** and **4** reversible on multiple cycles (see Figures S28–S30).

## Conclusions

The obtained results represent an important extension of knowledge on doubly charged PAHs and constitute a clear example of how aromaticity can induce not only conformational changes but also variations in the reactivity of systems. Indeed, upon two-electron oxidation, **3** underwent an aromatization reaction, which resulted in a π-extended dication. The latter could be isolated and studied, highlighting a dramatic change in conformation. Moreover, the presence of the doubly charged 6 π electron system resulted in a peculiar reactivity, with **4** undergoing a room temperature ring contraction and/or reduction to **3**, depending on the nucleophile used. These results not only pave the way for future applications of these systems as molecular actuators but also represent a new strategy to prepare functionalized π-extended triarylimidazolinium systems, potentially useful for light emission and sensing devices.

## Supporting Information Available

Detailed experimental procedures, spectra (NMR, HRMS, and UV–vis), NMR experiments, X-ray diffraction data, and calculations (PDF)

## Accession Codes

CCDC 2129306–2129309 contain the supplementary crystallographic data for this paper. These data can be obtained free of charge via www.ccdc.cam.ac.uk/data_request/cif, or by emailing data_request@ccdc.cam.ac.uk, or by contacting The Cambridge Crystallographic Data Centre, 12 Union Road, Cambridge CB2 1EZ, UK; fax: +44 1223 336033.
