# Supplementary material for: Phenanthrene-Extended Phenazine Dication: An Electrochromic Conformational Switch Presenting Dual Reactivity
Source: J Am Chem Soc. 2022 Apr 12;144(16):7295–301. doi: 10.1021/jacs.2c00493 (PMC9052754; doi:10.1021/jacs.2c00493)
Supplement: Supplementary file 1 — ja2c00493_si_001.pdf [file ja2c00493_si_001.pdf]

Supporting Information

Phenanthrene extended phenazine dication: an electrochromic conformational switch presenting dual reactivity

Jacopo Dosso*,§, Beatrice Bartolomei§, Nicola Demitri#, Fernando P. Cossio*,ǂ,†, Maurizio Prato*,§,ǁ,⊥

§ Department of Chemical and Pharmaceutical Sciences, CENMAT, Centre of Excellence for Nanostructured Materials, INSTM UdR Trieste, University of Trieste, via Licio Giorgieri 1, 34127 Trieste, Italy.

ǁ Centre for Cooperative Research in Biomaterials (CIC BiomaGUNE), Basque Research and Technology Alliance (BRTA), Paseo de Miramón 194, 20014, Donostia San Sebastián, Spain

⊥ Basque Fdn Sci, Ikerbasque, 48013 Bilbao, Spain.

Elettra—Sincrotrone, Trieste S.S. 14 Km 163.5, Area Science Park, 34149 Basovizza, Trieste (Italy)

† Departamento de Química Orgánica I and Instituto de Innovaciónen Química Avanzada (ORFEO-CINQA) University of the Basque Country (UPV/EHU), Paseo Manuel Lardizabal 3, 20018 Donostia/San Sebastián (Spain).

ǂ Donostia International Physics Center (DIPC) Paseo Manuel Lardizabal 4, 20018 Donostia/San Sebastián (Spain).

Summary

1. General Remarks 2
- **1.1 Instrumentation** 2
- **1.2 Materials and methods** 3

2 Synthetic procedures and spectral data 3
- **2.1 Synthesis of 2** 3
- **2.2 Synthesis of 3** 4
- **2.3 Synthesis of 4** 5
- **2.4 Synthesis of 5** 5
- **2.5 Synthesis of 6** 6
- **2.6 4 reduction reaction** 7

3 NMR and HRMS spectroscopic characterization 8
- **3.1 Characterization of 2** 8
- **3.2 Characterization of 3** 10
- **3.3 Characterization of 4** 12
- **3.4 Characterization of 5** 14
- **3.5 Characterization of 6** 17

4 UV-Vis stability studies 19

5 $H_2{}^{18}O$ Degradation experiment 20

6 NMR degradation experiment 21

7 Photochemical characterizations 25

8 Electrochemical characterization 27

9 Calculations 28

10 X-Ray Crystallography 52

11 References 56

1. General Remarks

1.1 Instrumentation

Thin layer chromatography (TLC) was conducted on Sigma Aldrich pre-coated aluminum sheets (0.25 mm layer thickness, 60 Å porosity and fluorescent indicator GF254) and were visualized using 254 or 365 nm light. Flash column chromatography was carried out using Merck Gerduran silica gel 60 (particle size 40 63 μm). ***Melting points*** (M.P.) were measured on a Gallenkamp apparatus. All of melting points have been measured in open capillary tubes and have not been corrected. ***Nuclear magnetic resonance*** (NMR) ^{1}H, and ^{13}C spectra were obtained on Varian Inova spectrometer (500 MHz ^{1}H and 126 MHz ^{13}C) or Varian 400 MHz NMR spectrometer (400 MHz ^{1}H and 101 MHz ^{13}C). Chemical shifts were reported in ppm according to tetramethylsilane using the solvent residual signal as an internal reference ($CDCl_3$: δ_H = 7.26 ppm, δ_C = 77.16 ppm, CD_2Cl_2: δ_H = 5.32 ppm, δ_C = 53.84 ppm, MeOD: δ_H = 3.31 ppm, δ_C = 49.00 ppm, CD_3CN: δ_H = 1.94 ppm, δ_C = 1.32, 118.26 ppm, Acetone-*d6*: δ_H = 2.05 ppm, δ_C = 29.84, 206.26 ppm). Coupling constants (*J*) were given in Hz and were averaged. Resonance multiplicity was described as s (singlet), d (doublet), t (triplet), m (multiplet), br (broad signal), dd (doublet of doublets), dt (doublet of triplets). Carbon spectra were acquired with a complete decoupling for the proton, unless specified. All spectra were recorded at 25 °C unless specified. ***Infrared spectra*** (IR) were recorded on a Shimadzu IR Affinity 1S FTIR spectrometer in ATR mode with a diamond mono-crystal. Selected absorption bands are reported in wavenumber (cm^{-1}). ***ESI-High resolution mass spectrometry*** (ESI-HRMS). ESI-HRMS was performed at University of Trieste Chemistry department, High resolution mass spectra (HRMS) were obtained on Bruker micrOTOF-Q (ESI-TOF). ***Photophysical analysis***: Absorption spectra of compounds were recorded on air equilibrated solutions at room temperature with an Agilent Cary 5000 UV-Vis spectrophotometer, using quartz cells with path length of 1.0 cm. Emission spectra were recorded on an Agilent Cary Eclipse fluorescence spectrofluorometer. Emission measurements were performed on an Edinburgh instruments FS5 spectrofluorometer using a 150 W CW Ozone-free xenon arc lamp as source and a Photomultiplier R928P (spectral coverage 200 nm – 900 nm, cooled and stabilised) as detector. Quantum yields were performed using the integrating sphere setup SC-30 on a sample solution in a quartz cuvette and using the same solvent in another cuvette as reference. Luminescence lifetimes were measured with an Edinburgh Instruments FS5 time-correlated single-photon counting spectrofluorimeter, exciting the sample at 280 nm with a picosecond pulsed diode laser (EPLED -280 Edimburgh Instruments). ***X-Ray Data***: CCDC **2129306**, **2129307**, **2129308** and **2129309** contain the supplementary crystallographic data for **3a** (triclinic crystal form of **3**), **3b** (monoclinic crystal form of **3**), **4** and **5**. Related files can be obtained free of charge from The Cambridge Crystallographic Data Centre via https://www.ccdc.cam.ac.uk/structures.

1.2 Materials and methods

Chemicals were purchased from Sigma Aldrich, TCI, Alfa Aesar and Fluorochem and were used as received unless otherwise stated. Solvents were purchased from Sigma Aldrich and Alfa Aesar, while deuterated solvents from Eurisotop and Sigma Aldrich. Anhydrous conditions were achieved by repeated cycles of flaming with a heat gun under vacuum and purging with Argon (Ar). The inert atmosphere was maintained using Argon-filled balloons equipped with a syringe and needle that was used to penetrate the silicon stoppers used to close the flask's necks. Additions of liquid reagents were performed using plastic syringes. Degassing of solutions was performed by bubbling argon in the reaction under sonication for at least 10 minutes. Dry solvents were obtained commercially or via treatment on activated molecular sieves (MS, 3Å for CH_3CN). Activation of MS was carried out by multiple cycles of heating under vacuum and the solvents were left over MS for at least 24 h. MilliQ water was obtained from a Millipore Milli-Q Plus 185 apparatus and presented a resistivity of 18.2 MΩcm. MilliQ water was always used unless otherwise specified.

2 Synthetic procedures and spectral data

2.1 Synthesis of 2

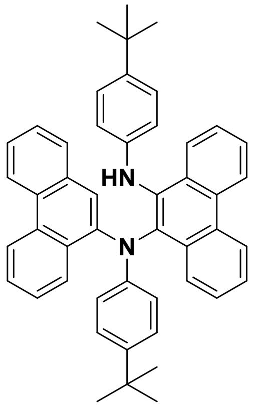

In a double necked flask, 9-bromophenanthrene (500 mg, 1.95 mmol), 4-tert-butyl aniline (0.37 ml, 2.33 mmol), $Pd(OAc)_2$ (22 mg, 0.1 mmol) and dppf (100 mg, 0.2 mmol) were added and purged with Ar (3 × vacuum/Ar cycles). Anhydrous toluene (10 mL) was then added, and the resulting suspension degassed for 10 min. with Ar bubbling and sonication. NaOtBu (412 mg, 4.2 mmol) was then added in one portion under Ar and the reaction degassed for further 10 min. The resulting dark suspension was then placed in a preheated silicon oil bath at 100°C and stirred for 2 h at the same temperature. At this point, a balloon equipped with a 12 cm needle was used to bubble air (passed on a column filled with drierite™) in the reaction solution at 100°C for 2 h. After complete conversion of the intermediate from the previous step, the reaction was diluted with CH_2Cl_2 (50 mL) and washed with water (2 × 50 mL) and brine (50 mL). The resulting organic solution was dried over $MgSO_4$, filtered, and evaporated. The obtained residue was then purified via two consecutive Flash column chromatography on silica plug (CH_2Cl_2/PE 1/9 and CH_2Cl_2/PE 0.5/9.5) to give **2** as a fluffy white solid (355 mg, 56% yield).

M.P.:196-198°C. ^{1}H NMR (500 MHz, CD_2Cl_2) δ: 8.79 (d, *J* = 8.3 Hz, 2 H), 8.70 (d, *J* = 8.3 Hz, 1 H), 8.55 (d, *J* = 8.3 Hz, 1 H), 8.17-8.13 (m, 2 H), 7.97 (d, *J* = 8.3 Hz, 1 H), 7.69-7.65 (m, 2 H), 7.61 (t, *J* = 7.6 Hz, 1 H), 7.54-7.47 (m, 3 H), 7.43 (t, *J* = 7.6 Hz, 1 H), 7.38-7.35 (m, 2 H), 7.30 (d, *J* = 7.6 Hz, 1 H), 7.16 (dd, *J* = 8.6, 2.5 Hz, 1 H), 7.12 (dd, *J* = 8.6, 2.5 Hz, 1 H), 6.86 (d, *J* = 6.7 Hz, 2 H), 6.74 (dd, *J* = 8.6, 2.5 Hz, 1 H), 6.60 (dd, *J* = 8.6, 2.5 Hz, 1 H), 6.12 (d, *J* = 6.7 Hz, 2 H), 5.83 (s, 1 H), 1.24 (s, 9 H), 1.15 (s, 9 H). ^{13}C NMR (101 MHz, $CDCl_3$) δ: 146.5, 143.5, 143.4, 141.8, 140.2, 135.84, 135.75, 132.6, 132.2, 132.0, 130.5, 130.4, 128.8, 128.7, 128.0, 127.4, 126.9, 126.79, 126.77, 126.6, 126.53, 126.50, 126.1, 126.0, 125.9, 125.6, 125.4, 125.2, 123.63, 123.58, 123.0, 122.9, 122.4, 118.5, 118.2, 115.1, 34.2, 33.9, 31.6. IR (ATR) ν (cm^{-1}): 3379, 2953, 2913, 2849, 1717, 1609, 1593, 1510, 1491, 1464, 1449, 1422, 1362, 1321, 1294, 1256, 1190, 1109, 1078, 1040, 864, 824, 760, 746, 725, 658, 617. ESI-HRMS: $[M+H]^+$ calc. for $[C_{48}H_{44}N_2Na]^+$: 671.3397; found 671.3397.

2.2 Synthesis of 3

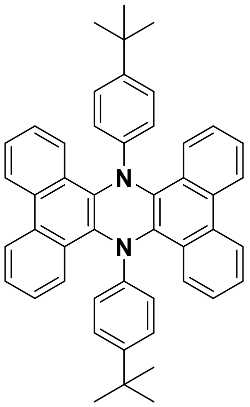

In a double necked flask, **2** (200 mg, 0.31 mmol) was added and purged with Ar (3 × vacuum/Ar cycles). Dry CH_2Cl_2 (20 mL) was then added, and the resulting solution degassed 10 min. by bubbling Ar under sonication. The reaction was then cooled to 0°C with an ice bath and DDQ (70 mg, 0.31 mmol) was added in one portion. The resulting dark solution was stirred at the same temperature for 10 min., allowed to reach r.t. and stirred for 2 h. The reaction was then filtered on a silica plug using CH_2Cl_2 (100 mL). The organic phase was then evaporated to dryness, reprecipitated and centrifuged (6000 rpm for 10 min.) from EtOH (3 times), and PE (3 times) to give **3** as a white solid (116 mg, 58% yield). (On larger scales purification was performed *via* filtration on glass frit filter using the same solvents.)

M.P.:>300°C. ^{1}H NMR (400 MHz, $CDCl_3$) δ: 8.79-8.77 (m, 4 H), 8.61-8.59 (m, 4 H), 7.73-7.68 (m, 8 H), 6.77 (d, *J* = 8.9 Hz, 4 H), 6.49 (d, *J* = 8.9 Hz, 4 H), 1.03 (s, 18 H). ^{13}C NMR (101 MHz, $CDCl_3$) δ: 146.2, 142.4, 142.1, 130.9, 129.8, 127.3, 126.9, 125.0, 124.9, 123.1, 115.4, 33.8, 31.4. IR (ATR) ν (cm^{-1}): 3063, 2959, 2901, 2866, 1611, 1587, 1510, 1495, 1449, 1422, 1393, 1362, 1348, 1329, 1315, 1287, 1273, 1244, 1200, 1155, 1117, 1076, 1038, 1013, 1001, 953, 932, 868, 837, 822, 806, 785, 773, 760, 725, 714, 654, 640, 615. ESI-HRMS: $[M+H]^+$ calc. for $[C_{48}H_{43}N_2]^+$: 647.3421; found 647.3422.

2.3 Synthesis of 4

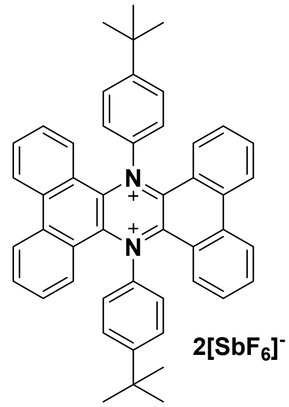

In a flame dried double necked flask, **3** (50 mg, 0.08 mmol) was added together with $AgSbF_6$* (54 mg, 0.16 mmol) and purged with Ar (3 × vacuum/Ar cycles). Dry DCE (10 mL) was then added under Ar at 0 °C. The resulting solution undergoes a colour change upon addition of solvent, turning dark blue. After 2 h stirring at r.t. the reaction was filtered using a 0.1 µm PTFE membrane to remove the Ag^0 precipitate and evaporated. The resulting dark solid was dissolved in a small amount of dry DCE and reprecipitated from hexane. This procedure was repeated three times each one followed by centrifugation (6000 rpm, 10 min.) to give **4** as a dark blue solid (76 mg, 85% yield).

* $AgSbF_6$ is hygroscopic, for this reason it was weighted in a vial under Ar and added immediately to the flask.

M.P.:>300°C. ^{1}H NMR (400 MHz, CD_2Cl_2) δ: 8.56 (d, J = 8.2 Hz, 4 H), 7.90 (ddd, J = 8.2, 6.7, 1.3 Hz, 4 H), 7.74 (d, J = 9.0 Hz, 4 H), 7.69 (d, J = 9.0 Hz, 4 H), 7.35-7.28 (m, 8 H), 1.43 (s, 18 H). ^{13}C NMR (126 MHz, $CDCl_3$) δ: 158.8, 141.9, 140.7, 135.7, 135.4, 131.0, 129.5, 128.7, 128.5, 125.1, 120.2, 35.8, 31.3. IR (ATR) ν (cm^{-1}): 3063, 2963, 2872, 1595, 1501, 1456, 1439, 1377, 1335, 1315, 1294, 1271, 1254, 1190, 1157, 1142, 1117, 1099, 1024, 1005, 962, 847, 812, 791, 777, 756, 731, 719, 652, 640, 606. UV-Vis ($CHCl_3$): 590 nm (ε 40012 $M^{-1}cm^{-1}$), 400 nm (ε 42111 $M^{-1}cm^{-1}$), 255 nm (ε 59172 $M^{-1}cm^{-1}$). Not stable enough for HR-Mass. ESI-LRMS: $[M]^-$ calc. for $[SbF_6]^-$: 234.9; found 234.6.

2.4 Synthesis of 5

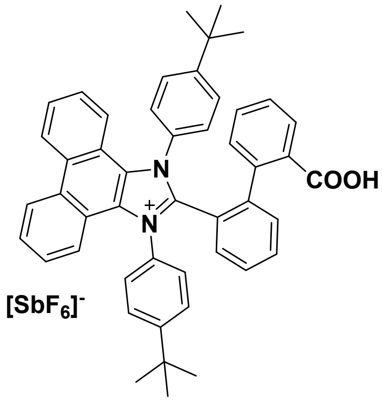

In a single necked flask, **4** (30 mg, 0.027 mmol) was added followed by CH_3CN (10 mL) and milliQ H_2O (100 µL). The solution was stirred under air for 2 h, resulting in the formation of a white suspension which was centrifuged 3 times from CH_3CN, to give **3** (8 mg, 45% yield). The CH_3CN solution (20 mL) was concentred under vacuum then diluted with milliQ H_2O (30 mL) and the suspension extracted with CH_2Cl_2 (20 mL × 3). The organic layers were then evaporated under reduced pressure. The resulting residue was dissolved in CH_2Cl_2 (1 mL) and reprecipitated from PE. This procedure was repeated three times, each one followed by centrifugation (6000 rpm, 10 min.) to give **4** as white powder (7 mg, 28% yield).

NMR experiment: In an NMR tube **4** (1.70 mg, 1.52×10^{-3} mmol)* was added followed by acetone-*d6* and 10 µL D_2O. When the solution turned transparent, 1µL of dioxane was added as internal standard. ^{1}H-NMR analysis gave a 50% yield of **3** and 50% yield of **5**. *Low concentrations were used to assure complete solubility of products.

$H_2{}^{18}O$ Experiment: In a flame dried Schlenk flask, **4** (10 mg, 8.94×10^{-3} mmol) was dissolved in anhydrous CH_3CN (2 mL) and 97% $H_2{}^{18}O$ (50 µL) added immediately. After complete discoloration of the solution a sample was collected, filtered on a 0.45 µm PTFE filter and analysed via HRMS spectroscopy.

5: M.P.:295-297°C. ^{1}H NMR (400 MHz, Acetone-*d6*) δ: 9.06 (d, *J* = 8.4 Hz, 2 H), 8.07 (dd, *J* = 7.8, 1.1 Hz, 1 H), 8.02 (d, *J* = 7.8 Hz, 1 H), 7.81 (t, *J* = 7.7 Hz, 2 H), 7.74 (bs, 4 H), 7.58-7.49 (m, 7 H), 7.44 (td, *J* = 7.7, 1.2 Hz, 4 H), 7.35 (dd, *J* = 7.8, 1.0 Hz, 1 H), 6.95 (bs, 2 H), 1.40 (s, 18 H). ^{13}C NMR (126 MHz, MeOD) δ: 169.5, 157.0, 150.0, 143.0, 140.1, 134.9, 134.4, 133.5, 133.0, 132.9, 132.6, 132.0, 130.3, 129.9, 129.6, 128.9, 128.8, 128.6, 128.2, 128.0, 125.7, 123.1, 121.4, 120.5, 36.0, 31.6. IR (ATR) ν (cm^{-1}): 3067, 2963, 2870, 1705 (s, COOH), 1508, 1472, 1435, 1368, 1269, 1204, 1119, 1105, 1018, 847, 789, 775, 756, 721, 698, 656, 621, 583. UV-Vis ($CHCl_3$): 344 nm (ε 6690 $M^{-1}cm^{-1}$), 293 nm (ε 29834 $M^{-1}cm^{-1}$), 259 nm (ε 108834 $M^{-1}cm^{-1}$). ESI-HRMS: $[M]^+$ calc. for $[C_{48}H_{43}N_2O_2]^+$: 679.3319; found 679.3315

2.5 Synthesis of 6

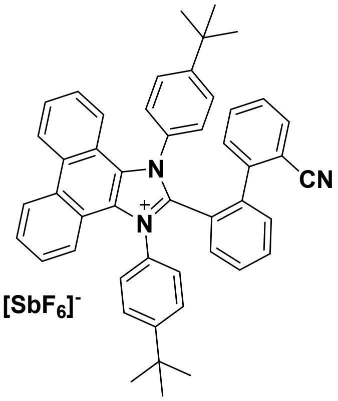

In a flame dried Schlenk flask, **4** (30 mg, 0.027 mmol) was added together with NaN_3 (35 mg, 0.54 mmol) and the solids were kept under vacuum for 20 min. Dry CH_3CN (10 mL) was then added, and the reaction stirred at r.t. for 2 h. The resulting yellowish suspension was diluted with milliQ water (30 mL) and extracted with CH_2Cl_2 (20 mL × 3). The organic layers were washed with milliQ water (10 mL × 3) and the CH_2Cl_2

solution evaporated under reduced pressure. The resulting residue was dissolved in CH_2Cl_2 (1 mL) and reprecipitated from PE. This procedure was repeated three times, each one followed by centrifugation (6000 rpm, 10 min.) to give **6** as a white powder (9 mg, 37% yield). Evaporation of the supernatants gave **3** (5 mg, 29%).

6: M.P.:284-286°C. ^{1}H NMR (400 MHz, $CDCl_3$) δ: 8.81-8.75 (m, 3 H), 8.42 (bs, 2 H), 7.71 (dd, *J* = 7.3, 1.5 Hz, 1 H), 7.68-7.64 (m, 4 H), 7.55-7.43 (m, 5 H), 7.38 (d, *J* = 8.3 Hz, 2 H), 7.31 (t, *J* = 7.8 Hz, 2 H), 7.27 (d, *J* = 7.8 Hz, 1 H), 7.03 (d, *J* = 8.3 Hz, 2 H), 6.64, (bs, 2 H), 1.38 (s, 18 H). ^{13}C NMR (101 MHz, $CDCl_3$) δ: 155.5, 147.9, 142.2, 137.3, 136.7, 134.9, 132.8, 132.3, 131.7, 131.1, 130.9, 129.6, 128.8, 128.7, 128.4, 128.1, 127.8, 127.3, 126.4, 125.5, 124.3, 122.5, 120.6, 120.5, 117.1, 114.4, 110.2, 35.3, 31.3. IR (ATR) ν (cm^{-1}): 3061, 2959, 2926, 2868, 2224 (w, CN), 1508, 1472, 1366, 1269, 1175, 1105, 1051, 1018 ,851 ,806, 760, 741, 721 ,700, 610, 583, 569, 544. ESI-HRMS: $[M]^+$ calc. for $[C_{48}H_{42}N_3]^+$: 660.3373; found 660.3371

2.6 4 reduction reaction

a) Batch reaction

In a flame dried Schlenk flask, **4** (30 mg, 0.027 mmol) was added together with PPh_3 (35 mg, 0.14 mmol) and the solids were kept under vacuum for 20 min. Dry CH_2Cl_2 (10 mL) was then added, and the reaction stirred at r.t. for 2 h. The resulting clear yellow solution was filtered on a silica plug washing abundantly with CH_2Cl_2 and the organic layers were then evaporated under reduced pressure. The resulting residue was reprecipitated from MeOH three times, each one followed by centrifugation (6000 rpm, 10 min.) to give **3** as white powder (14 mg, 80% yield).

b) NMR reaction with PPh_3

In an NMR tube **4** (2.27 mg, 2.03×10^{-3} mmol)* was added together with PPh_3 (2.13 mg, 8.12×10^{-3} mmol) and dissolved in $CDCl_3$. After complete discolouration of the solution, 1μL of dioxane was added as internal standard and ^{1}H-NMR performed, resulting in a reduction yield ≥95%. *Low concentrations were used to assure complete solubility of products.

3 NMR and HRMS spectroscopic characterization

3.1 Characterization of 2

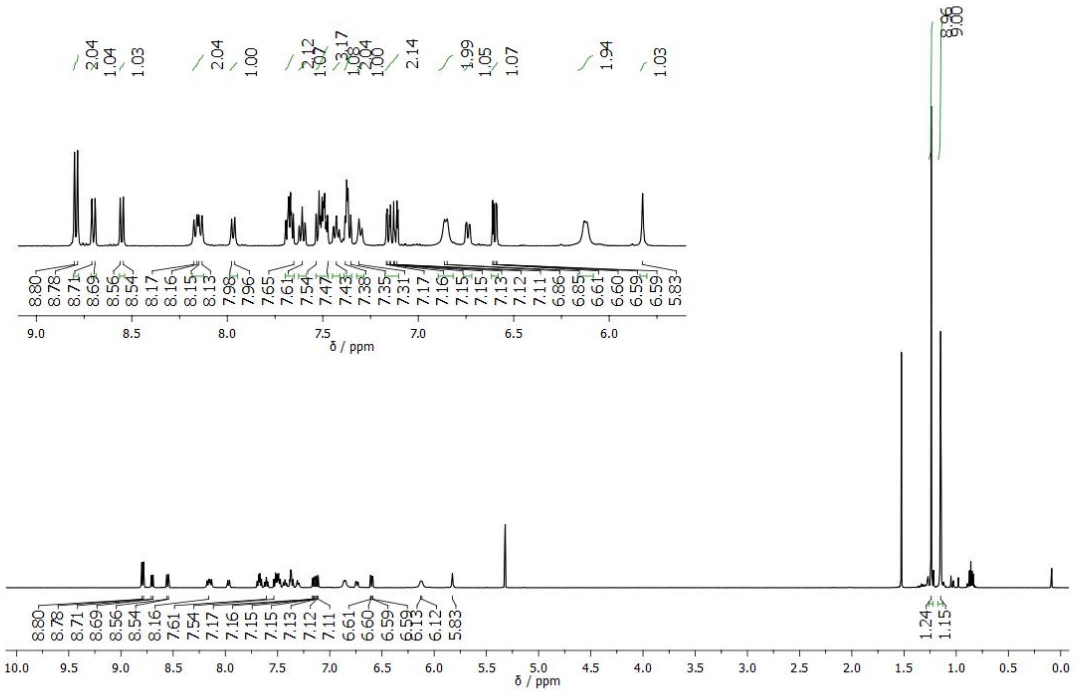

Figure S1: ^{1}H NMR 500 MHz of **2** in CD_2Cl_2.

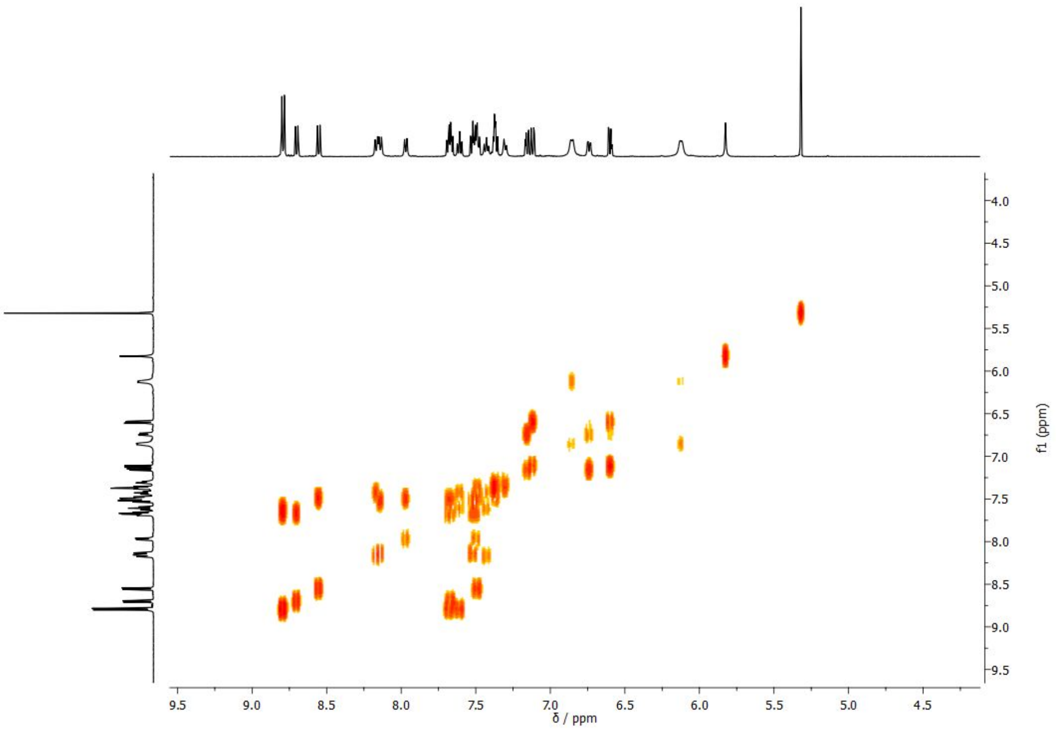

Figure S2: ^{1}H-^{1}H 500 MHz COSY of **2** (aromatic part) in CD_2Cl_2.

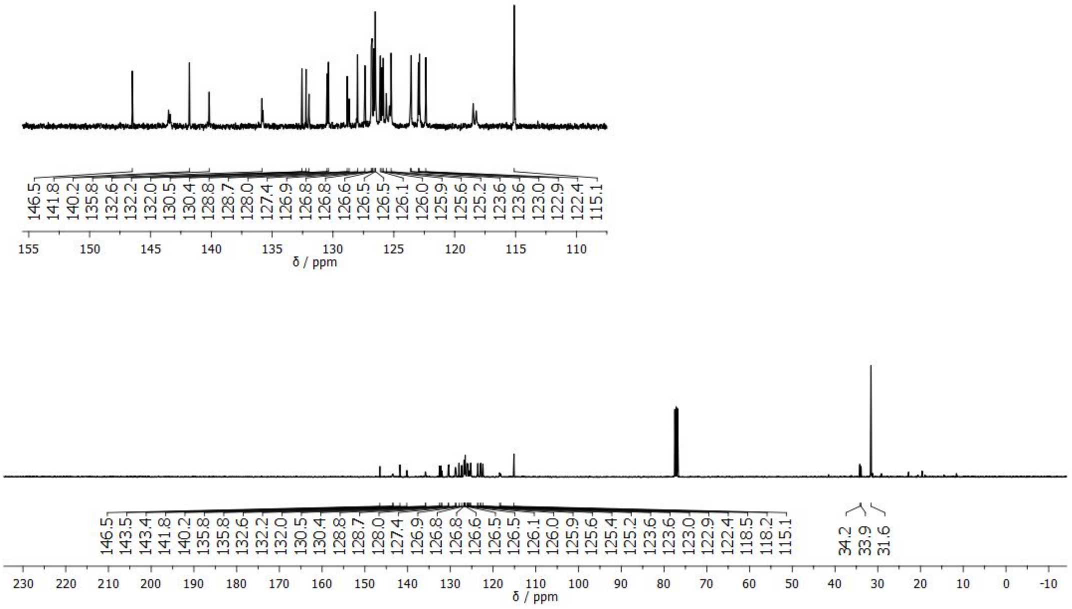

Figure S3: ^{13}C NMR 101 MHz of **2** in $CDCl_3$.

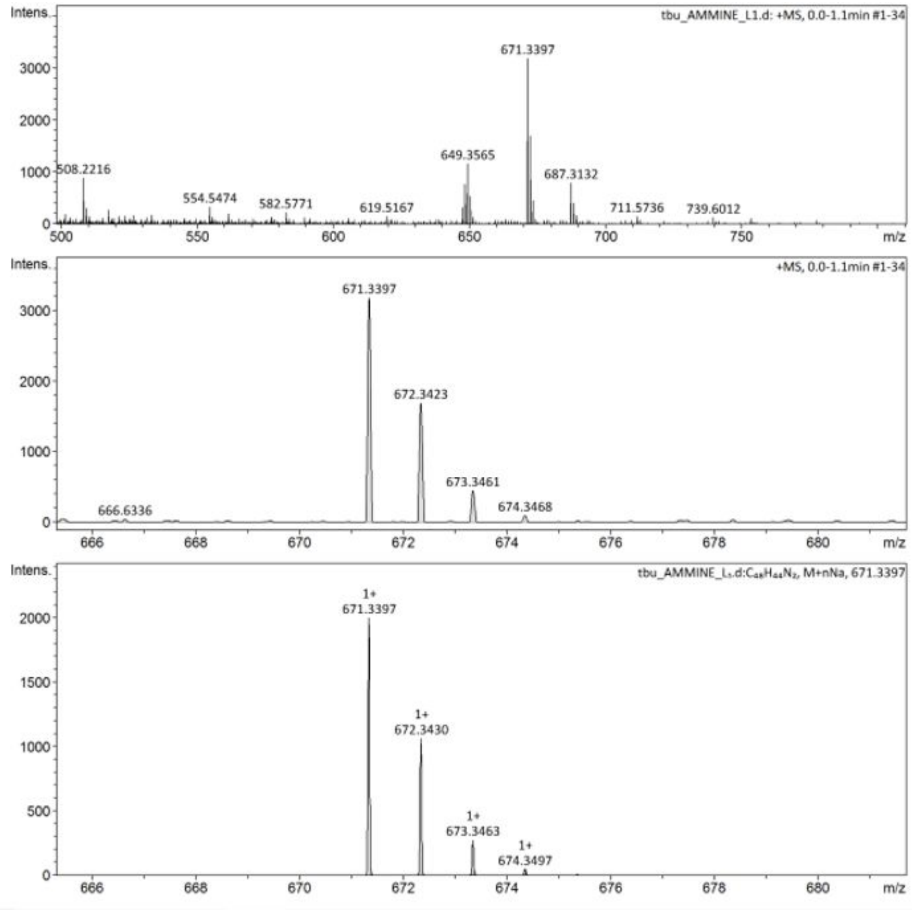

Figure S4: ESI-HRMS of **2**, top) experimental spectra, middle) zoom and bottom) simulated spectra.

3.2 Characterization of 3

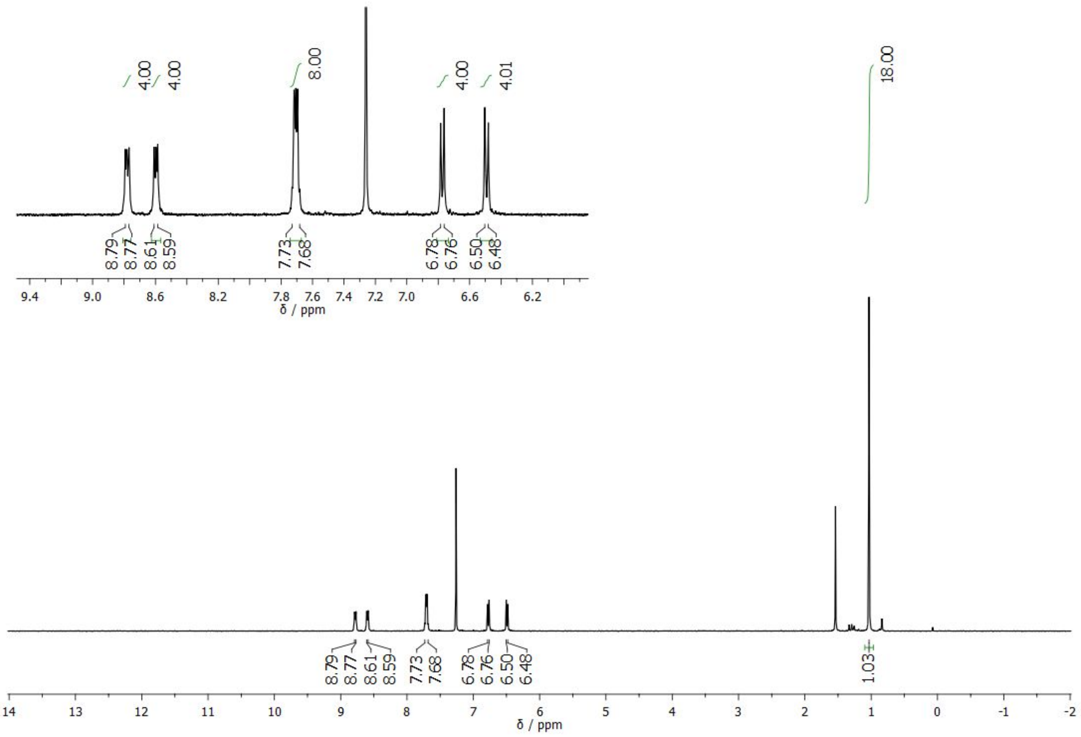

Figure S5: ^{1}H NMR 400 MHz of **3** in $CDCl_3$.

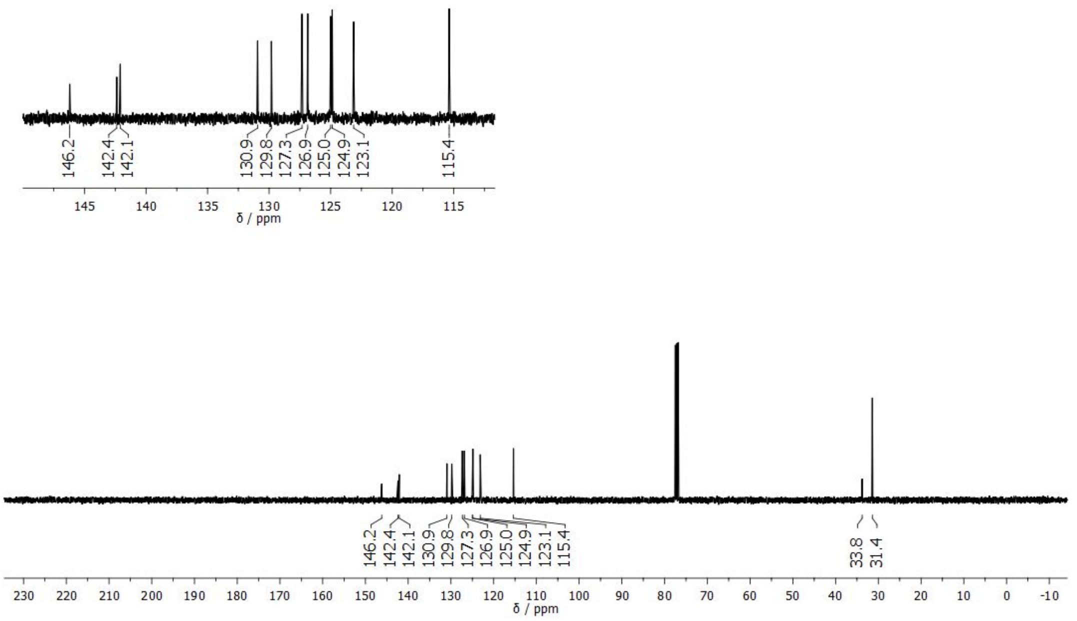

Figure S6: ^{13}C NMR 101 MHz of **3** in $CDCl_3$.

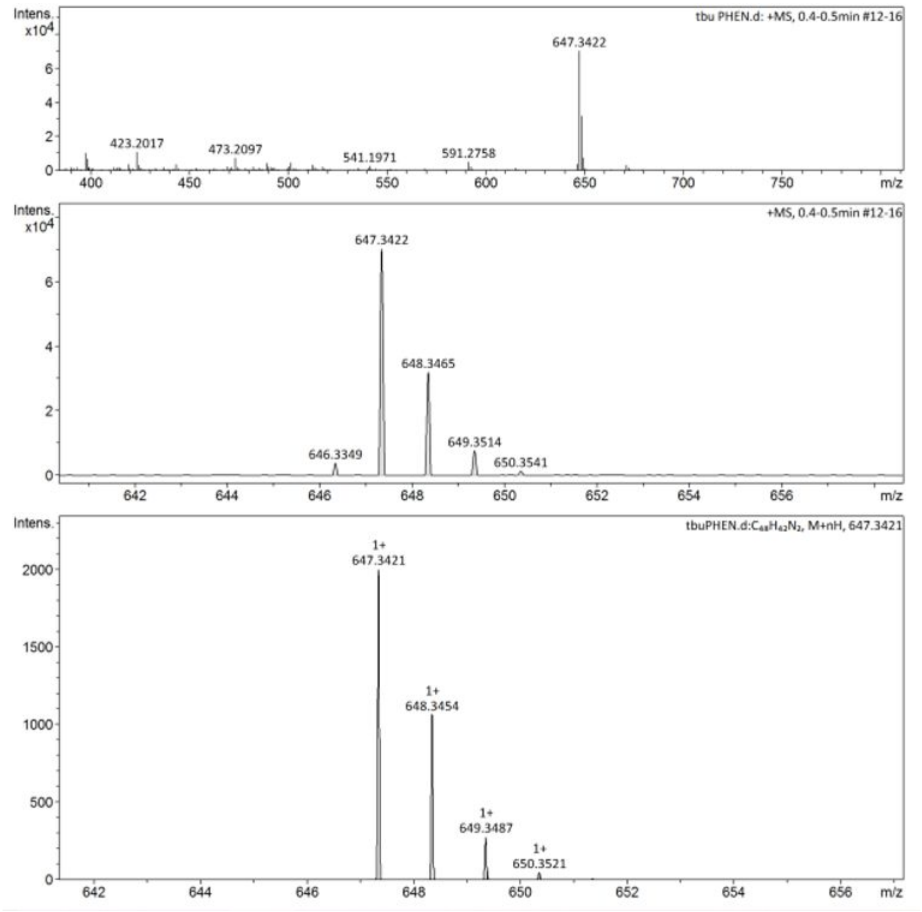

Figure S7: ESI-HRMS of **3**, top) experimental spectra, middle) zoom and bottom) simulated spectra.

3.3 Characterization of 4

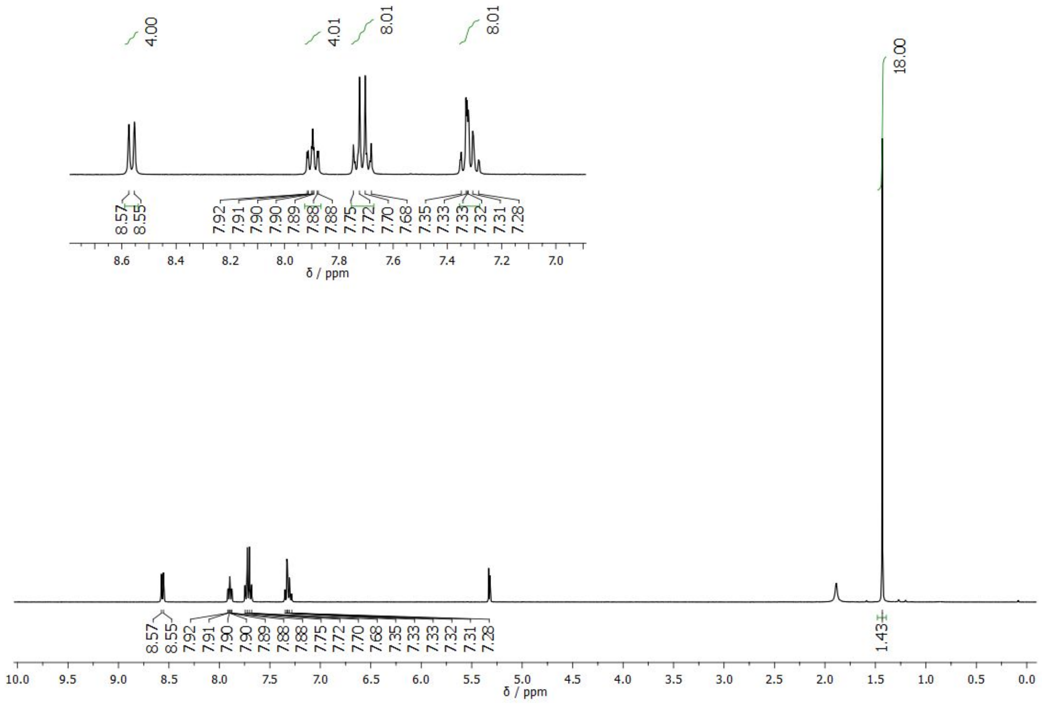

Figure S8: ^{1}H NMR 400 MHz of **4** in CD_2Cl_2.

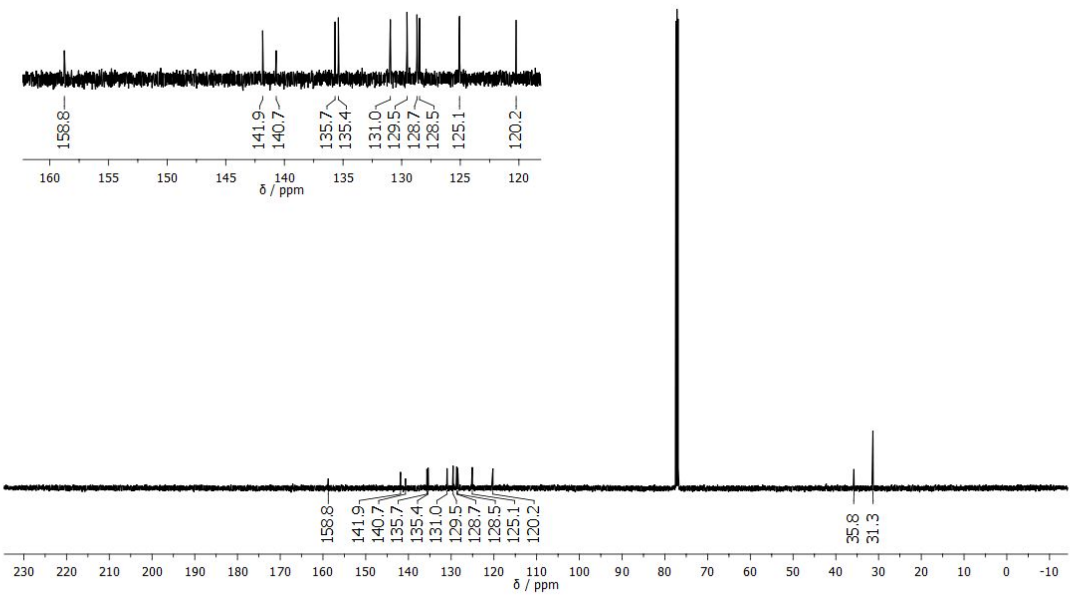

Figure S9: ^{13}C NMR 101 MHz of **4** in $CDCl_3$.

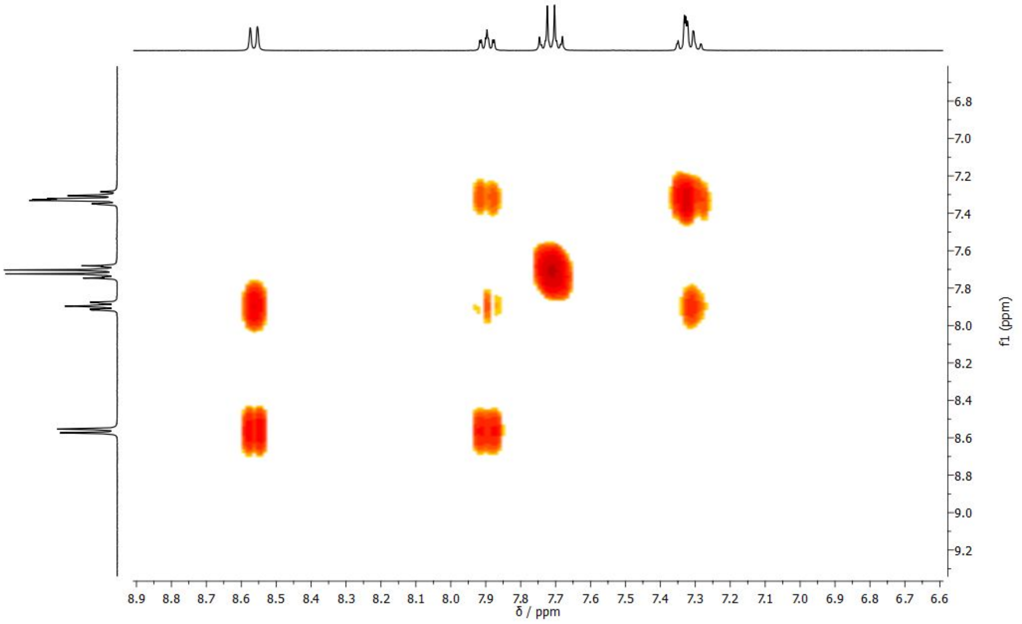

Figure S10: ^{1}H-^{1}H 500 MHz COSY of **4** (aromatic part) in CD_2Cl_2.

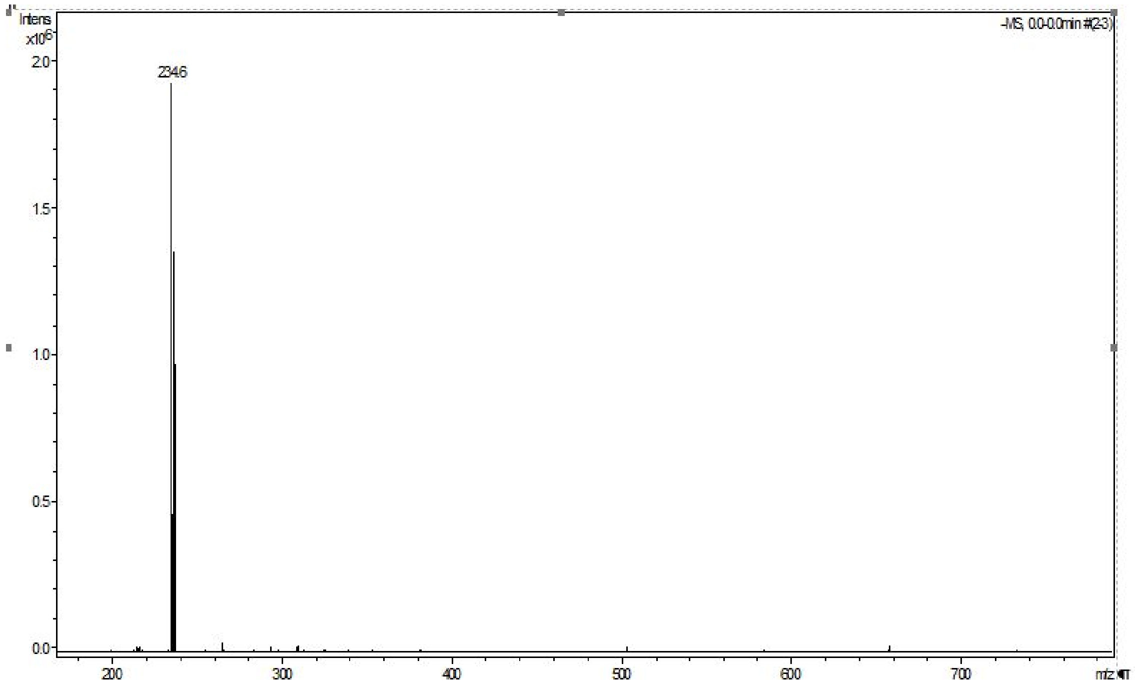

Figure S11: ESI-LRMS of **4**, negative mode.

3.4 Characterization of 5

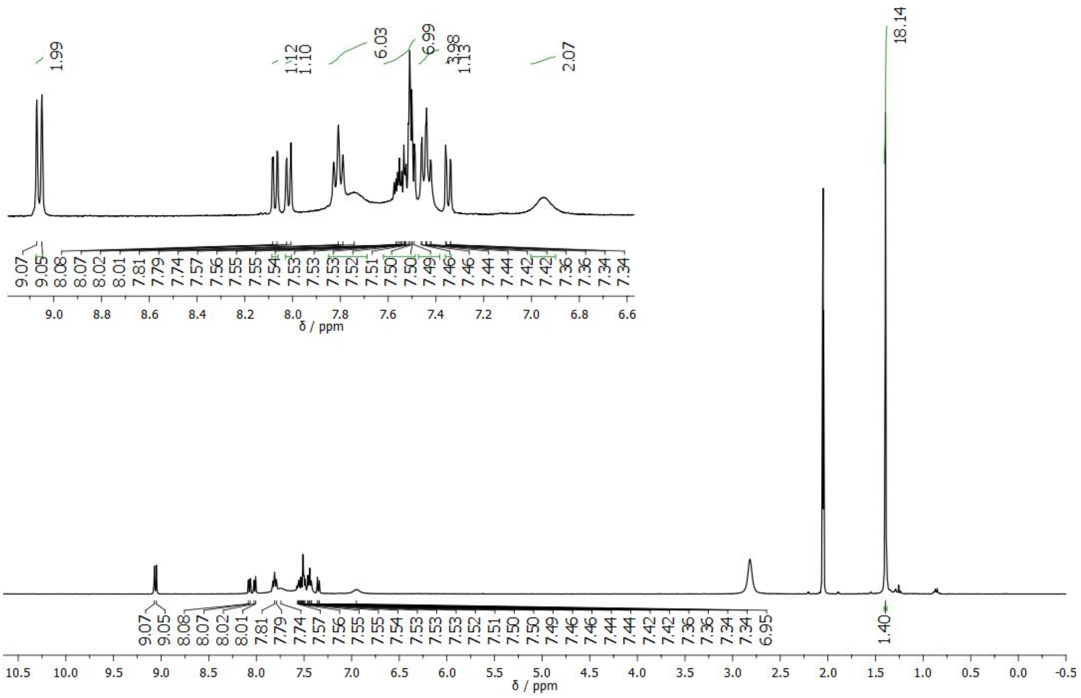

Figure S12: ^{1}H NMR 400 MHz of **5** in Acetone-*d6*.

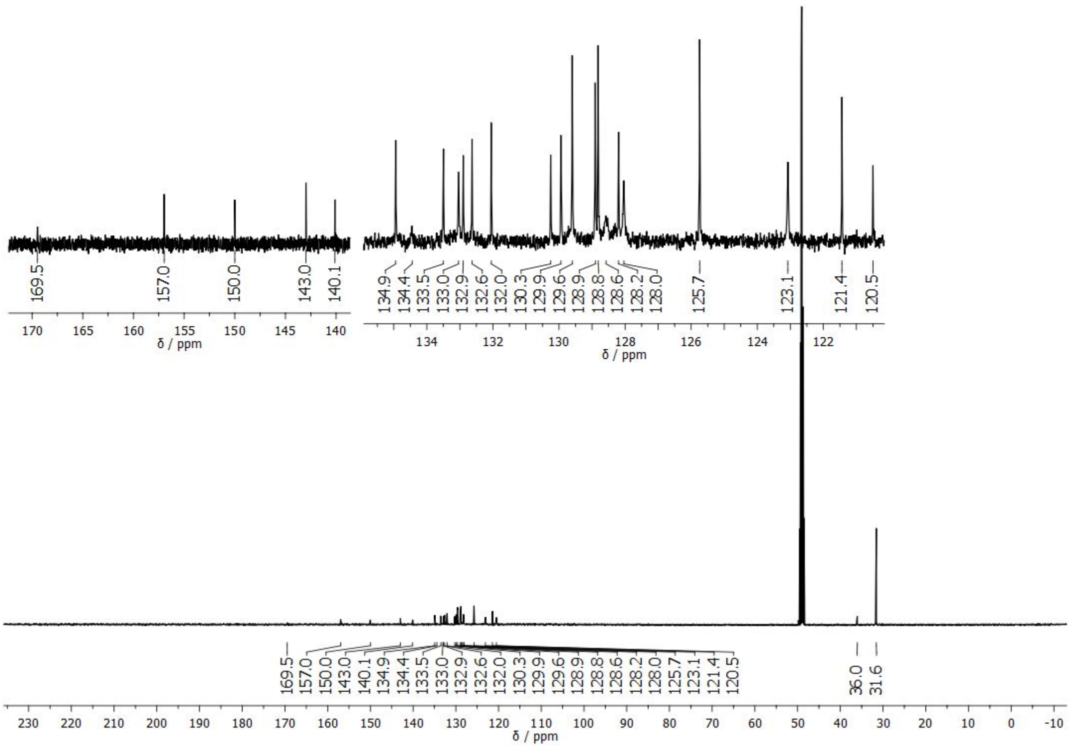

Figure S13: ^{13}C NMR 126 MHz of **5** in MeOD.

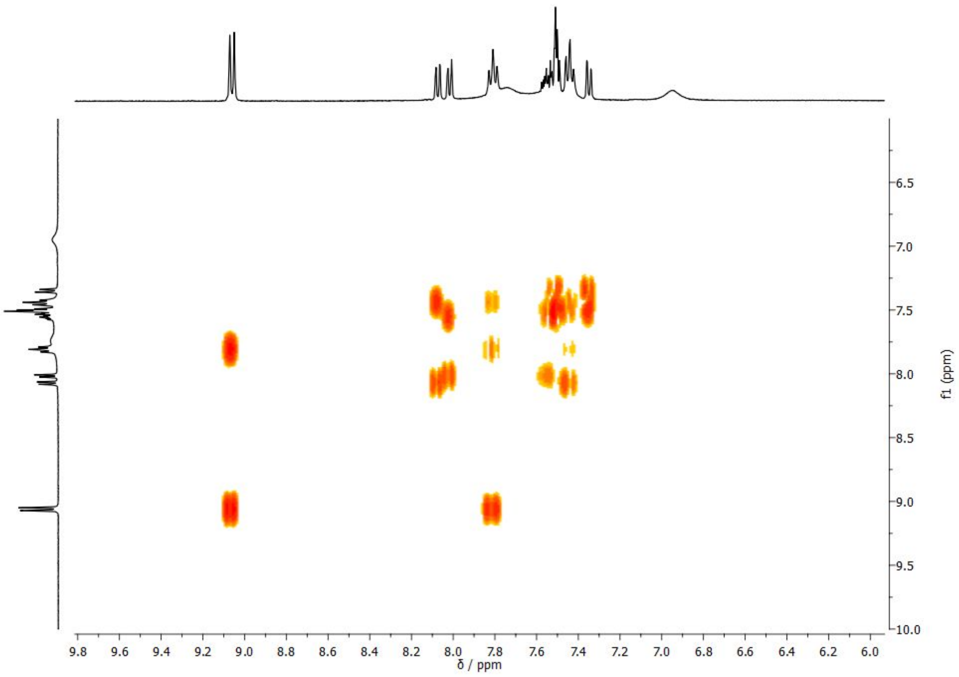

Figure S14: ^{1}H-^{1}H 500 MHz COSY of **5** (aromatic part) in Acetone-*d6*.

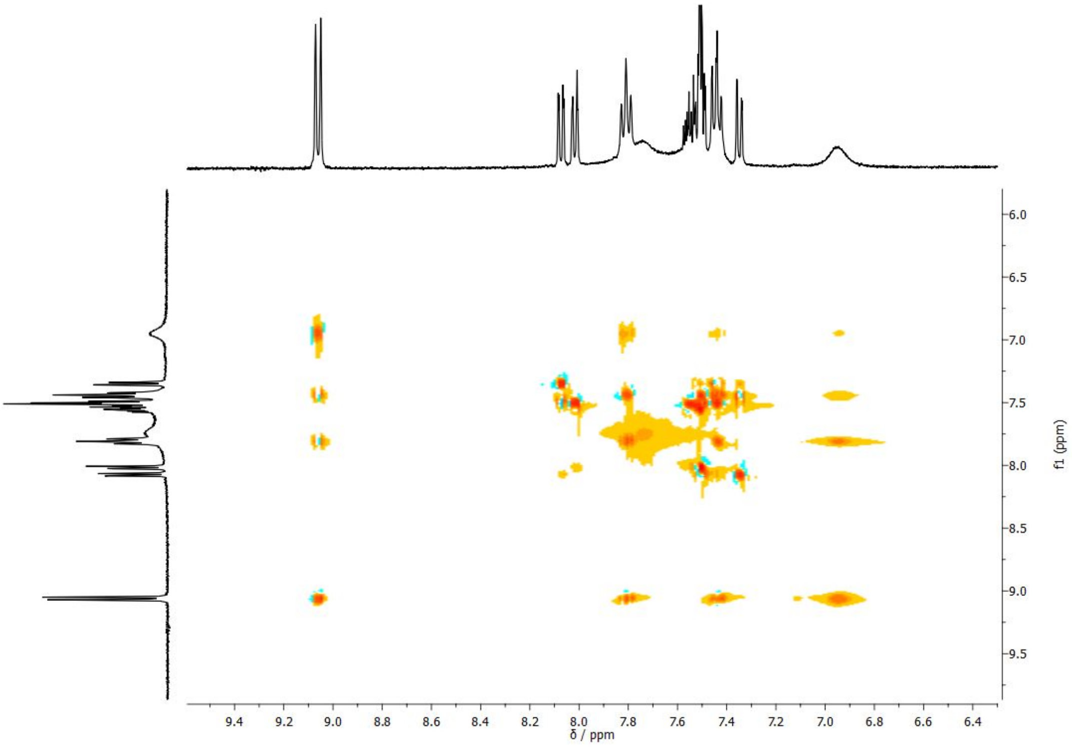

Figure S15: ^{1}H-^{1}H 400 MHz TOCSY of **5** (aromatic part) in Acetone-*d6*.

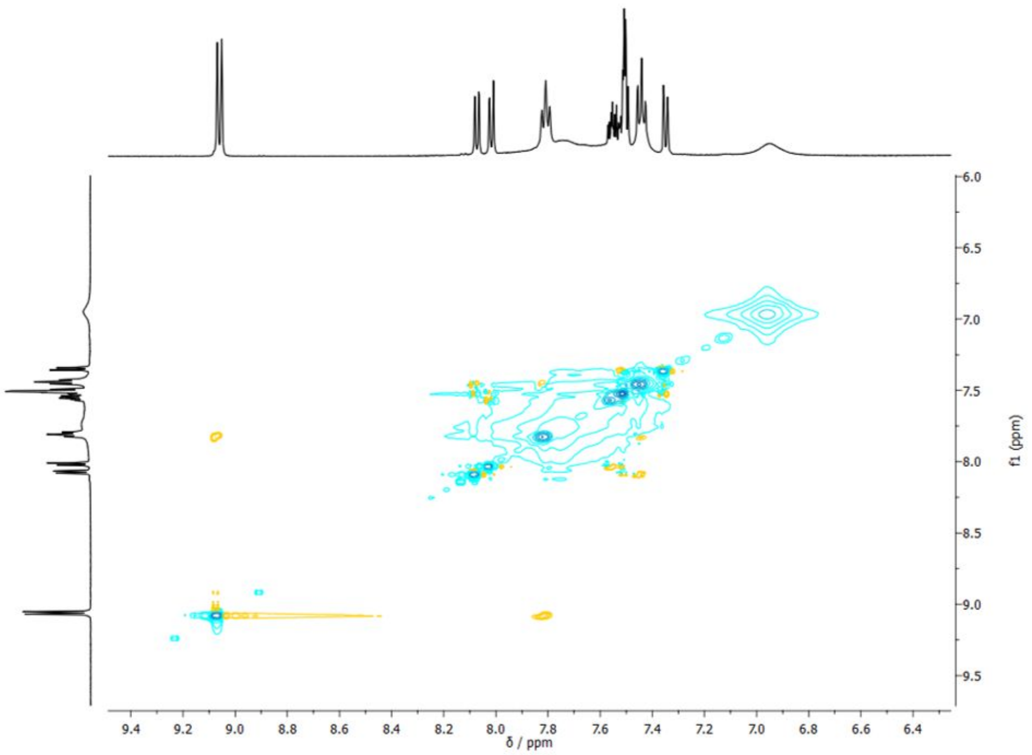

Figure S16: Top ^{1}H-^{1}H 500 MHz NOESY of **5** (aromatic part) in Acetone-*d6.*

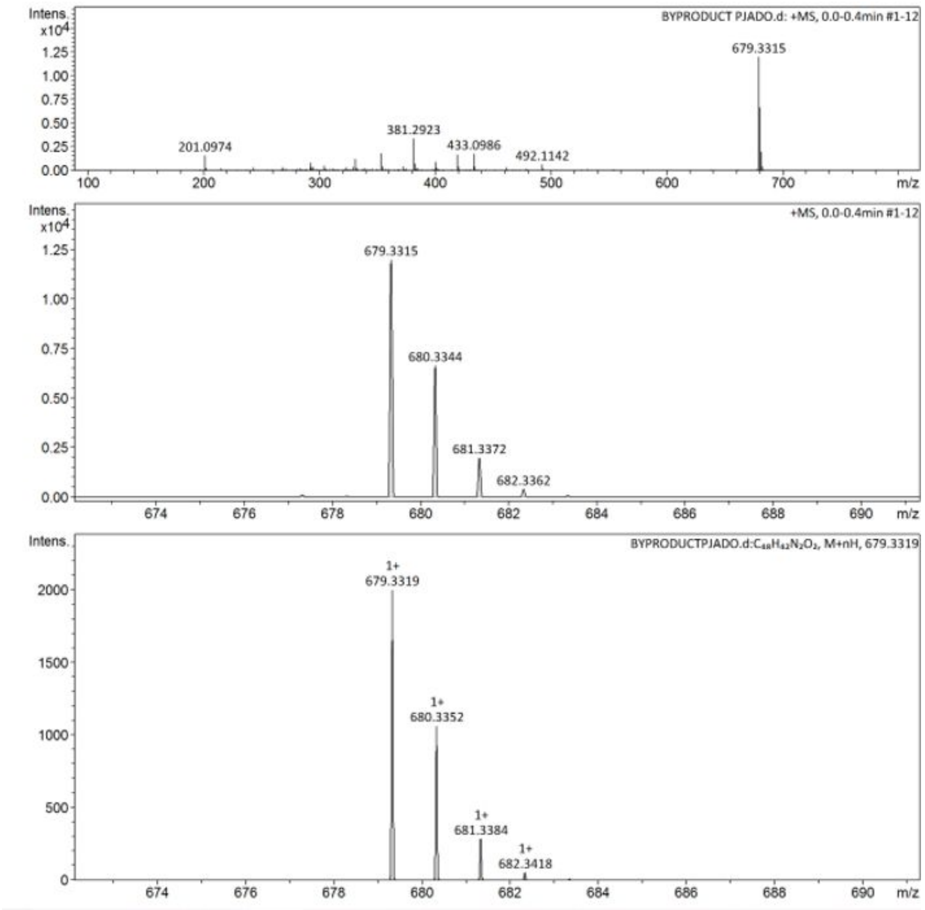

Figure S17: ESI-HRMS of **5**, top) experimental spectra, middle) zoom and bottom) simulated spectra.

3.5 Characterization of 6

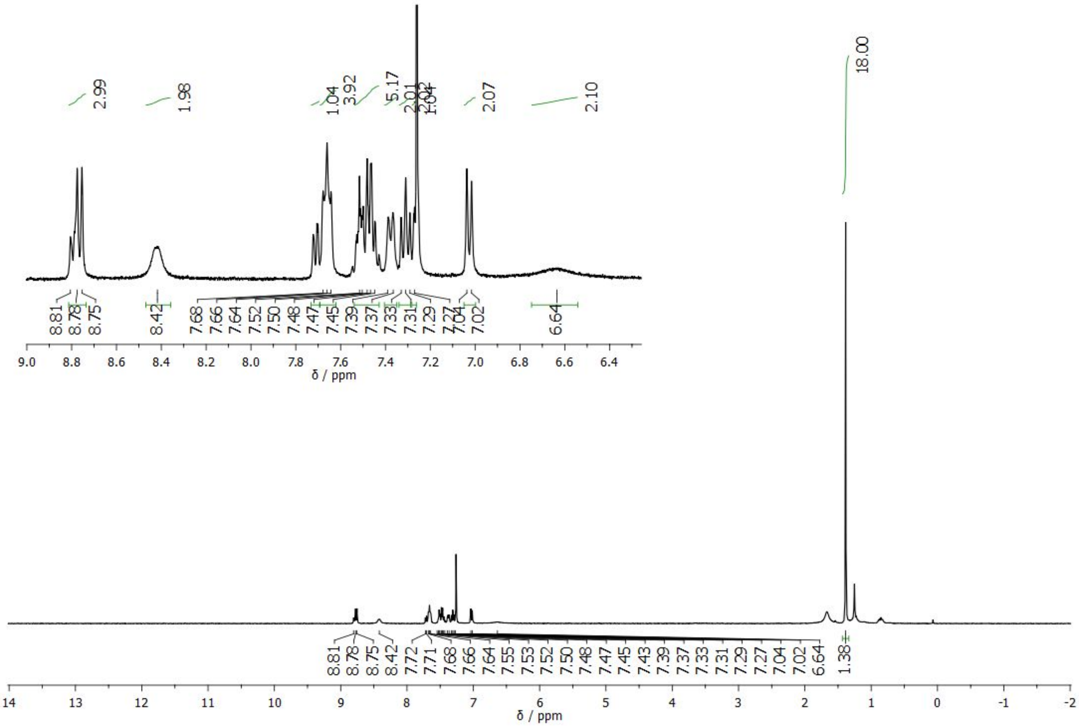

Figure S18: ^{1}H NMR 400 MHz of **6** in $CDCl_3$.

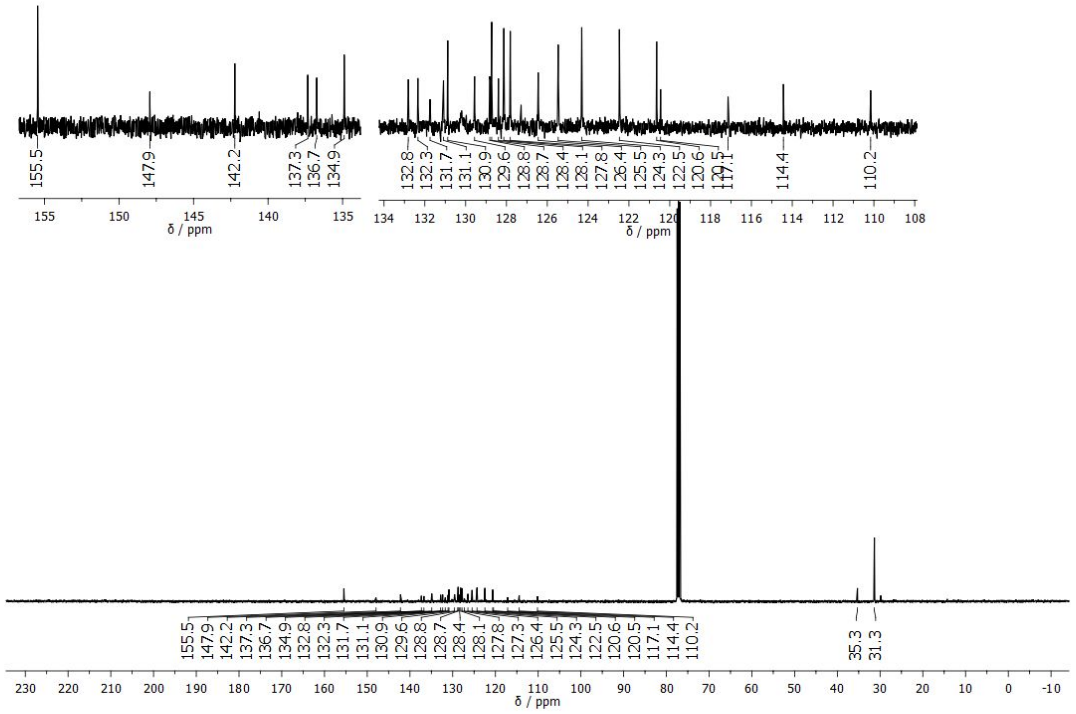

Figure S19: ^{13}C NMR 101 MHz of **6** in $CDCl_3$.

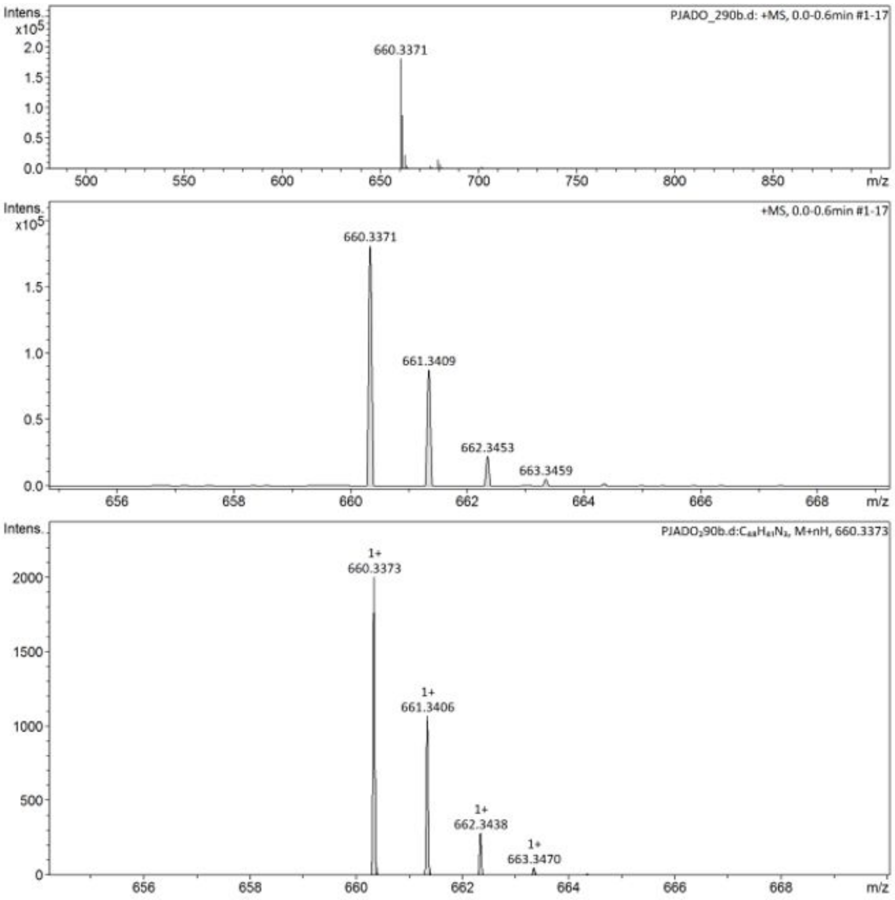

Figure S20: ESI-HRMS of **6**, top) experimental spectra, middle) zoom and bottom) simulated spectra.

4 UV-Vis stability studies

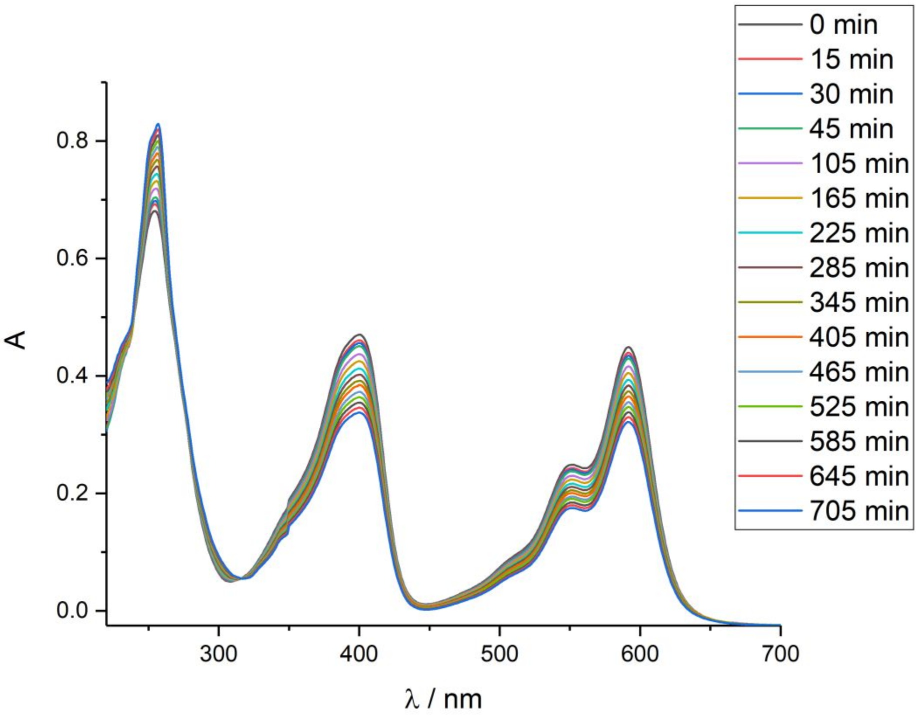

Figure S21: Aerated $CHCl_3$ used, time dependent spectra performed during 12 h on a 2 × 10^{-5} M solution of **4**.

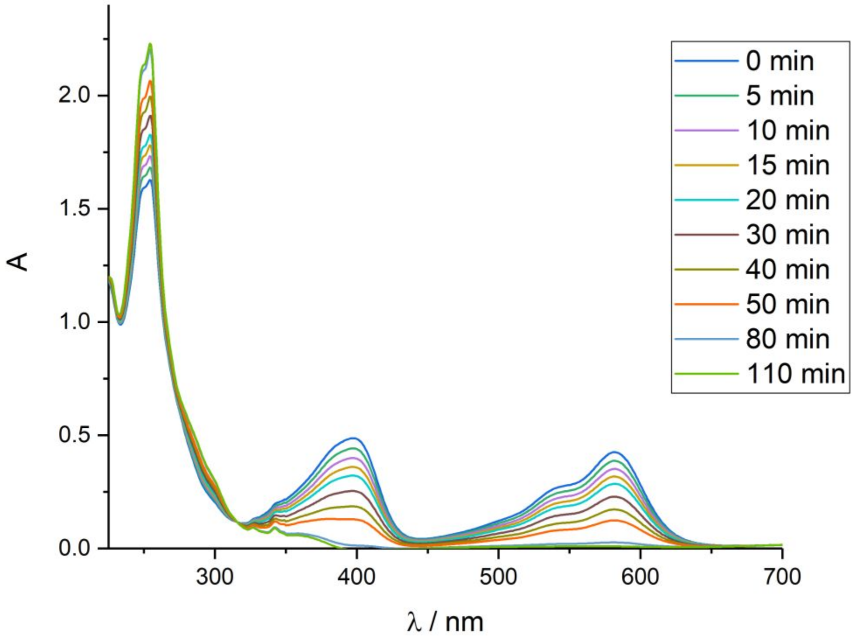

Figure S22: Dry aerated CH_3CN used, time dependant spectra performed on 2 h on a 2 × 10^{-5} M solution of **4**.

5 $H_2{}^{18}O$ Degradation experiment

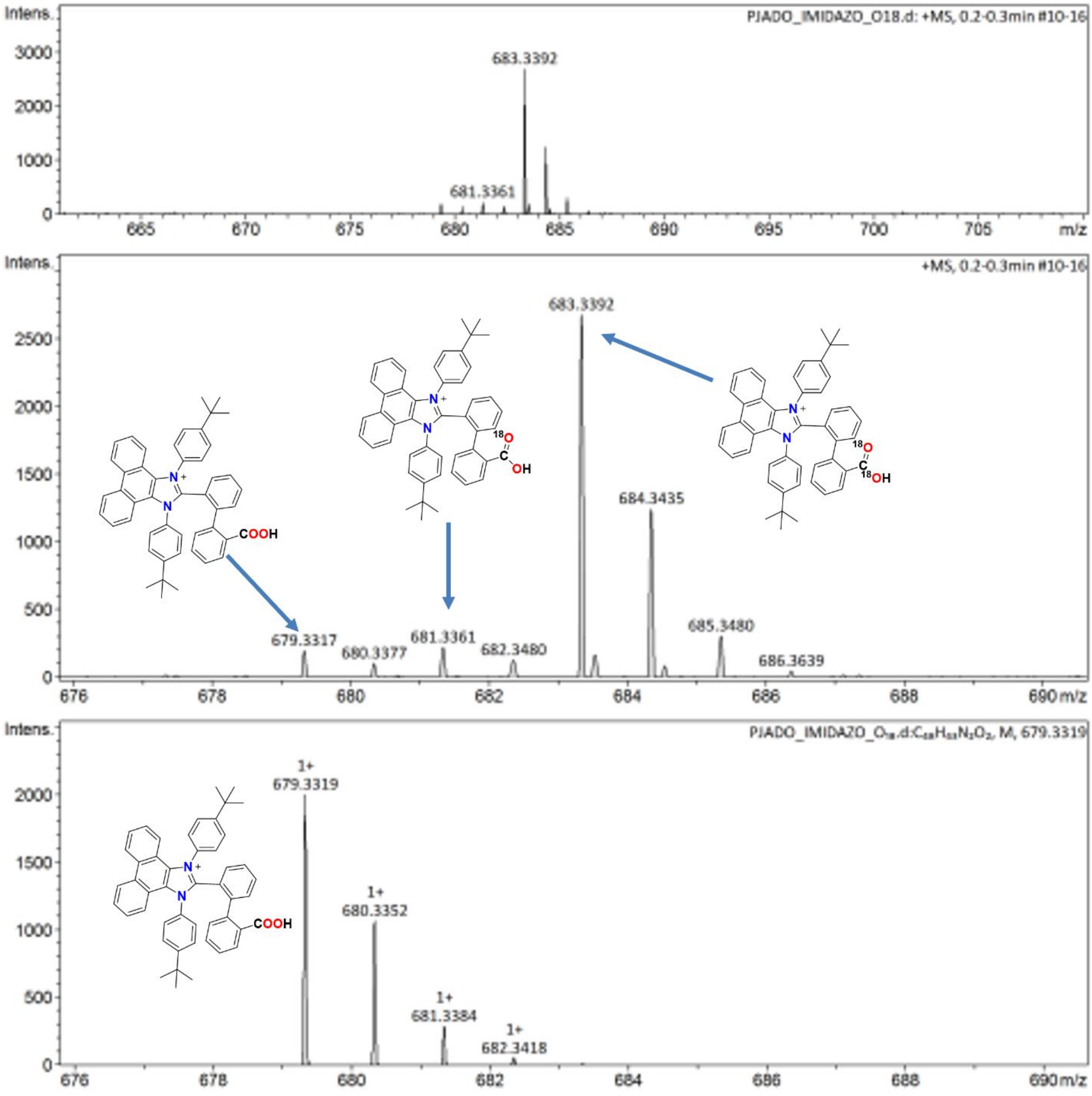

Figure S23: ESI-MS of ^{18}O labelled **5**, from top to bottom: experimental spectra, zoom and simulated spectra for non-marked **5**.

6 NMR degradation experiment

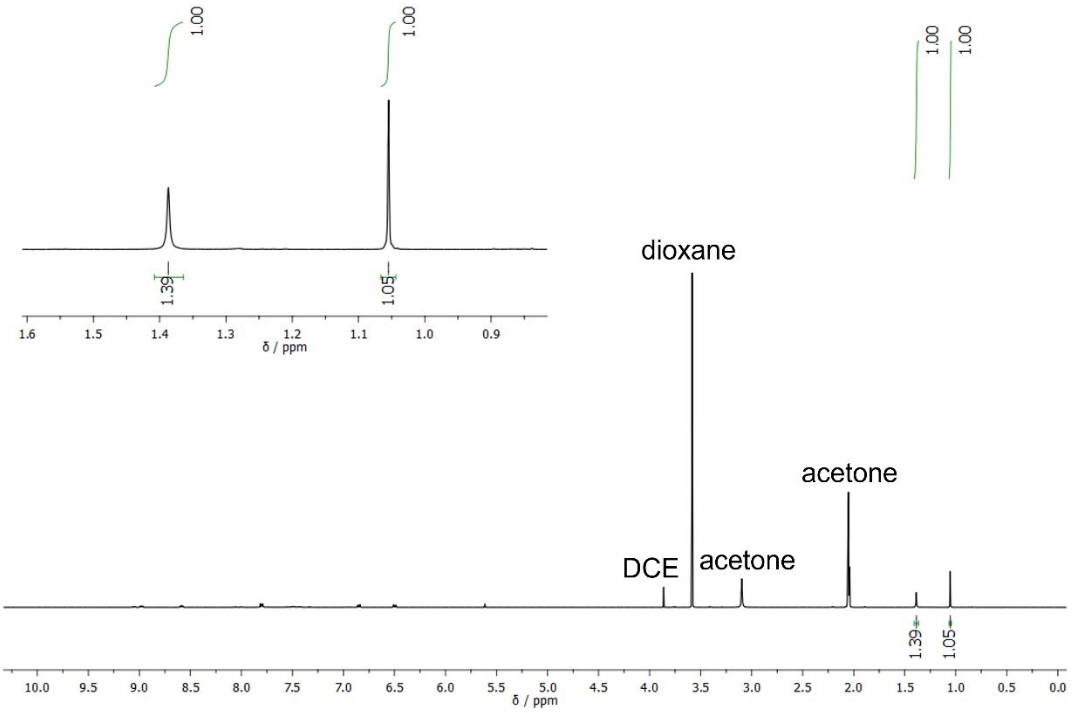

Figure S24: ^{1}H 400 MHz of **4** in Acetone-*d6* following addition of 10 µl D_2O and 1 µl Dioxane (internal standard).

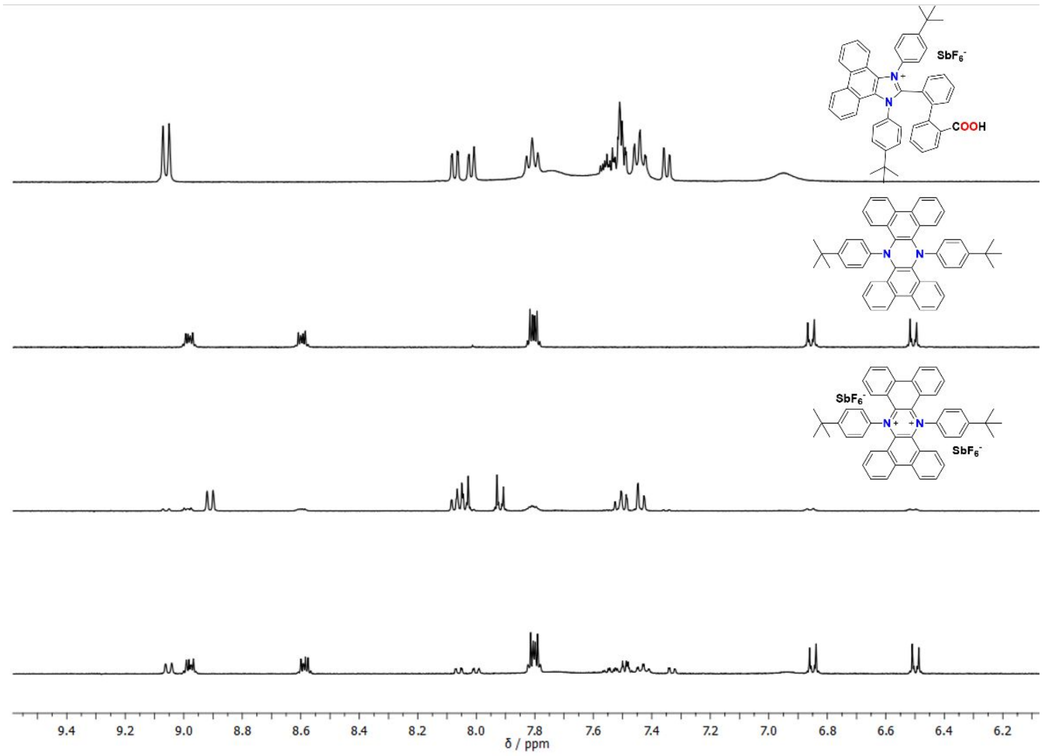

Figure S25: From top to bottom: ^{1}H 400 MHz in Acetone-*d6* (aromatic region) of **5**, **3**, **4** and degradation experiment with 10 µl D_2O (some degradation is already visible in **4** due to solvent moisture).

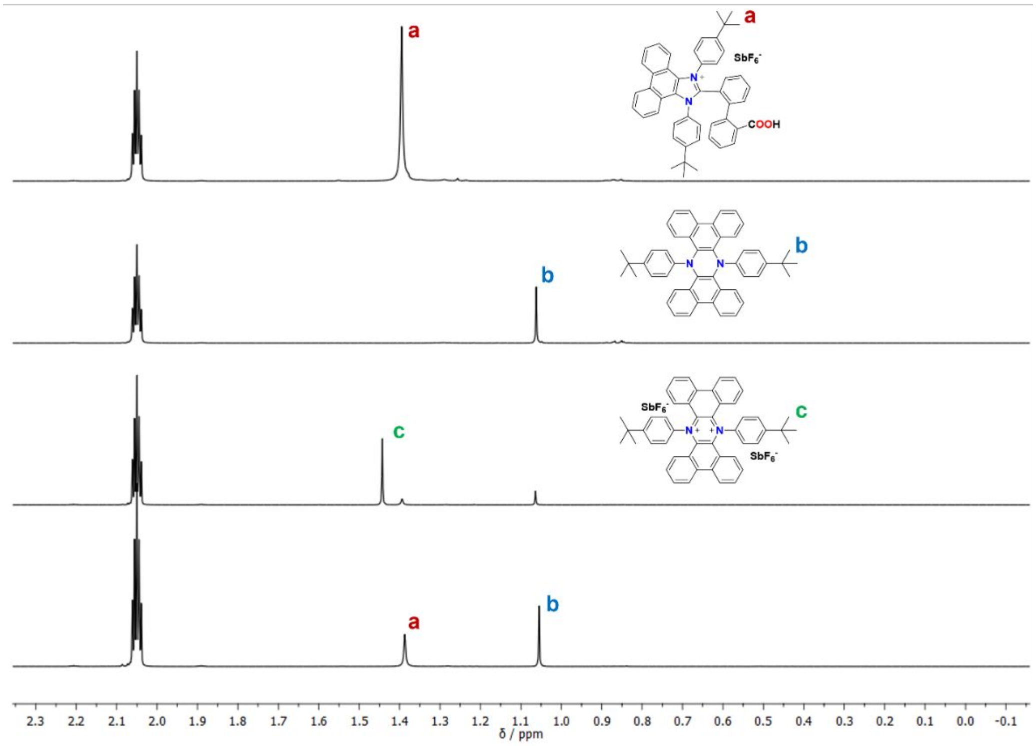

Figure S26: From top to bottom: ^{1}H 400 MHz in Acetone-*d6* (aliphatic region) of **5**, **3**, **4** and degradation experiment with 10 µl D_2O (some degradation is already visible in **4** due to solvent moisture).

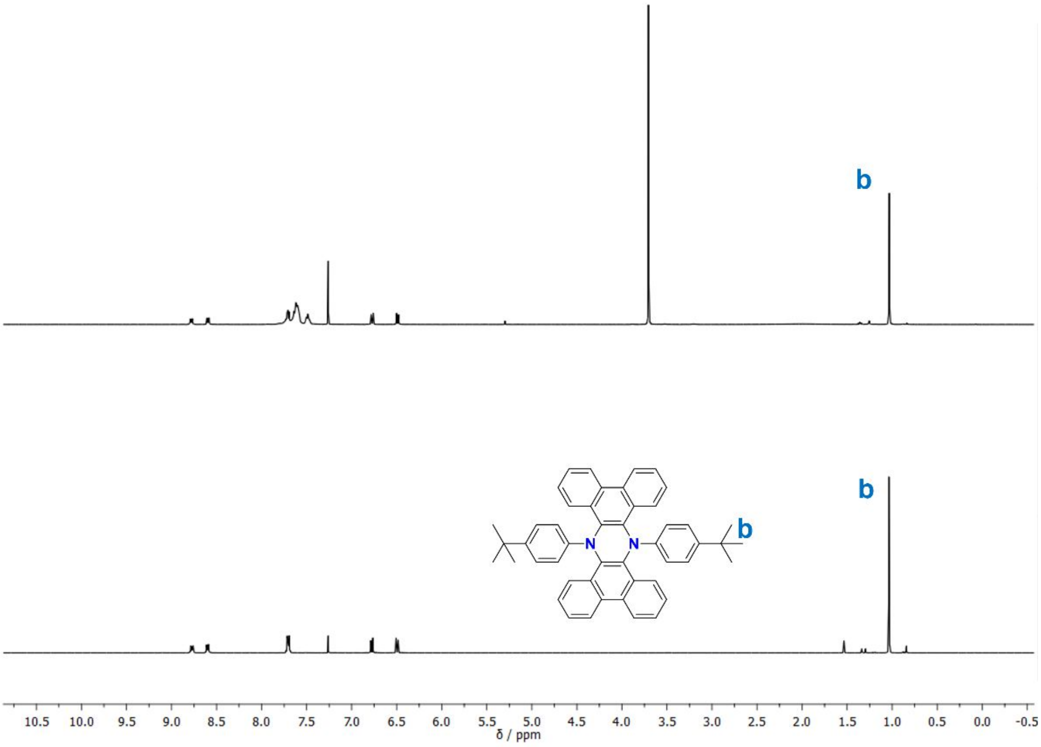

Figure S27: ^{1}H 400 MHz in $CDCl_3$ of: top) reduction experiment with PPh_3 (internal reference signal visible at 3.71 ppm), bottom) pure **4**.

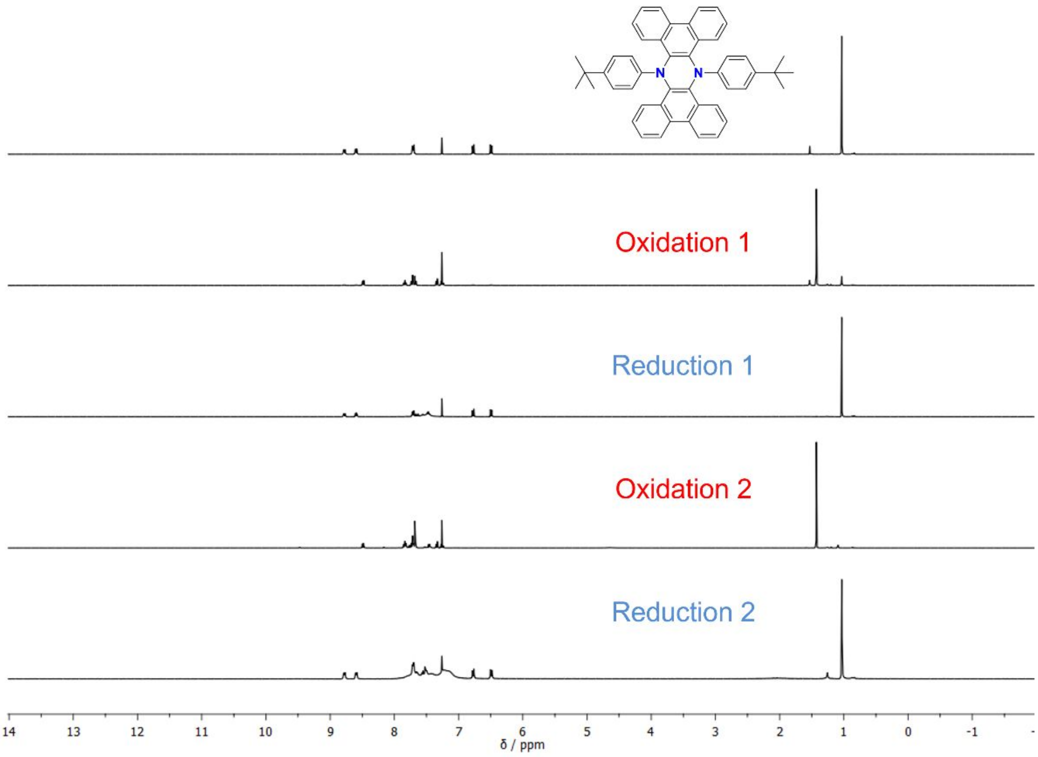

Figure S28: ^{1}H 400 MHz in $CDCl_3$ of oxidation reduction cycles. Each oxidation was carried out by adding 3 eq. $AgSbF_6$, while reduction was performed with 3 eq. PPh_3. top) spectrum of pure **3**.

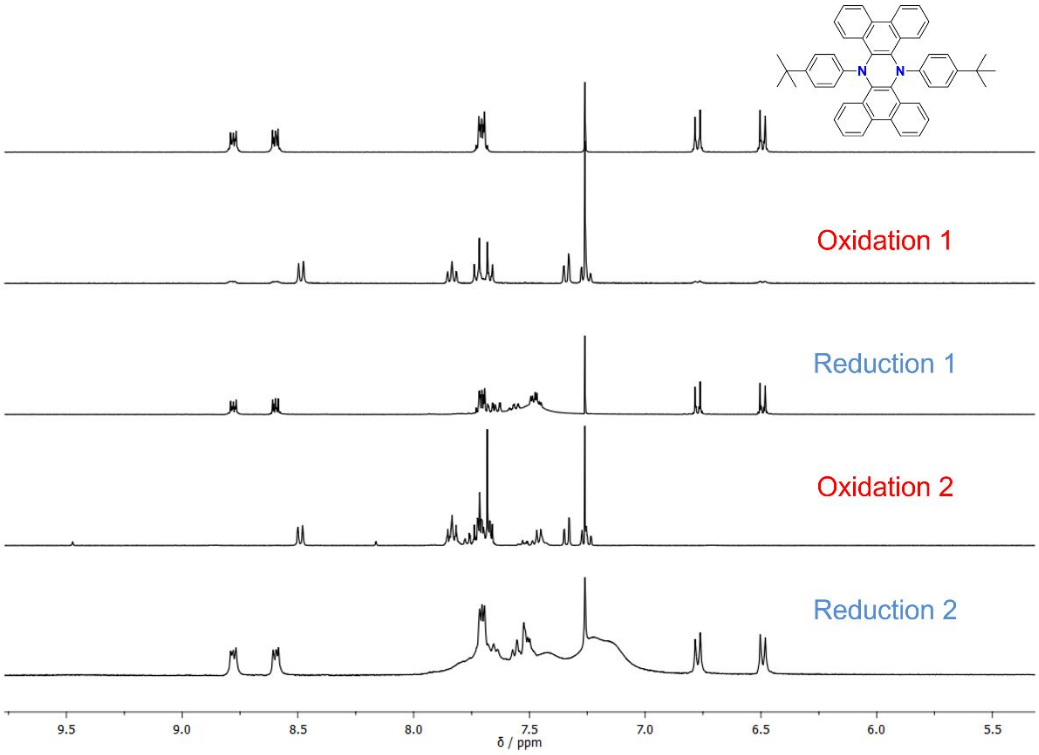

Figure S29: ^{1}H 400 MHz in $CDCl_3$ of oxidation reduction cycles. Zoom on the aromatic part.

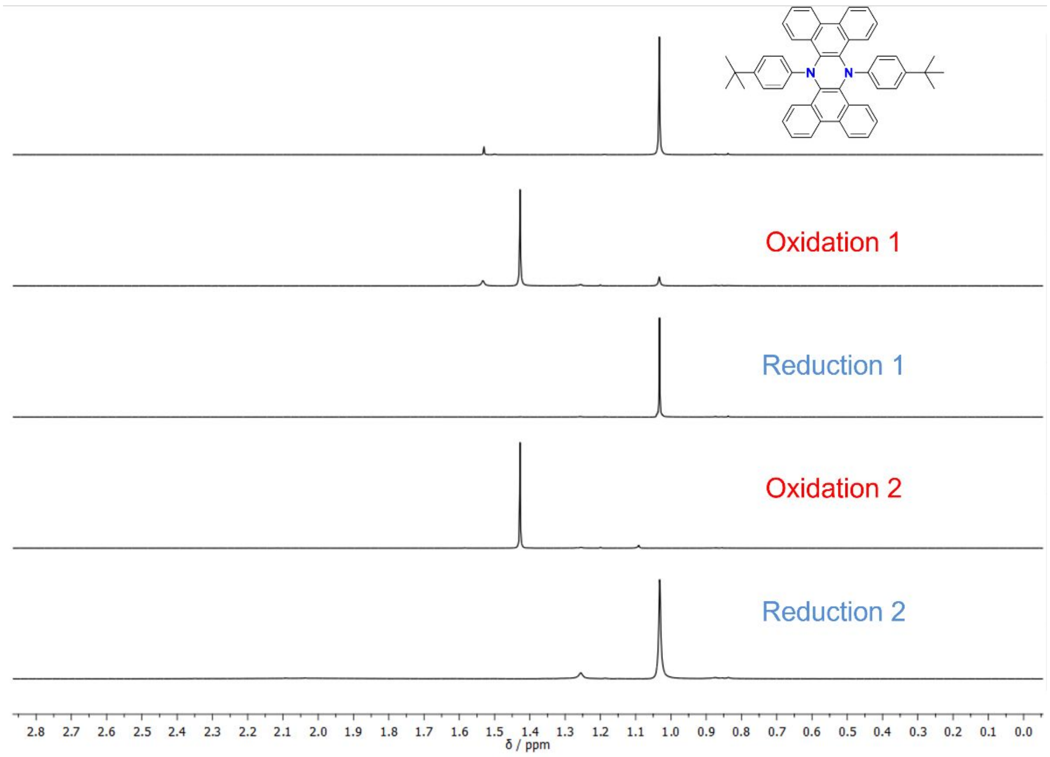

Figure S30: ^{1}H 400 MHz in $CDCl_3$ of oxidation reduction cycles. Zoom on the aliphatic part.

7 Photochemical characterizations

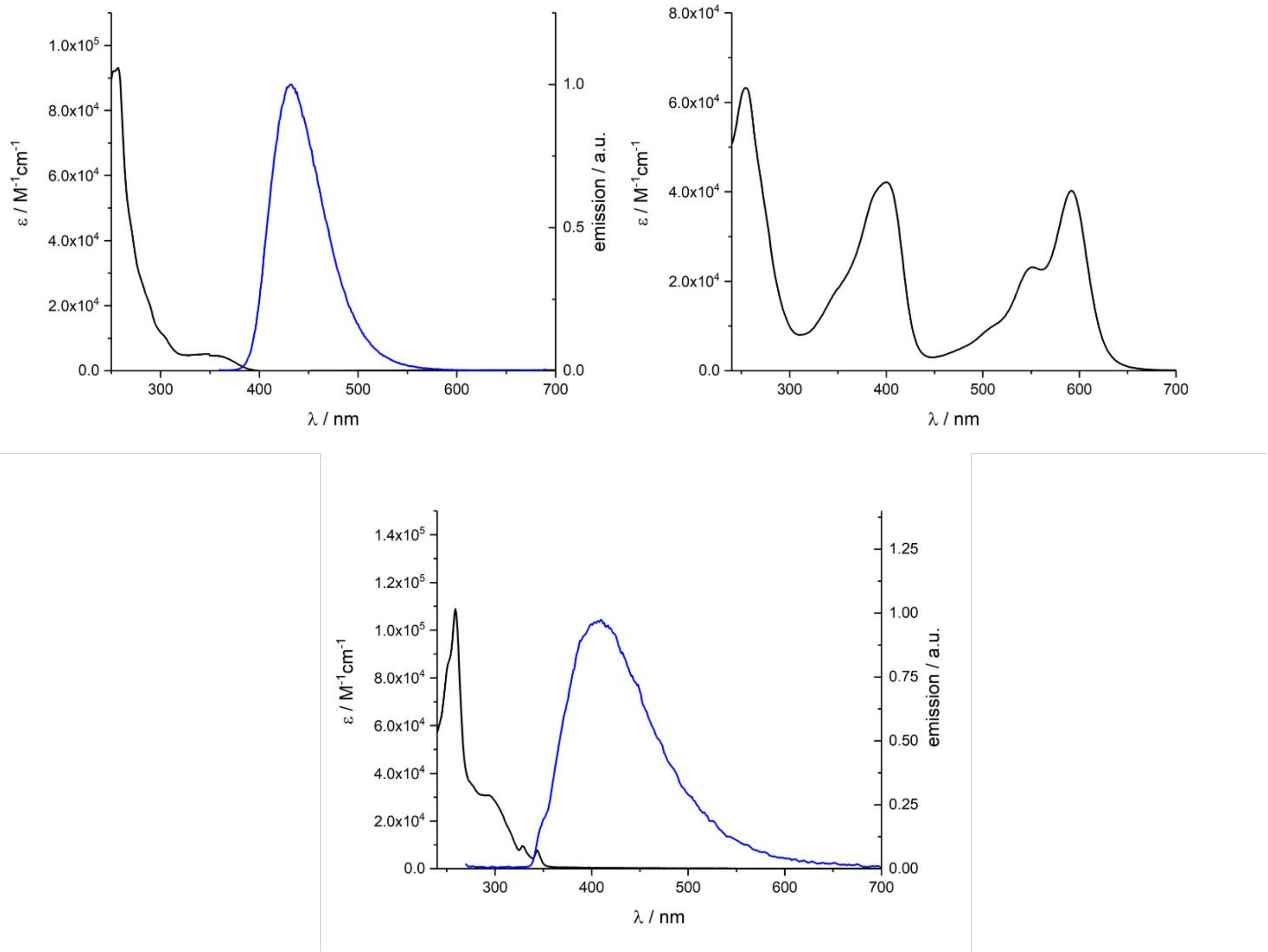

Figure S31: a) top-left, absorption (black) and fluorescence spectrum (blue) of **3** in air equilibrated $CHCl_3$ at room temperature. λ_{ex}=350 nm. b) top-right, absorption (black) of **4** in air equilibrated $CHCl_3$ at room temperature. c) bottom, absorption (black) and fluorescence spectrum (blue) of **5** in air equilibrated $CHCl_3$ at room temperature λ_{ex}= 300 nm.

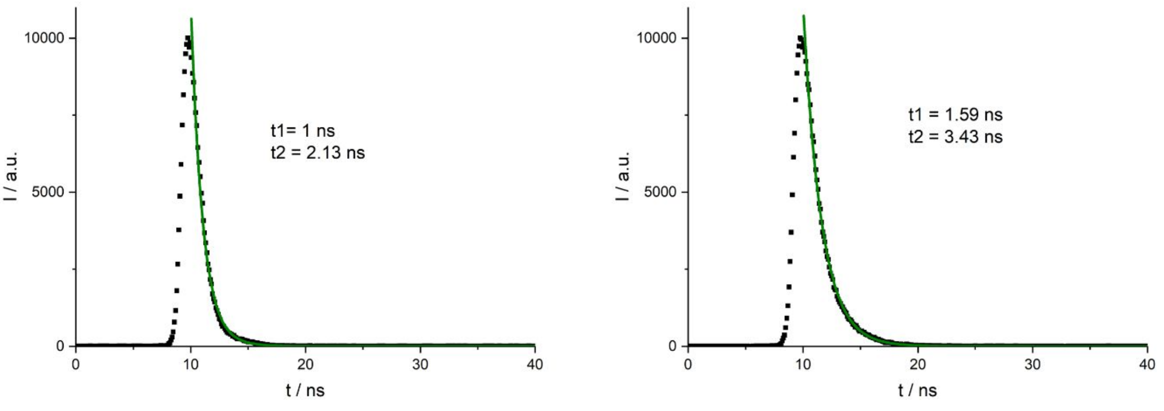

Figure S32: a) left, fluorescence emission decay of **3** in air equilibrated $CHCl_3$ at room temperature. λ_{ex}=285 nm. b) fluorescence emission decay of **5** in air equilibrated $CHCl_3$ at room temperature λ_{ex}=285 nm.

Table S1. Photophysical data for compounds **3**, **4**, **5**.

compound	λ_{max} (nm)	ε /10^4 $M^{-1}cm^{-1}$	λ_{em} (nm)	Φ_{fl}	τ_{fl} (ns)
**3**	257, 351	9.3, 0.46	432	3%	1.00, 2.13
**4**	254, 400, 592	5.9, 4.2, 4.0	n.d.	n.d.	n.d.
**5**	259, 293, 344	10.9, 3.0, 0.67	408	27%	1.59, 3.43

8 Electrochemical characterization

Electrochemical analysis. Cyclic voltammetry experiments were carried out at room temperature in Argon-purged $CHCl_3$ with an Autolab PGSTAT128N potenziostat/galvanostat. The working electrode consisted of a glassy carbon electrode (5 mm diameter), the reference electrode was a standard calomel electrode (SCE) and the auxiliary electrode was Pt. Working electrode was polished on a felt pad with 0.05 or 0.3 µm alumina suspension and sonicated in deionized water and ethanol for 1 minute before each experiment. Tetrabutylammonium hexafluorophosphate ($TBAPF_6$) is added to the solution as a supporting electrode at concentrations typically 100 times higher than the electroactive analyte.

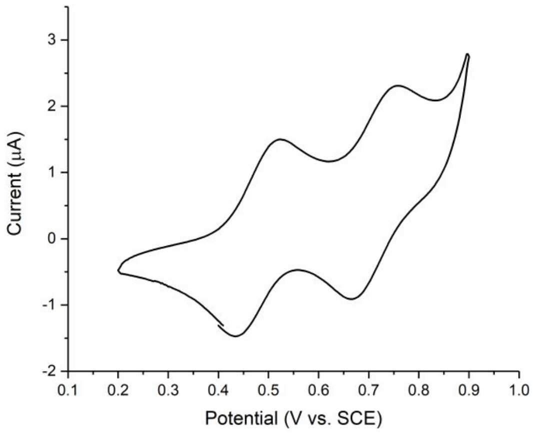

Fig. S33: a) Cyclic voltammograms of **3** in $CHCl_3$ (ca. 1 mM). Scan rates: 0.025 V s^{-1} (black line), $TBAPF_6$ (0.1 M) is used as a supporting electrolyte; ferrocene (Fc) CV was recorded in the same conditions in a separate experiment (incompatible as internal reference) and used as reference ($E_{Fc+/Fc}$ = 0.44 V vs. SCE).

9 Calculations

Methods

All the DFT calculations were performed with the B3LYP hybrid functional[1–4] as implemented in the Gaussian 16 suite of programs.[5] Dispersion corrections were taken into account by including the Grimme's D3 function including a Becke-Johnson damping.[6–8] NBO calculations were carried out using Natural Atomic Orbital program[9] as implemented in Gaussian. NICS calculations were performed using the Gauge-Independent Atomic Orbital method (GIAO).[10] Solvent effects were estimated by means of the Polarizable Continuum Model (PCM).[11,12]

Results

In order to assess the aromaticity of *N,N*-diarylphenazinium dication **4**, we performed DFT studies at the B3LYP-D3BJ/6-31G* level of theory and analyzed the aromatic character of **4** according to geometric, electronic and magnetic criteria.

The structural results at this computational level show geometric data in good agreement with the X-ray structure (Figure S34). The four C-N bonds are similar to each other and both C-C bonds resulted to be identical. The calculated minimum energy conformation of **4** corresponds to an almost planar half-chair structure, with a N-C-C-N dihedral angle of ca. 31 deg. (absolute value), also in nice agreement with the experimentally found structure. Another boat conformation was found (Figure S34), with a N-N-C-N dihedral angle of ca. 14 deg., thus showing an almost eclipsed interaction between both *p*-*tert*-butyl-phenyl groups. This latter structure was calculated to be ca. 17 kcal/mol more energetic than its half-chair congener. Therefore, the following discussion will be restricted to the minimum energy structure gathered in Figure S34.

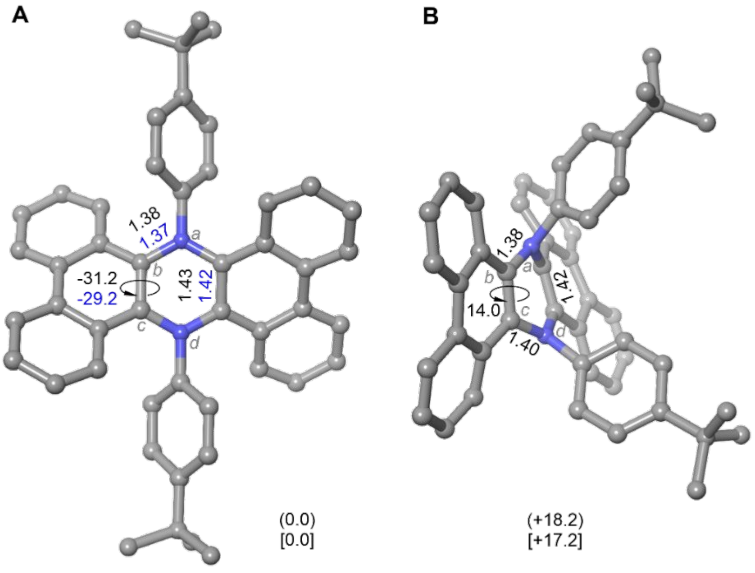

Figure S34. Fully optimized structures (B3LYP-D3BJ/6-31G* level of theory) of half-chair (A) and boat (B) conformations of N,N-diarylphenazinium dication **4**. Numbers in blue correspond to X-Ray data. Bond distances and

angles are given in Å and deg., respectively. Numbers in parentheses and square brackets correspond to the relative internal and Gibbs (at 298.15 K) energies, respectively, and are given in kcal/mol. Hydrogen atoms have been omitted for clarity. Nitrogen and carbon atoms are represented in blue and gray, respectively.

The possible aromatic character of **4** was analysed considering the Wiberg bond orders[13] in the Natural Atomic Orbital (NBO) basis[14] along the central pyrazine-1,4-diium dication moiety. According to our results, the computed C-C and C-N bond orders are quite close to each other and show lower values than those found for the parent pyrazine-1,4-diium dication. These results indicate a lower π–overlap in **4**, generated by the departure from planarity imposed by the densely substituted environment. The aromaticity of the dication was quantified by means of the Bird equation[15] adapted to Wiberg-NBO bond orders and to six-membered unsaturated heterocycles:[16]

$$I_6 = 100\left[1 - \frac{100}{33.3\overline{N}}\sqrt{\frac{4\left(N_{CN}-\overline{N}\right)^2 + 2\left(N_{CC}-\overline{N}\right)^2}{6}}\right]. \qquad (1.1)$$

In eq. (1.1) I_6 is the Bird aromaticity index for a six-membered unsaturated ring, N_{CN} and N_{CC} stand for the C-N and C-C bond orders, respectively, and $\overline{N}$ corresponds to the average bond order value of the pyrazine-1,4-diium dication moiety of **4**:

$$\overline{N} = \frac{4N_{CN} + 2N_{CC}}{6}. \qquad (1.2)$$

DFT-NBO calculations yield a value of $I_6 = 96$ (for D_{6h}-symmetric benzene, $I_6 = 100$), which points to a highly aromatic character for **4** on the basis of bond order equalization.

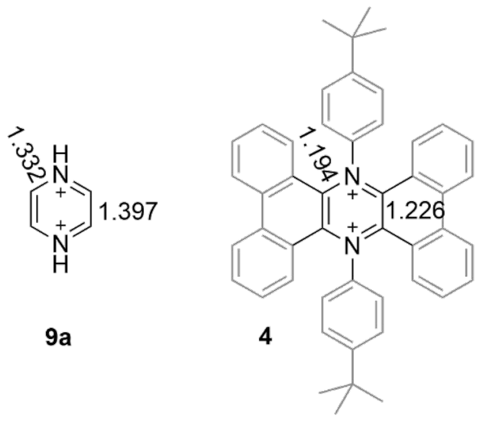

Figure S35. Wiberg bond orders (in atomic units) in the Natural Atomic Orbital basis of pyrazine-1,4-diium dication **9a** (left) and *N,N*-diarylphenazinium dication **4** (right).

In order to assess the aromatic character of **4** considering structural criteria[17] based on bond distances, the Harmonic Oscillator Model of Aromaticity[18] (HOMA) descriptor was calculated, according to the following equation:[19]

$$HOMA = 1 - \frac{1}{6}\left[4\alpha_{CN}(R_{CN}^{opt} - R_{CN})^2 + 2\alpha_{CC}(R_{CC}^{opt} - R_{CC})^2\right] \tag{1.3}$$

where α_{CN} and α_{CC} are two parameters associated with the C-N and C-C bonds in the form

$$\alpha_{CN} = \frac{2}{\left(R_{CN}^{C-N} - R_{CN}^{opt}\right)^2 + \left(R_{CN}^{C=N} - R_{CN}^{opt}\right)^2} \tag{1.4}$$

and

$$\alpha_{CC} = \frac{2}{\left(R_{CC}^{C-C} - R_{CC}^{opt}\right)^2 + \left(R_{CC}^{C=C} - R_{CC}^{opt}\right)^2}, \tag{1.5}$$

respectively. In eq. (1.3), R_{CN}^{opt} and R_{CC}^{opt} are the optimal bond distances for C-N and C-C bonds, respectively. In this case, taking $R_{CN}^{opt} = 1.334$ Å and $\alpha_{CN} = 93.95$ Å^{-2} for the four C-N bonds,[19] and $R_{CC}^{opt} = 1.381$ Å and $\alpha_{CC} = 278.0$ Å^{-2} for the two C-C bonds[20] of **4**, a value of HOMA=0.69 is obtained (HOMA=1.00 for benzene). This value is substantially lower than the I_6 value computed according to eq. (1.1) and reflects the departure of the C-N and C-C bond distances in **4** from the optimal values expected for a standard planar aromatic nitrogen-containing heterocycle, rather than a loss of aromaticity. In our opinion, a bond order-based aromaticity index, such as I_6, less dependent on parametric terms, is more appropriate for the quantification of aromaticity in this particular case.

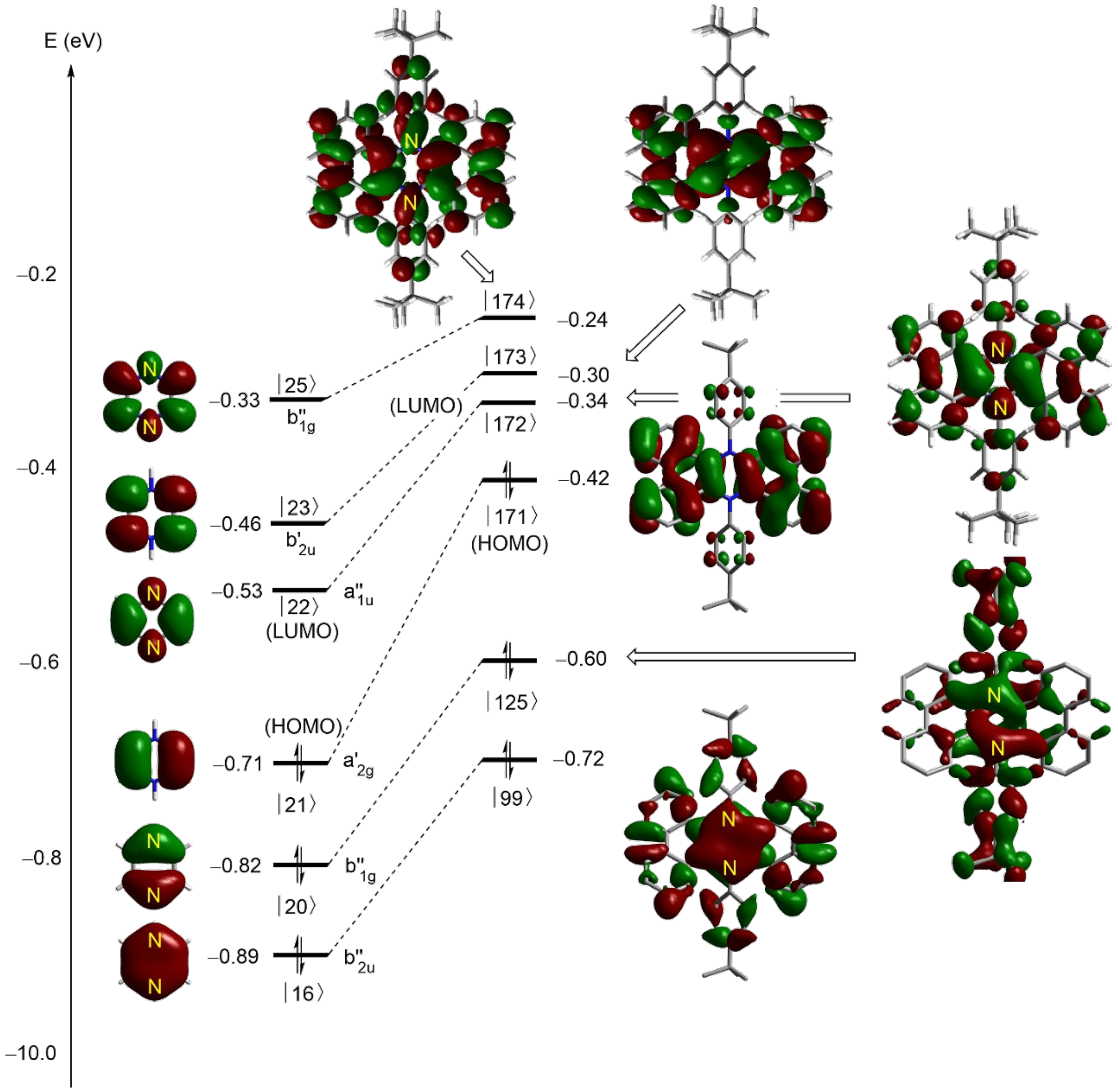

Figure S36. Kohn-Sham molecular orbitals (KS-MO's) of **4**, calculated at the B3LYP-D3BJ/6-31G* level of theory. The energies and shapes of the KS-MO's of D_{2h}-symmetric pyrazine-1,4-diium dication are also shown.

The Kohn-Sham molecular orbitals (KS-MO's) of **4** agree with its aromatic character. Thus, analysis of the occupied and virtual KS-MO's associated with the aromatic D_{2h}-symmetric pyrazine-1,4-diium dication show the six π–orbitals expected for an π–aromatic system, namely the b''_{2u}, b''_{1g} and a'_{2g} occupied MO's, as well as the a''_{1u}, b'_{2u} and b''_{1g} unoccupied MO's shown in Figure S36. These orbitals mix with the phenyl groups around the central *N,N*-diarylphenazinium moiety, giving rise to a complex array of KS-MO's, whose topologies can be assimilated to the previously shown three occupied and three virtual orbitals associated with a six-member π–aromatic ring (Figure S36). It is noteworthy that the π–orbitals of **4** show a slight distortion with respect to the parent pyrazine-1,4-diium dication because of the departure of the central ring from perfect planarity, generated by the steric clash associated with the *ortho* hydrogens of the six phenyl groups.

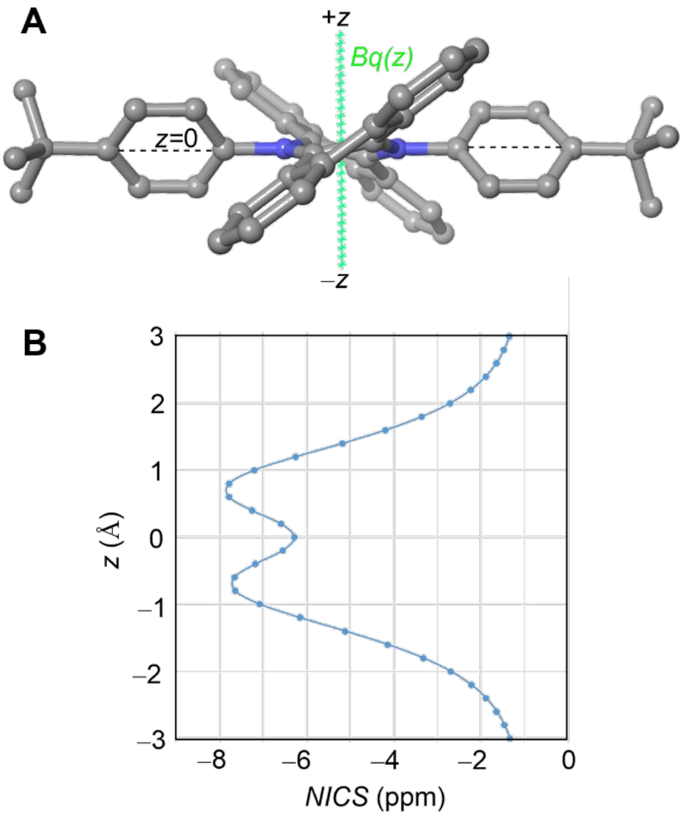

Figure S37. a) Orientation of the *Bq* points (in green) used for the calculation of different NICS with respect to the fully optimized structure of dication **4**; b) Plot of the calculated NICS values with respect to the z-axis, perpendicular to the average molecular plane of **4** and intersecting the ring point of the pyrazinium dication unit.

The aromatic character of **4** was also confirmed by magnetic criteria.[21] Thus, the evolution of the Nucleus Independent Chemical Shift (NICS)[22,23] was calculated along the *Bq* points within the axis perpendicular to the average molecular plane that intersects the ring point of electron density at the *N,N*-diarylphenazinium moiety. Analysis of the NICS vs z curve (Figure S37) shows a strong diamagnetic shielding effect at z= 0, with two maxima at ca. 0.75 Å above and below the average molecular plane. This latter value is close to the covalent radii of carbon and nitrogen and supports a π^2–aromatic character characterized by two diamagnetic ring currents[24,25] circulating above and below the molecular plane of dication **4**. In contrast, a similar analysis of the NICS profile for the thermodynamically stable boat conformer of *N,N*-diaryl dihydrophenazine **3**, a reduced precursor of dication **4**, shows an antiaromatic character of the 1,4-dihydrophenazine moiety (Figure S38). Thus, in the proximity of the molecular plane, the NICS values are positive. In this case, a diamagnetic shielding (NICS < 0) for $z > +2$ Å is observed. However, this effect is induced by the two eclipsed *p-tert*-butylphenyl groups and not by the 1,4-dihydrophenazine moiety.

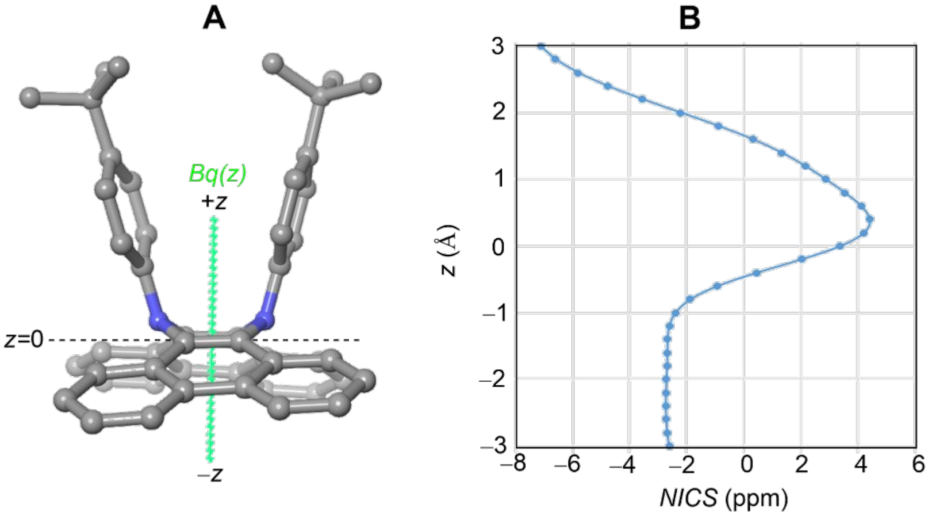

Figure S38. a) Orientation of the *Bq* points (in green) used for the calculation of different NICS with respect to the fully optimized structure of *N,N*-diaryl dihydrophenazine **3**, a reduced precursor of dication **4**; b) Plot of the calculated NICS values with respect to the z-axis, perpendicular to the average molecular plane of **3** and intersecting the ring point of the dihydrophenazine moiety.

It is interesting to note that the aromaticity of dication **4** is compatible with a strong delocalization of the positive charge along the π–system associated with the KS-MOs shown in Figure S36. This distribution of π–density minimizes the Coulombic repulsion within the two formally positively charged nitrogen atoms of the phenazine-1,4-diium ring. Thus, the electrostatic potential surface and the NBO charges of **4** indicate that the central cationic system accommodates a total charge of only +0.5e, whereas the remaining charge of +1.5e is extended along the entire polycyclic molecule (Figure S39). This result is in line with that reported for 2,3,6,7-tetramethoxythianthrene (TMT)[26] and hexaarylbutadiene[27] dications, for which different delocalization patterns for neutral and dicationic states induce strong geometrical changes. However, in the case of TMT^{2+} dication, delocalization, and not aromaticity, was responsible for the stability of the cationic species. In our case, both charge delocalization and aromaticity are compatible to each other. This moderate Coulombic repulsion agrees with the measured small separation (ΔE=0.23 V) between the two oxidation potentials observed for the oxidation of **3**.

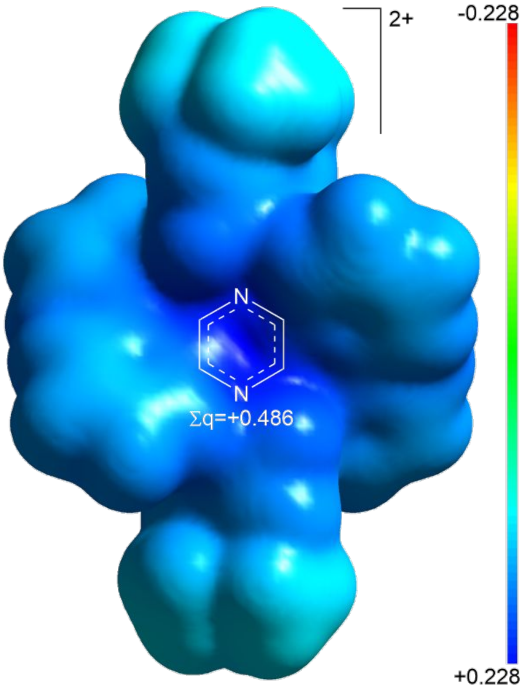

Figure S39. Electrostatic potential and collective NBO charge of the phenazine-1,4-diium moiety of dication **4**. The colors are represented according to the electrostatic potential, given in hartrees.

We have also estimated the aromatic character of dication **4** by calculating the internal and free energies associated with the isodesmic reactions shown in Scheme S1 and in Table S1. The first equation uses as base the conjugate dication **7a**. This selection permits to estimate the resonance energy as -91.6 kcal/mol, a quite high value. Taking dication **9a** as reference leads to an exothermic value of ca. -74 kcal/mol. Since **9a** is also aromatic, a significant part of the resonance energy reported in entry 1 of Table S1, stems from the thermodynamic instability of boat compound **3**. Including two phenyl groups in the boat conformation of reference compound **9b**, significantly diminishes the exothermic character of the corresponding isodesmic reaction (Table 1, entry 4). Therefore, the reaction energy of **3**+**9b**→**4**+**10b** process reflects the strain generated by the phenanthrene units of **3**. Taking the N-aryl effect into account, the resonance aromatic energy of **4** is estimated as 32.4 (internal energy) or 33.3 (Gibbs energy) kcal/mol, a value lower than the adiabatic resonance energy calculated for benzene (61.4 kcal/mol with the 6-31G* basis set).[28]

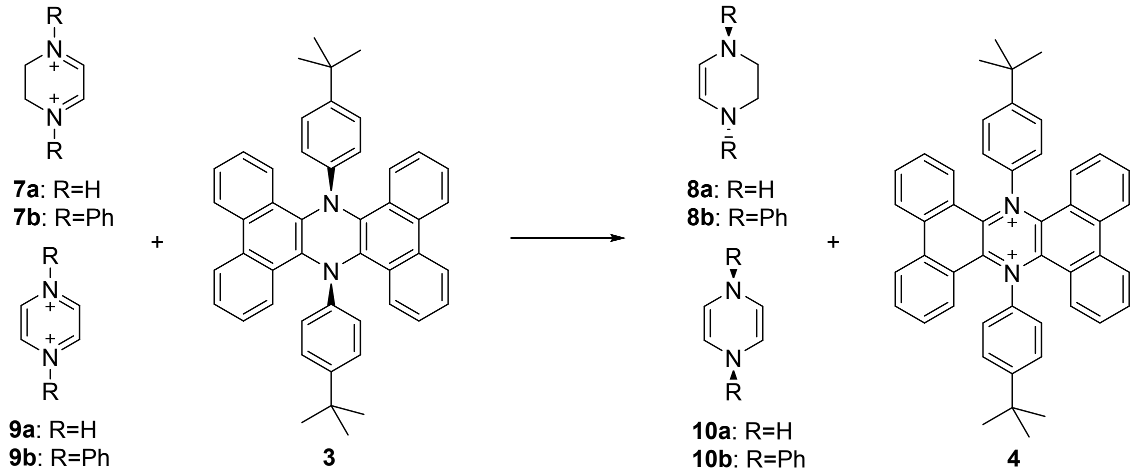

Scheme S1. Isodesmic reactions to assess the aromaticity of dication **4**.

Table S2. Internal and Gibbs (298K) energies, in kcal/mol, of the isodesmic reactions gathered in Scheme S1.[a]

Entry	Reaction	ΔE_{rxn}	ΔG_{rxn}
1	**3 + 7a → 4 + 8a**	-91.6	-91.6
2	**3 + 7b → 4 + 8b**	-32.4	-33.3
3	**3 + 9a → 4 + 10a**	-74.4	-74.7
4	**3 + 9b → 4 + 10b**	-21.8	-23.1

[a] Values computed at the B3LYP-D3BJ/6-31G* level of theory.

Finally, we have calculated the radical cation intermediates associated with the oxidation of neutral phenazine **3** to yield the corresponding dication **4**. This process takes place via two consecutive monoelectronic oxidations, in agreement with the electrochemical studies. We have found two radical cations associated with boat and half-chair conformations (Figure S40). This structural situation corresponds to the following elementary steps:

3 (boat) → $\mathbf{3}^{\cdot+}$ (boat) + e, (1)

$\mathbf{3}^{\cdot+}$ (boat) → $\mathbf{4}^{\cdot+}$ (half-chair), and (2)

$\mathbf{4}^{\cdot+}$ (half-chair) → **4** (half-chair) + e (3)

In addition, an alternative sequence of events can be envisaged, in which the second oxidation occurs *before* the conformational change:

3 (boat) → $\mathbf{3}^{\cdot+}$ (boat) + e^-, (1)

$\mathbf{3}^{\cdot+}$ (boat) → $\mathbf{3}^{++}$ (boat) + e^-, and (4)

$\mathbf{3}^{++}$ (boat) → **4** (half-chair) + e (5)

In order to analyze these two possible mechanisms, calculations were conducted at the B3LYP-D3BJ-SCRF (PCM, solvent=$CHCl_3$)/6-31G*//B3LYP-D3BJ/6-31G* level of theory. $CHCl_3$ was selected as solvent to be consistent with the cyclic voltammetry experiments (vide supra). The oxidation elementary reactions associated with eq. (1), (3) and (4) were modelled according to the reverse Born-Haber cycles shown in Figure S40.

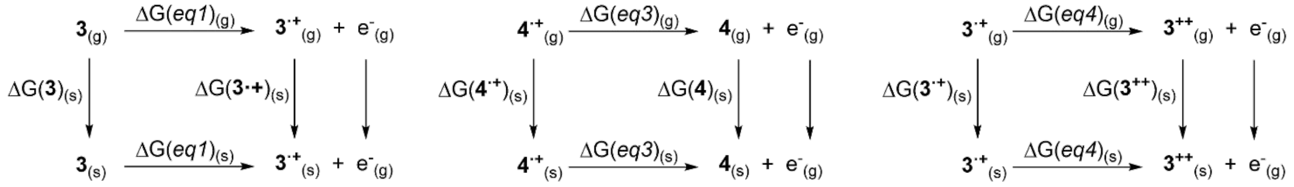

Figure S40. Reverse Born-Haber cycles associated with oxidation processes corresponding to eqs. (1), (3) and (4). Descriptors (g) and (s) stand for gas state and $CHCl_3$ solution, respectively.

In addition, for each oxidative step, the standard one-electron redox potentials E^0_{calc} were computed according to the Nerst equation:

$$E^0_{calc} = \frac{\Delta G(eq.X)_{(s)}}{F} \qquad (6),$$

in which F is the Faraday constant 23.06 kcal mol^{-1} V^{-1}.

Table S3. Free energies in solution ($\Delta G(eq.X)_{(s)}$, in kcal/mol at 298.15 K), one-electron redox potentials (E^0_{calc}, in V) and estimated one-electron redox potentials with respect to the experimental value of the ferrocene pair[29] (E^0_{calc} vs. $E^0_{\exp}(Fc/Fc^{\cdot+})$, in V) calculated for eqs. (1)-(5).[a]

Reaction		$\Delta G(eq.X)_{(s)}$	E^0_{calc}	E^0_{calc} vs. $E^0_{\exp}(Fc/Fc^{\cdot+})$
**3** (boat) → $\mathbf{3}^{\cdot+}$(boat) + e	(1)	120.9	5.24	0.09
$\mathbf{3}^{\cdot+}$ (boat) → $\mathbf{4}^{\cdot+}$ (half-chair)	(2)	2.6	--	--
$\mathbf{4}^{\cdot+}$ (half-chair) → **4** (half-chair) + e-	(3)	125.7	5.45	0.30
$\mathbf{3}^{\cdot+}$ (boat) → $\mathbf{3}^{++}$ (boat) + e^-	(4)	145.7	6.32	1.17
$\mathbf{3}^{++}$ (boat) → **4** (half-chair) + e	(5)	-17.4	--	--

[a]All values were calculated at the B3LYP-D3BJ-SCRF(PCM, solvent=$CHCl_3$)/6-31G*//B3LYP-D3BJ/6-31G* level of theory.

The values obtained for each equation (1)-(5) are collected in Table S3. According to our results, the first and second oxidative steps have associate redox potentials of 5.24 V (eq. (1)) and 5.45 V (eq. (3)). If the approximate experimental value of the ferrocene Fc/$Fc^{\cdot+}$ pair (ca. 5.15 V in CH_2Cl_2) is considered as a reference,[29] the corresponding redox potentials are 0.09 V and 0.30 V, which are in nice agreement with the experimentally observed values of 0.04 V and 0.27 V, respectively (see Main Text). The alternative process

associated with eq. (4) has a higher potential of 6.32 V (1.17 V vs. $Fc/Fc^{·+}$). These results indicate that, for both sets of equations, the second oxidation is more difficult that the first one. In the first mechanism, we were not able to locate the transition structure associated with the conformational change described by eq. (2), thus suggesting that the activation barrier, if existing, is very low in terms of free energy. Thus, despite the slightly endergonic character of this latter conformational change, most likely the (1)→(2)→(3) sequence is still feasible. In contrast, the (1)→(4)→(5) sequence, despite the noticeable exergonic character of step (5) (see also Figure S34), is less probable due to the difficult second oxidation associated with eq. (4) which can hamper the contribution of this mechanism to the formation of aromatic compound **4**.

Thus, according to our proposed mechanism, the first oxidation leads to a radical boat cation $\mathbf{3}^{·+}$. Given the conformational flexibility induced by the two *para-tert*-butylyphenyl groups, the loss of one electron results in a more planar boat structure, with a large RMS value with respect to **3**, mainly due to a ca. 3 Å increase in the distance between both *tert*-butyl groups (Figure S41). In this radical cation, the spin density is virtually coincident with the shape of the SOMO and shows a delocalization that goes beyond the nitrogen atoms and is distributed between the phenazine-9,18-diium and the two *para-tert*-butylphenyl groups. In contrast, radical cation $\mathbf{4}^{·+}$ exhibits the half-chair structure shown by aromatic dication **4**, with a very low RMS value between both charged species, thus indicating that the structural changes associated with the two-electron oxidation process correspond to the conformational conversion described in eq. (2). In addition, the spin density of $\mathbf{4}^{·+}$, very close to the corresponding SOMO, shows a less delocalized distribution of the unpaired electron, more confined to the phenazine-9,18-diium cycle (Figure S41). Consequently, $\mathbf{4}^{·+}$ is calculated to be ca. 2.6-2.9 kcal/mol less stable than $\mathbf{3}^{·+}$. Again, this relatively small difference in relative energies is also compatible with the low E^0_{calc} and $E^0_{\exp}$ vs. $Fc/Fc^{·+}$ values measured for the two oxidation waves associated with eq. (1) and (3).

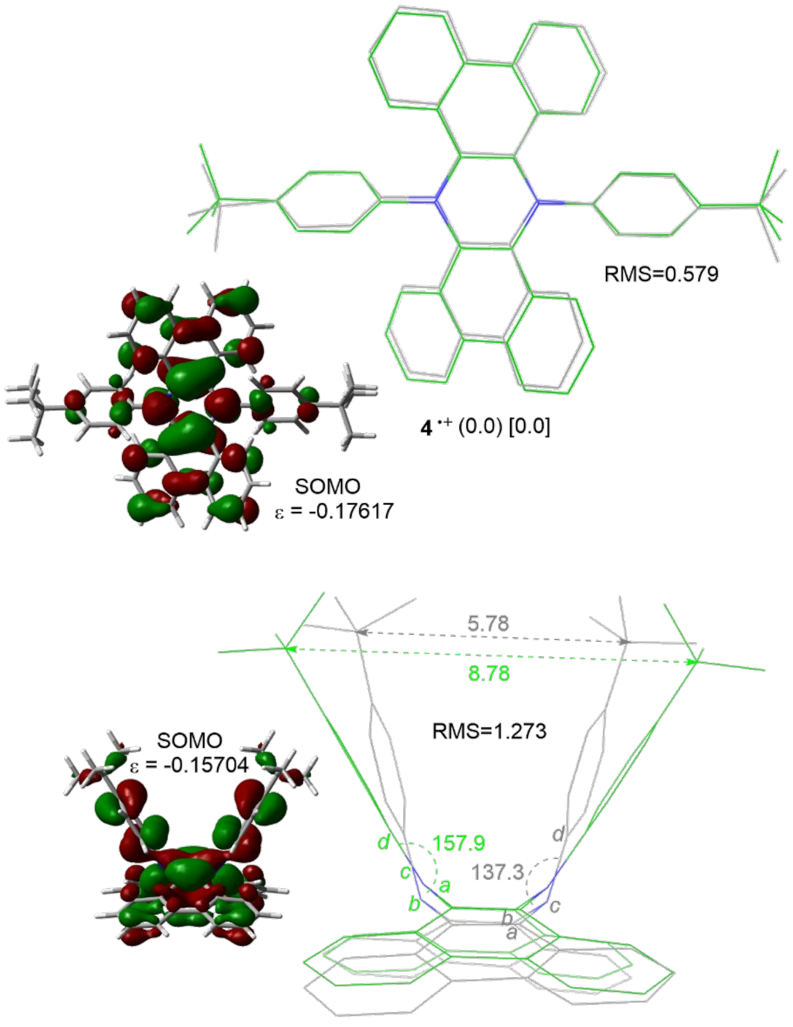

Figure S41. Calculated geometries (UB3LYP-D3BJ/6-31G* level of theory) of radical cations $3^{\cdot+}$ (boat) and $4^{\cdot+}$ (half-chair). Bond distances are given in Å. Dihedral angles (in absolute value) are given in deg. Orbital energies are given in hartrees. Numbers in parentheses and in square brackets are the relative total and Gibbs energies (at 298.15 K), in kcal/mol. The structures of the radical cations ($\langle S^2 \rangle \approx 0.75$ a. u.) are represented in green. The corresponding **4** and **3** structures are represented in grey. The RMS values are the Root Mean Squares between the corresponding structural pairs.

In summary, geometric, electronic, magnetic and energetic criteria show that dication **4** is π^2–aromatic, with two strongly delocalized π ring currents circulating at ca. 0.75 Å above and below the average molecular plane. This aromatic character is compatible with a minimization of the Coulombic repulsion between the nitrogen atoms and is somewhat diminished by a non-perfect planarity that in turn generates a non-optimal overlap of p-AO's along the *N,N*-diarylphenazinium moiety, as well as higher bond distances and lower (but significantly equalized) bond orders. The two-electron **3**→**4** oxidation takes place via radical cations $3^{\cdot+}$ and $4^{\cdot+}$, in which there is a structural transition between the boat conformation associated with neutral species **3** and the half-chair structure of aromatic dication **4**.

Table S4. Total energies,[a,b] zero-point vibrational energies[a,b] (ZPVE) and thermal corrections to Gibbs free energies (TCGFE)[a-c] of the stationary points discussed in this work.

Stationary point	Total energy		ZPVE	TCGFE
	Gas phase	$CHCl_3$[d]		
**4** (half-chair)	-1963.693316	-1963.834021	0.772734	0.699458
**4** (boat)	-1963.662812	-1963.803126	0.771259	0.696319
**4**($^{\cdot+}$) (half-chair)	-1963.994299	-1964.032117	0.771531	0.697272
**3**	-1964.221450	-1964.228402	0.770204	0.696756
**3**($^{\cdot+}$) (boat)	-1963.997019	-1964.034323	0.770452	0.695327
**7a**	-266.051081		0.126911	0.098610
**7b**	-728.302973		0.288797	0.247145
**8a**	-266.725173		0.124317	0.095937
**8b**	-728.882356		0.285947	0.242560
**9a**	-264.848032		0.104053	0.076542
**9b**	-727.092815		0.264704	0.223719
**10a**	-265.492955		0.099785	0.071512
**10b**	-727.655043		0.261511	0.218233

[a] Values computed at the B3LYP-D3BJ/6-31G* level of theory. All the stationary points exhibit positive definite Hessians (NIMAG=0).[b] In atomic units (hartree/particle).[c] Computed at 298K. [d]Total energies computed at the B3LYP-D3BJ-SCRF(PCM, solvent=$CHCl_3$)/6-31G*//B3LYP-D3BJ/6-31G* level of theory.

Cartesian coordinates

4 (half chair)

Center Number	Atomic Number	Atomic Type	Coordinates (Angstroms) X	Y	Z
1	7	0	1.360509	0.010351	0.003241
2	6	0	7.143891	-0.021262	0.001843
3	6	0	7.759890	0.903955	1.063587
4	1	0	7.471207	0.612581	2.079556
5	1	0	8.850379	0.845742	1.005990
6	1	0	7.480456	1.951668	0.906632
7	6	0	7.633457	0.433259	-1.392606
8	1	0	7.268923	-0.220073	-2.191658
9	1	0	7.306785	1.455780	-1.610643
10	1	0	8.727258	0.413819	-1.423208
11	6	0	7.617852	-1.467950	0.272435
12	1	0	7.278854	-1.816218	1.254084
13	1	0	7.253280	-2.169998	-0.484213
14	1	0	8.711534	-1.505955	0.259040
15	6	0	2.812897	0.011273	0.008263
16	6	0	3.490039	0.815309	0.919265
17	1	0	2.942947	1.434578	1.622598
18	6	0	4.882783	0.802066	0.922322
19	1	0	5.400358	1.421896	1.643224
20	6	0	5.614186	0.009150	0.023307
21	6	0	4.885991	-0.783357	-0.886360
22	1	0	5.414482	-1.403424	-1.601572
23	6	0	3.498132	-0.796085	-0.899532
24	1	0	2.955891	-1.414369	-1.607395
25	7	0	-1.360541	0.010321	-0.003177
26	6	0	-7.143933	-0.021292	-0.001977
27	6	0	-7.617878	-1.468013	-0.272440
28	1	0	-7.253548	-2.169937	0.484443
29	1	0	-8.711567	-1.505974	-0.259346
30	1	0	-7.278620	-1.816470	-1.253934
31	6	0	-7.633540	0.433402	1.392402
32	1	0	-7.268768	-0.219637	2.191584
33	1	0	-7.307129	1.456064	1.610174
34	1	0	-8.727331	0.413673	1.423073
35	6	0	-7.759903	0.903804	-1.063832
36	1	0	-7.471355	0.612178	-2.079771
37	1	0	-8.850394	0.845800	-1.006094
38	1	0	-7.480310	1.951507	-0.907125
39	6	0	-2.812930	0.011217	-0.008249
40	6	0	-3.490035	0.815207	-0.919326
41	1	0	-2.942898	1.434430	-1.622665
42	6	0	-4.882775	0.801971	-0.922445
43	1	0	-5.400323	1.421739	-1.643418
44	6	0	-5.614218	0.009112	-0.023412
45	6	0	-4.886064	-0.783368	0.886309
46	1	0	-5.414579	-1.403422	1.601519
47	6	0	-3.498202	-0.796105	0.899544
48	1	0	-2.956005	-1.414367	1.607460
49	6	0	0.691511	-1.180653	0.175955
50	6	0	1.277023	-2.396586	0.698122
51	6	0	2.403926	-2.412912	1.557318
52	1	0	2.913981	-1.496592	1.811937
53	6	0	2.840829	-3.593887	2.126542
54	1	0	3.689950	-3.580711	2.801366

55	6	0	2.177061	-4.796357	1.849179
56	1	0	2.518503	-5.723870	2.297035
57	6	0	1.063984	-4.800379	1.021554
58	1	0	0.537969	-5.732300	0.856956
59	6	0	0.586814	-3.617276	0.435212
60	6	0	-0.586767	-3.617305	-0.435041
61	6	0	-1.063886	-4.800430	-1.021382
62	1	0	-0.537879	-5.732345	-0.856727
63	6	0	-2.176910	-4.796443	-1.849078
64	1	0	-2.518292	-5.723969	-2.296951
65	6	0	-2.840668	-3.593990	-2.126530
66	1	0	-3.689748	-3.580849	-2.801407
67	6	0	-2.403824	-2.412995	-1.557304
68	1	0	-2.913872	-1.496686	-1.811985
69	6	0	-1.276981	-2.396635	-0.698026
70	6	0	-0.691512	-1.180672	-0.175863
71	6	0	-0.692131	1.200893	0.173155
72	6	0	0.692071	1.200898	-0.173098
73	6	0	1.278812	2.416591	-0.694282
74	6	0	2.407221	2.431776	-1.551338
75	1	0	2.918108	1.515089	-1.803560
76	6	0	2.844734	3.612173	-2.121254
77	1	0	3.694774	3.598450	-2.794925
78	6	0	2.180248	4.814915	-1.846189
79	1	0	2.522289	5.741930	-2.294620
80	6	0	1.065827	4.819849	-1.020378
81	1	0	0.539396	5.751864	-0.857468
82	6	0	0.587705	3.637174	-0.433923
83	6	0	-0.587751	3.637181	0.433952
84	6	0	-1.278871	2.416601	0.694323
85	6	0	-2.407264	2.431820	1.551407
86	1	0	-2.918171	1.515151	1.803648
87	6	0	-2.844744	3.612227	2.121330
88	1	0	-3.694768	3.598521	2.795019
89	6	0	-2.180242	4.814955	1.846246
90	1	0	-2.522250	5.741977	2.294688
91	6	0	-1.065839	4.819867	1.020412
92	1	0	-0.539396	5.751873	0.857490

4 (boat)

Center Number	Atomic Number	Atomic Type	Coordinates (Angstroms) X	Y	Z
1	7	0	-1.337500	-0.058461	-1.134166
2	6	0	-0.658209	-1.236202	-1.384926
3	6	0	0.945821	-3.557184	-1.875959
4	6	0	-1.286054	-2.539812	-1.369390
5	6	0	0.747035	-1.139630	-1.542007
6	6	0	1.522424	-2.254978	-1.955834
7	6	0	-0.459180	-3.698380	-1.501013
8	6	0	-2.299456	-0.038592	-0.022431
9	6	0	-3.920628	-0.082219	2.293225
10	6	0	-3.587327	0.485619	-0.064826
11	6	0	-1.813526	-0.595923	1.171160
12	6	0	-2.613231	-0.605877	2.302058
13	6	0	-4.376457	0.461430	1.086211
14	1	0	-4.009125	0.886683	-0.972590
15	1	0	-0.807776	-1.000618	1.214059

16	1	0	-2.204974	-1.023299	3.216048
17	1	0	-5.377388	0.868479	1.019349
18	6	0	-0.747118	1.139030	-1.542344
19	6	0	0.459013	3.697815	-1.502090
20	6	0	0.658150	1.235660	-1.385361
21	6	0	-1.522534	2.254221	-1.956516
22	6	0	-0.945955	3.556464	-1.877100
23	6	0	1.285937	2.539316	-1.370173
24	7	0	1.337442	0.058012	-1.134275
25	6	0	2.299518	0.038532	-0.022606
26	6	0	3.920798	0.083037	2.292938
27	6	0	3.587372	-0.485712	-0.064880
28	6	0	1.813639	0.596323	1.170789
29	6	0	2.613409	0.606719	2.301636
30	6	0	4.376560	-0.461081	1.086118
31	1	0	4.009130	-0.887137	-0.972498
32	1	0	0.807897	1.001056	1.213566
33	1	0	2.205217	1.024508	3.215488
34	1	0	5.377477	-0.868186	1.019365
35	6	0	-2.686168	-2.704059	-1.251369
36	1	0	-3.332240	-1.839977	-1.202457
37	6	0	-1.062721	-4.963018	-1.386763
38	1	0	-0.457485	-5.858474	-1.446439
39	6	0	-3.251571	-3.962460	-1.180887
40	1	0	-4.327295	-4.065504	-1.087357
41	6	0	-2.431914	-5.099029	-1.215205
42	1	0	-2.867616	-6.089010	-1.129104
43	6	0	2.793851	-2.073920	-2.558282
44	1	0	3.132257	-1.066622	-2.772648
45	6	0	3.539750	-3.162268	-2.963928
46	1	0	4.500844	-3.020345	-3.446212
47	6	0	3.023359	-4.454310	-2.779297
48	1	0	3.605196	-5.317009	-3.087323
49	6	0	1.743587	-4.643776	-2.267606
50	1	0	1.355950	-5.653012	-2.217157
51	6	0	2.686036	2.703670	-1.252163
52	1	0	3.332157	1.839636	-1.203073
53	6	0	1.062466	4.962509	-1.388054
54	1	0	0.457161	5.857910	-1.447897
55	6	0	3.251368	3.962121	-1.181926
56	1	0	4.327085	4.065246	-1.088404
57	6	0	2.431649	5.098634	-1.216470
58	1	0	2.867287	6.088657	-1.130525
59	6	0	-2.793968	2.072926	-2.558879
60	1	0	-3.132368	1.065542	-2.772853
61	6	0	-1.743688	4.642900	-2.269219
62	1	0	-1.356017	5.652147	-2.219223
63	6	0	-3.023462	4.453231	-2.780847
64	1	0	-3.605282	5.315808	-3.089246
65	6	0	-3.539870	3.161121	-2.964939
66	1	0	-4.500976	3.019015	-3.447144
67	6	0	4.772626	0.129459	3.562058
68	6	0	-4.772384	-0.128095	3.562413
69	6	0	-4.051254	0.666871	4.675747
70	1	0	-3.068168	0.246261	4.911021
71	1	0	-4.649388	0.641971	5.592009
72	1	0	-3.915380	1.714670	4.387405
73	6	0	-4.937500	-1.601186	4.001272
74	1	0	-3.975629	-2.077649	4.216835
75	1	0	-5.443559	-2.188313	3.227497
76	1	0	-5.541800	-1.649090	4.912550
77	6	0	-6.167214	0.481349	3.349078

78	1	0	-6.738461	-0.059605	2.586599
79	1	0	-6.113477	1.537589	3.063146
80	1	0	-6.734565	0.424693	4.282298
81	6	0	4.051485	-0.664836	4.675887
82	1	0	3.915412	-1.712754	4.388078
83	1	0	3.068503	-0.243945	4.911084
84	1	0	4.649743	-0.639572	5.592057
85	6	0	4.937964	1.602754	4.000108
86	1	0	5.444078	2.189386	3.225995
87	1	0	5.542301	1.651079	4.911340
88	1	0	3.976162	2.079469	4.215431
89	6	0	6.167350	-0.480298	3.348957
90	1	0	6.738659	0.060210	2.586207
91	1	0	6.113433	-1.536661	3.063509
92	1	0	6.734753	-0.423293	4.282124

4(·+) (half-chair)

Center Number	Atomic Number	Atomic Type	Coordinates (Angstroms) X	Y	Z
1	7	0	1.379757	0.009751	0.003913
2	6	0	7.163448	-0.021177	-0.000826
3	6	0	7.781166	0.904872	1.059302
4	1	0	7.489704	0.614639	2.074760
5	1	0	8.872515	0.848117	1.002003
6	1	0	7.497282	1.951333	0.902315
7	6	0	7.656332	0.433052	-1.392703
8	1	0	7.286873	-0.218733	-2.190753
9	1	0	7.326569	1.454888	-1.610025
10	1	0	8.750885	0.413783	-1.427490
11	6	0	7.642222	-1.464449	0.272216
12	1	0	7.301039	-1.810525	1.254023
13	1	0	7.272664	-2.167513	-0.480985
14	1	0	8.736644	-1.505646	0.257891
15	6	0	2.819288	0.010386	0.009845
16	6	0	3.507168	0.804775	0.921664
17	1	0	2.959213	1.421800	1.626029
18	6	0	4.900948	0.799996	0.920640
19	1	0	5.416206	1.424620	1.639512
20	6	0	5.631532	0.009772	0.022740
21	6	0	4.903539	-0.780594	-0.884741
22	1	0	5.430414	-1.404504	-1.598693
23	6	0	3.514773	-0.786453	-0.899944
24	1	0	2.971170	-1.402550	-1.608431
25	7	0	-1.379751	0.009784	-0.003738
26	6	0	-7.163442	-0.021112	0.000445
27	6	0	-7.642198	-1.464397	-0.272555
28	1	0	-7.272719	-2.167418	0.480725
29	1	0	-8.736623	-1.505586	-0.258335
30	1	0	-7.300923	-1.810535	-1.254309
31	6	0	-7.656458	0.433206	1.392246
32	1	0	-7.287080	-0.218532	2.190372
33	1	0	-7.326710	1.455053	1.609537
34	1	0	-8.751014	0.413946	1.426929
35	6	0	-7.781052	0.904876	-1.059800
36	1	0	-7.489489	0.614583	-2.075212
37	1	0	-8.872407	0.848128	-1.002607
38	1	0	-7.497181	1.951346	-0.902846
39	6	0	-2.819281	0.010432	-0.009806
40	6	0	-3.507069	0.804757	-0.921749

41	1	0	-2.959043	1.421732	-1.626103
42	6	0	-4.900849	0.799986	-0.920860
43	1	0	-5.416034	1.424564	-1.639823
44	6	0	-5.631524	0.009827	-0.022976
45	6	0	-4.903622	-0.780490	0.884622
46	1	0	-5.430569	-1.404353	1.598560
47	6	0	-3.514859	-0.786356	0.899958
48	1	0	-2.971327	-1.402413	1.608535
49	6	0	0.682658	-1.193472	0.155935
50	6	0	1.284879	-2.419213	0.652764
51	6	0	2.427470	-2.436431	1.490735
52	1	0	2.926187	-1.516821	1.753570
53	6	0	2.899362	-3.617343	2.030778
54	1	0	3.765701	-3.595671	2.684149
55	6	0	2.250445	-4.828803	1.756352
56	1	0	2.617525	-5.756755	2.183081
57	6	0	1.115631	-4.832769	0.965980
58	1	0	0.590251	-5.766586	0.807719
59	6	0	0.603720	-3.646039	0.406082
60	6	0	-0.603822	-3.646019	-0.405855
61	6	0	-1.115787	-4.832727	-0.965747
62	1	0	-0.590443	-5.766566	-0.807495
63	6	0	-2.250615	-4.828714	-1.756100
64	1	0	-2.617739	-5.756651	-2.182824
65	6	0	-2.899498	-3.617230	-2.030505
66	1	0	-3.765852	-3.595525	-2.683855
67	6	0	-2.427553	-2.436338	-1.490465
68	1	0	-2.926246	-1.516704	-1.753270
69	6	0	-1.284939	-2.419171	-0.652526
70	6	0	-0.682675	-1.193454	-0.155708
71	6	0	-0.683367	1.212759	0.152261
72	6	0	0.683397	1.212737	-0.152090
73	6	0	1.287687	2.438217	-0.646752
74	6	0	2.433431	2.454465	-1.480274
75	1	0	2.933507	1.534582	-1.739985
76	6	0	2.906862	3.634888	-2.020081
77	1	0	3.775312	3.612695	-2.670641
78	6	0	2.256739	4.846557	-1.749117
79	1	0	2.625168	5.774082	-2.175614
80	6	0	1.119197	4.851322	-0.962629
81	1	0	0.593139	5.785223	-0.806979
82	6	0	0.605384	3.665006	-0.403652
83	6	0	-0.605285	3.665035	0.403756
84	6	0	-1.287623	2.438270	0.646893
85	6	0	-2.433357	2.454579	1.480428
86	1	0	-2.933457	1.534720	1.740177
87	6	0	-2.906748	3.635032	2.020204
88	1	0	-3.775193	3.612883	2.670774
89	6	0	-2.256594	4.846674	1.749198
90	1	0	-2.624991	5.774223	2.175672
91	6	0	-1.119058	4.851382	0.962702
92	1	0	-0.592971	5.785262	0.807025

3

Center Number	Atomic Number	Atomic Type	Coordinates (Angstroms) X	Y	Z
1	7	0	1.020435	-0.219626	-1.323572
2	6	0	1.530040	-1.280519	-0.493480
3	6	0	2.514393	-3.291165	1.250210

4	6	0	2.086804	-2.472963	-1.064724
5	6	0	1.538072	-1.065632	0.853911
6	6	0	2.033409	-2.052818	1.769469
7	6	0	2.559223	-3.497336	-0.190968
8	6	0	-0.366250	-0.298094	-1.678452
9	6	0	-3.124594	-0.469444	-2.366059
10	6	0	-1.159351	0.850028	-1.760555
11	6	0	-0.965400	-1.537874	-1.943791
12	6	0	-2.311377	-1.609232	-2.281030
13	6	0	-2.509489	0.756884	-2.097375
14	1	0	-0.736507	1.820393	-1.532233
15	1	0	-0.386626	-2.450241	-1.877732
16	1	0	-2.733390	-2.589921	-2.480532
17	1	0	-3.083577	1.675712	-2.132080
18	6	0	1.512473	1.052974	-0.884132
19	6	0	2.620612	3.470229	0.105359
20	6	0	1.582507	1.266425	0.461259
21	6	0	1.967633	2.035993	-1.825059
22	6	0	2.496708	3.265294	-1.331622
23	6	0	2.178824	2.452947	1.002966
24	7	0	1.092501	0.216882	1.316900
25	6	0	-0.275283	0.325660	1.727857
26	6	0	-3.006381	0.553414	2.499778
27	6	0	-1.058707	-0.816532	1.942283
28	6	0	-0.869275	1.579071	1.911226
29	6	0	-2.207121	1.680732	2.288138
30	6	0	-2.391032	-0.693472	2.319043
31	1	0	-0.638843	-1.802395	1.786374
32	1	0	-0.299059	2.484036	1.745185
33	1	0	-2.622401	2.675006	2.407201
34	1	0	-2.965751	-1.604800	2.457283
35	6	0	2.173463	-2.641784	-2.464581
36	1	0	1.826371	-1.835085	-3.100471
37	6	0	3.078732	-4.672476	-0.779510
38	1	0	3.446994	-5.473379	-0.148794
39	6	0	2.691095	-3.801950	-3.003636
40	1	0	2.755127	-3.921117	-4.081257
41	6	0	3.140918	-4.827057	-2.151924
42	1	0	3.547321	-5.742478	-2.572146
43	6	0	2.026247	-1.822740	3.162683
44	1	0	1.661431	-0.868713	3.525656
45	6	0	2.465379	-2.795307	4.037697
46	1	0	2.453463	-2.611269	5.108008
47	6	0	2.928573	-4.026238	3.538613
48	1	0	3.271814	-4.795122	4.224765
49	6	0	2.953888	-4.263486	2.176932
50	1	0	3.320121	-5.219980	1.822106
51	6	0	2.324791	2.625933	2.397111
52	1	0	1.991642	1.827164	3.050240
53	6	0	3.181950	4.639558	0.666205
54	1	0	3.531009	5.434490	0.017252
55	6	0	2.879690	3.781335	2.908647
56	1	0	2.989392	3.904646	3.982118
57	6	0	3.307269	4.796725	2.034116
58	1	0	3.743974	5.707811	2.432836
59	6	0	1.875289	1.810660	-3.215805
60	1	0	1.473693	0.864343	-3.559445
61	6	0	2.899607	4.232428	-2.280090
62	1	0	3.301812	5.181550	-1.944948
63	6	0	2.792368	3.999566	-3.638562
64	1	0	3.109119	4.764419	-4.341761
65	6	0	2.279457	2.778573	-4.112644

66	1	0	2.202035	2.598376	-5.180879
67	6	0	-4.488142	0.634624	2.884020
68	6	0	-4.599594	-0.601523	-2.763851
69	6	0	-5.301852	-1.635993	-1.858560
70	1	0	-4.855644	-2.630814	-1.953448
71	1	0	-6.360949	-1.721231	-2.129898
72	1	0	-5.239341	-1.339271	-0.807310
73	6	0	-4.680988	-1.073734	-4.232082
74	1	0	-4.180639	-2.038397	-4.367745
75	1	0	-4.199582	-0.350491	-4.899649
76	1	0	-5.726472	-1.187366	-4.544451
77	6	0	-5.354550	0.732094	-2.638625
78	1	0	-4.946457	1.495755	-3.309585
79	1	0	-5.317609	1.120985	-1.614752
80	1	0	-6.407518	0.587786	-2.904398
81	6	0	-5.333626	-0.018572	1.769847
82	1	0	-5.066602	-1.070614	1.627391
83	1	0	-5.175588	0.496638	0.816115
84	1	0	-6.401761	0.028121	2.014855
85	6	0	-4.964912	2.085555	3.059784
86	1	0	-4.406413	2.602181	3.848224
87	1	0	-6.024010	2.095499	3.339997
88	1	0	-4.861370	2.660640	2.133035
89	6	0	-4.722575	-0.115002	4.212858
90	1	0	-4.135468	0.335385	5.020871
91	1	0	-4.435961	-1.168839	4.138798
92	1	0	-5.781810	-0.074773	4.494556

3(·+) (boat)

Center Number	Atomic Number	Atomic Type	Coordinates (Angstroms) X	Y	Z
1	7	0	1.314327	0.817590	0.033556
2	6	0	0.691189	1.234549	1.236799
3	6	0	-0.729021	2.053941	3.555847
4	6	0	1.439721	1.693677	2.373932
5	6	0	-0.691403	1.234493	1.236764
6	6	0	-1.440030	1.693491	2.373890
7	6	0	0.728631	2.054053	3.555862
8	6	0	2.095322	-0.368075	-0.036309
9	6	0	3.582097	-2.791151	-0.180267
10	6	0	2.463697	-0.915943	-1.275996
11	6	0	2.449949	-1.066377	1.133668
12	6	0	3.176834	-2.242303	1.050372
13	6	0	3.193406	-2.097316	-1.334740
14	1	0	2.171775	-0.436213	-2.199510
15	1	0	2.146107	-0.704493	2.105749
16	1	0	3.426845	-2.751766	1.974965
17	1	0	3.452640	-2.482201	-2.313280
18	6	0	0.690871	1.374394	-1.112704
19	6	0	-0.728715	2.472342	-3.313588
20	6	0	-0.690951	1.374398	-1.112732
21	6	0	1.439713	1.970461	-2.184370
22	6	0	0.728725	2.472317	-3.313573
23	6	0	-1.439743	1.970506	-2.184401
24	7	0	-1.314458	0.817542	0.033481
25	6	0	-2.095350	-0.368188	-0.036440
26	6	0	-3.581914	-2.791387	-0.180426
27	6	0	-2.449645	-1.066702	1.133508
28	6	0	-2.463966	-0.915891	-1.276127

29	6	0	-3.193568	-2.097332	-1.334880
30	6	0	-3.176430	-2.242687	1.050205
31	1	0	-2.145610	-0.704927	2.105571
32	1	0	-2.172329	-0.435993	-2.199640
33	1	0	-3.453019	-2.482079	-2.313419
34	1	0	-3.426159	-2.752330	1.974776
35	6	0	2.846651	1.825690	2.327654
36	1	0	3.370508	1.586666	1.409794
37	6	0	1.484643	2.471223	4.673627
38	1	0	0.983141	2.743356	5.593860
39	6	0	3.550642	2.250869	3.434495
40	1	0	4.630292	2.349981	3.387094
41	6	0	2.863117	2.559899	4.621409
42	1	0	3.413990	2.886498	5.497697
43	6	0	-2.846979	1.825279	2.327589
44	1	0	-3.370784	1.586191	1.409716
45	6	0	-3.551058	2.250310	3.434431
46	1	0	-4.630724	2.349241	3.387015
47	6	0	-2.863607	2.559415	4.621367
48	1	0	-3.414551	2.885895	5.497654
49	6	0	-1.485120	2.470959	4.673610
50	1	0	-0.983680	2.743143	5.593861
51	6	0	-2.846409	2.098545	-2.121326
52	1	0	-3.370361	1.749619	-1.239443
53	6	0	-1.484719	3.023835	-4.371766
54	1	0	-0.983162	3.405537	-5.252171
55	6	0	-3.550196	2.657168	-3.167255
56	1	0	-4.629628	2.751763	-3.107147
57	6	0	-2.862874	3.107565	-4.308246
58	1	0	-3.413726	3.539426	-5.137744
59	6	0	2.846385	2.098429	-2.121287
60	1	0	3.370318	1.749469	-1.239407
61	6	0	1.484762	3.023791	-4.371737
62	1	0	0.983228	3.405539	-5.252136
63	6	0	2.862920	3.107458	-4.308205
64	1	0	3.413800	3.539300	-5.137692
65	6	0	3.550208	2.657016	-3.167213
66	1	0	4.629646	2.751548	-3.107098
67	6	0	-4.389238	-4.089564	-0.214538
68	6	0	4.389625	-4.089201	-0.214356
69	6	0	3.566003	-5.214857	0.451758
70	1	0	3.328069	-4.984969	1.495151
71	1	0	4.134044	-6.151346	0.438057
72	1	0	2.623857	-5.378912	-0.082322
73	6	0	5.707166	-3.883844	0.567215
74	1	0	5.524765	-3.615997	1.612761
75	1	0	6.311318	-3.090164	0.114724
76	1	0	6.295174	-4.807948	0.557458
77	6	0	4.735954	-4.521862	-1.648142
78	1	0	5.339966	-3.768843	-2.166170
79	1	0	3.837222	-4.715888	-2.243920
80	1	0	5.317825	-5.448270	-1.619811
81	6	0	-3.565187	-5.215177	0.451128
82	1	0	-3.326993	-4.985417	1.494491
83	1	0	-2.623160	-5.378924	-0.083259
84	1	0	-4.133026	-6.151790	0.437419
85	6	0	-4.735870	-4.522009	-1.648316
86	1	0	-5.340062	-3.768944	-2.166069
87	1	0	-5.317658	-5.448470	-1.620004
88	1	0	-3.837265	-4.715863	-2.244339
89	6	0	-5.706609	-3.884632	0.567421
90	1	0	-6.311037	-3.090980	0.115249

91	1	0	-5.524010	-3.616974	1.612979
92	1	0	-6.294435	-4.808851	0.557619

7a

Center Number	Atomic Number	Atomic Type	Coordinates (Angstroms) X	Y	Z
1	1	0	-1.295383	-1.979271	0.237979
2	6	0	-0.720724	-1.189537	-0.253906
3	1	0	-0.801101	-1.330290	-1.339792
4	6	0	0.720019	-1.189931	0.253973
5	1	0	1.294273	-1.980122	-0.237619
6	1	0	0.800134	-1.330380	1.339913
7	7	0	-1.373470	0.115137	0.055452
8	1	0	-2.395419	0.123373	0.177228
9	7	0	1.373562	0.114342	-0.055603
10	1	0	2.395460	0.121970	-0.177834
11	6	0	0.735874	1.226708	-0.114592
12	1	0	1.260763	2.160737	-0.315241
13	6	0	-0.735140	1.227126	0.114715
14	1	0	-1.259552	2.161437	0.315281

7b

Center Number	Atomic Number	Atomic Type	Coordinates (Angstroms) X	Y	Z
1	6	0	-0.709961	1.158765	0.007236
2	7	0	-1.429329	0.052825	-0.088561
3	6	0	-0.664546	-1.198282	-0.367133
4	6	0	0.664545	-1.198295	0.367489
5	7	0	1.429350	0.052788	0.088907
6	6	0	0.710006	1.158755	-0.006735
7	1	0	-1.248689	-2.048849	-0.020155
8	1	0	-0.533258	-1.269455	-1.452094
9	1	0	1.248658	-2.048896	0.020551
10	1	0	0.533214	-1.269441	1.452448
11	6	0	-2.836691	0.030323	-0.022809
12	6	0	2.836697	0.030285	0.022874
13	1	0	1.226008	2.108832	-0.056352
14	1	0	-1.225937	2.108849	0.056922
15	6	0	3.520245	1.039137	-0.693984
16	6	0	4.903992	1.029620	-0.720008
17	6	0	5.615994	0.030511	-0.036192
18	6	0	4.934194	-0.973923	0.666311
19	6	0	3.548542	-0.991376	0.690659
20	1	0	2.977608	1.775023	-1.277781
21	1	0	5.435864	1.782866	-1.291091
22	1	0	6.700895	0.027394	-0.064207
23	1	0	5.489102	-1.739206	1.197970
24	1	0	3.037011	-1.754018	1.266153
25	6	0	-3.520438	1.039161	0.693890
26	6	0	-4.904188	1.029591	0.719627
27	6	0	-5.616013	0.030434	0.035692
28	6	0	-4.934022	-0.974002	-0.666620
29	6	0	-3.548364	-0.991398	-0.690680
30	1	0	-2.977976	1.775082	1.277801
31	1	0	-5.436205	1.782831	1.290582
32	1	0	-6.700920	0.027275	0.063484

33	1	0	-5.488779	-1.739343	-1.198353
34	1	0	-3.036692	-1.754074	-1.266004

8a

Center Number	Atomic Number	Atomic Type	Coordinates (Angstroms) X	Y	Z
1	6	0	0.666724	1.239328	0.074870
2	1	0	1.227212	2.164645	0.139183
3	6	0	-0.667228	1.239083	-0.074820
4	1	0	-1.228113	2.164184	-0.138726
5	7	0	-1.416434	0.045329	-0.204971
6	1	0	-2.343782	0.126535	0.198454
7	6	0	-0.711554	-1.140100	0.282894
8	1	0	-1.243169	-2.036749	-0.054286
9	1	0	-0.647505	-1.169315	1.382494
10	6	0	0.711992	-1.139911	-0.282752
11	1	0	0.647938	-1.169522	-1.382339
12	1	0	1.243923	-2.036279	0.054702
13	7	0	1.416471	0.045899	0.204744
14	1	0	2.343628	0.127507	-0.199053

8b

Center Number	Atomic Number	Atomic Type	Coordinates (Angstroms) X	Y	Z
1	7	0	-1.330275	-0.134430	-0.512060
2	6	0	-0.629670	1.054464	-0.221023
3	6	0	0.632091	1.054125	0.225234
4	7	0	1.330594	-0.137159	0.519226
5	6	0	0.757219	-1.314465	-0.120049
6	6	0	-0.753748	-1.317734	0.112702
7	1	0	-1.157480	1.980772	-0.413679
8	1	0	1.162048	1.979845	0.414364
9	6	0	2.743058	-0.019349	0.264931
10	1	0	1.200030	-2.213227	0.321181
11	1	0	0.939736	-1.334131	-1.205921
12	1	0	-0.936040	-1.352551	1.198295
13	1	0	-1.194559	-2.212329	-0.339322
14	6	0	-2.743519	-0.018195	-0.261399
15	6	0	3.590222	-1.108438	0.501995
16	6	0	4.963451	-0.993902	0.254765
17	6	0	5.489516	0.209724	-0.229529
18	6	0	4.642353	1.298813	-0.466592
19	6	0	3.269124	1.184276	-0.219362
20	1	0	3.181018	-2.044687	0.878707
21	1	0	5.622423	-1.841058	0.439167
22	1	0	6.557693	0.298816	-0.421839
23	1	0	5.051557	2.235061	-0.843304
24	1	0	2.610151	2.031431	-0.403764
25	6	0	-3.588479	-1.109067	-0.498129
26	6	0	-4.962466	-0.996062	-0.254431
27	6	0	-5.491493	0.207815	0.225997
28	6	0	-4.646533	1.298686	0.462728
29	6	0	-3.272546	1.185681	0.219030
30	1	0	-3.176972	-2.045511	-0.871834
31	1	0	-5.619725	-1.844604	-0.438574
32	1	0	-6.560260	0.295716	0.415560

33	1	0	-5.058040	2.235130	0.836432
34	1	0	-2.615287	2.034224	0.403172

9a

Center Number	Atomic Number	Atomic Type	Coordinates (Angstroms) X	Y	Z
1	1	0	-2.360897	-0.000002	0.000030
2	7	0	-1.331030	-0.000009	0.000008
3	6	0	-0.697864	1.189455	-0.000016
4	1	0	-1.291670	2.100074	0.000004
5	6	0	0.697859	1.189459	0.000004
6	1	0	1.291659	2.100082	0.000013
7	7	0	1.331030	-0.000003	0.000002
8	1	0	2.360898	0.000007	0.000012
9	6	0	0.697875	-1.189447	-0.000008
10	1	0	1.291658	-2.100083	0.000016
11	6	0	-0.697870	-1.189451	-0.000006
12	1	0	-1.291646	-2.100090	0.000007

9b

Center Number	Atomic Number	Atomic Type	Coordinates (Angstroms) X	Y	Z
1	6	0	0.686323	1.169111	-0.012185
2	7	0	1.392105	0.000035	0.000033
3	6	0	0.686388	-1.168981	0.011989
4	6	0	-0.686498	-1.168984	-0.011874
5	7	0	-1.392184	0.000012	-0.000104
6	6	0	-0.686514	1.169085	0.011618
7	6	0	2.823016	0.000052	0.000003
8	1	0	1.248516	-2.092025	0.055700
9	1	0	-1.248579	-2.092076	-0.055461
10	6	0	-2.823036	0.000002	-0.000036
11	1	0	-1.248689	2.092121	0.055100
12	1	0	1.248476	2.092115	-0.056137
13	6	0	-3.506447	0.993645	-0.723600
14	6	0	-4.894336	0.979926	-0.722180
15	6	0	-5.589157	-0.000052	0.000134
16	6	0	-4.894213	-0.980004	0.722358
17	6	0	-3.506322	-0.993661	0.723610
18	1	0	-2.972144	1.715625	-1.332862
19	1	0	-5.437577	1.720483	-1.299138
20	1	0	-6.674329	-0.000076	0.000201
21	1	0	-5.437358	-1.720590	1.299371
22	1	0	-2.971909	-1.715621	1.332803
23	6	0	3.506535	0.993291	0.724177
24	6	0	4.894401	0.979540	0.722811
25	6	0	5.589225	-0.000043	-0.000019
26	6	0	4.894305	-0.979614	-0.722723
27	6	0	3.506413	-0.993286	-0.724011
28	1	0	2.972168	1.714846	1.333856
29	1	0	5.437652	1.719741	1.300219
30	1	0	6.674398	-0.000045	-0.000039
31	1	0	5.437420	-1.719956	-1.300077
32	1	0	2.972011	-1.715020	-1.333468

10a

Center Number	Atomic Number	Atomic Type	Coordinates (Angstroms) X	Y	Z
1	7	0	1.441188	-0.000039	-0.120720
2	1	0	2.262657	-0.000055	0.479398
3	6	0	0.667692	-1.189747	0.022944
4	1	0	1.232346	-2.114776	0.045339
5	6	0	0.667758	1.189717	0.022939
6	1	0	1.232462	2.114713	0.045337
7	6	0	-0.667715	-1.189746	0.022889
8	1	0	-1.232367	-2.114780	0.045118
9	6	0	-0.667649	1.189778	0.022892
10	1	0	-1.232259	2.114837	0.045124
11	7	0	-1.441366	0.000037	-0.120806
12	1	0	-2.262113	0.000058	0.480391

10b

Center Number	Atomic Number	Atomic Type	Coordinates (Angstroms) X	Y	Z
1	6	0	0.669009	1.182435	0.873862
2	7	0	1.375628	-0.000088	0.561844
3	6	0	0.668967	-1.182617	0.873734
4	6	0	-0.669007	-1.182583	0.873632
5	7	0	-1.375571	-0.000024	0.561618
6	6	0	-0.668963	1.182473	0.873777
7	6	0	2.688640	-0.000033	0.080988
8	1	0	1.237454	-2.031050	1.233993
9	1	0	-1.237637	-2.030970	1.233783
10	6	0	-2.688601	0.000002	0.080863
11	1	0	-1.237534	2.030835	1.234077
12	1	0	1.237588	2.030778	1.234196
13	6	0	-3.365916	1.208726	-0.172143
14	6	0	-4.684794	1.199383	-0.616478
15	6	0	-5.362949	-0.000012	-0.835000
16	6	0	-4.684518	-1.199391	-0.617261
17	6	0	-3.365634	-1.208714	-0.172940
18	1	0	-2.859313	2.158009	-0.047572
19	1	0	-5.181643	2.147741	-0.802773
20	1	0	-6.390775	-0.000017	-1.183813
21	1	0	-5.181140	-2.147744	-0.804190
22	1	0	-2.858779	-2.157953	-0.049048
23	6	0	3.365576	1.208732	-0.172833
24	6	0	4.684422	1.199509	-0.617272
25	6	0	5.362899	0.000177	-0.835117
26	6	0	4.684839	-1.199267	-0.616577
27	6	0	3.366002	-1.208706	-0.172118
28	1	0	2.858663	2.157930	-0.048876
29	1	0	5.180975	2.147895	-0.804214
30	1	0	6.390687	0.000252	-1.184043
31	1	0	5.181737	-2.147584	-0.802951
32	1	0	2.859491	-2.158031	-0.047505

10 X-Ray Crystallography

Data collections were performed at the XRD2 beamline of the Elettra Synchrotron, Trieste (Italy).[30] The crystals were dipped in NHV oil (Jena Bioscience, Jena, Germany) and mounted on the goniometer head with kapton loops (MiTeGen, Ithaca, USA). Complete datasets were collected at 100 K for **3** or 298 K for **4**, **5** (nitrogen stream supplied through an Oxford Cryostream 700) through the rotating crystal method. Data were acquired using monochromatic wavelength of 0.620 Å on Pilatus hybrid-pixel area detectors (DECTRIS Ltd., Baden-Daettwil, Switzerland). The diffraction data were indexed, integrated and scaled using XDS.[31] Two different polymorphs of **3** have been found and characterized. A triclinic compact packing, called **3a**, and an alternative monoclinic crystal packing, called **3b**, has been found in the same crystallization batch. Two different datasets, collected from randomly oriented crystals, have been merged to obtain complete sets of data for **5** and **3a**, using CCP4-Aimless code.[32,33] Semi-empirical absorption corrections and scaling were performed on datasets, exploiting multiple measures of symmetry-related reflections, using SADABS program for **4**.[34] The structures were solved by the dual space algorithm implemented in the SHELXT code.[35] Fourier analysis and refinement were performed by the full-matrix least-squares methods based on F^2 implemented in SHELXL (Version 2018/3).[36] The Coot program was used for modeling.[37] Anisotropic thermal motion refinement have been used for all atoms with occupancies greater than 30%. Geometry and thermal motion parameters restrains (SIMU, DFIX and DANG) have been used on disordered fragments (i.e. solvent molecules and SbF_6^- counterions). Hydrogen atoms were included at calculated positions with isotropic $U_{factors} = 1.2 \cdot U_{eq}$ or $U_{factors} = 1.5 \cdot U_{eq}$ for methyl groups (U_{eq} being the equivalent isotropic thermal factor of the bonded non hydrogen atom). **3b** is characterized by wide packing voids filled with disordered solvent molecules: this electron density contribution couldn't be modeled and it has been removed with Platon SQUEEZE[38] routine (291 e^-/cell squeezed, corresponding to ~8 hexane and ~8 1,2-dichloroethane molecules/cell in 1233 $Å^3$ volume voids). Pictures were prepared using Ortep-3[39] and Pymol[40] software. Essential crystal and refinement data are reported below (Table S5 - contribution of disordered solvent removed by the SQUEEZE procedure is included in **3b** parameters).

Crystals of **4** and **5** show one crystallographic independent molecule in the asymmetric unit (ASU) with 2 and 1 hexafluoroantimonate counterions respectively, to balance the charges (Figure S42 – shortest $d_{SbF \cdots N}$ = 3.184(6) Å in the imidazolinium **5** cation and $d_{SbF \cdots N}$ = 2.889(8) Å in the phenazine **4** dication). The neutral compound **3** gives rise to two different crystal forms with completely different packing, bearing one molecule in the ASU for **3a** and two in **3b** monoclinic phase, with equivalent conformations (Figure S43). Crystal packing for **3**, **4** and **5** show extensive hydrophobic interactions with π•••π stacking of phenanthrene moieties and CH•••π contacts. **3a** and **3b** crystal forms differ for their solvent content and the space occupied by the solvent keep **3** molecules far, breaking stacking interactions (i.e. $d_{\pi \cdots \pi}$ = 3.579(1) Å with 0.34 Å slippage between ring centroids well overlapped in **3a**, compared to poorly overlapped rings in **3b** with $d_{\pi \cdots \pi}$ = 4.110(1) Å). CH•••π contacts are instead relevant in both **3a** and **3b** crystal packing (i.e. $d_{CH \cdots \pi}$ = 3.599(2) Å with 72° between CH and π-plane in **3a**, compared to $d_{CH \cdots \pi}$ = 3.540(2) Å with 57° in **3b**). The presence of

a carboxylate group in **5** gives rise to dimers in the solid state, bound through strong hydrogen bonds of facing –COOH groups ($d_{OH\cdots O}$ = 2.615(6) Å).

Table S5. Crystallographic data and refinement details for compounds **3** (**3a** and **3b**), **4** and **5**.

	**3a**	**3b**	**4**	**5**
CCDC Number	2129306	2129307	2129308	2129309
Chemical Formula	$C_{48}H_{42}N_2$	$C_{48}H_{42}N_2\cdot{}^3/_8(C_6H_{14})\cdot{}^3/_8(4H_4Cl_2)$	$[C_{48}H_{42}N_2](SbF_6)_2\cdot{}^3/_2(CHCl_3)$	$[C_{48}H_{43}N_2O_2](SbF_6)$
Formula weight	646.83 g/mol	716.25 g/mol	1297.39 g/mol	915.59 g/mol
Temperature	100(2) K	100(2) K	298(2) K	298(2) K
Wavelength	0.620 Å	0.620 Å	0.620 Å	0.620 Å
Crystal system	Triclinic	Monoclinic	Monoclinic	Triclinic
Space Group	*P* -1	*P* $2_1/n$	*P* $2_1/n$	*P* -1
Unit cell dimensions	*a* = 12.669(3) Å<br>*b* = 12.856(3) Å<br>*c* = 13.378(3) Å<br>*α* = 95.35(3)°<br>*β* = 113.56(3)°<br>*γ* = 111.60(3)°	*a* = 19.373(4) Å<br>*b* = 17.608(4) Å<br>*c* = 23.058(5) Å<br>*α* = 90°<br>*β* = 92.25(3)°<br>*γ* = 90°	*a* = 16.451(3) Å<br>*b* = 18.392(4) Å<br>*c* = 18.763(4) Å<br>*α* = 90°<br>*β* = 102.69(3)°<br>*γ* = 90°	*a* = 9.976(2) Å<br>*b* = 10.982(2) Å<br>*c* = 20.779(4) Å<br>*α* = 87.17(3)°<br>*β* = 85.47(3)°<br>*γ* = 87.39(3)°
Volume	1781.4(8) $Å^3$	7859(3) $Å^3$	5538(2) $Å^3$	2264.6(8) $Å^3$
Z	2	8	4	2
Density (calculated)	1.206 $g\cdot cm^{-3}$	1.211 $g\cdot cm^{-3}$	1.556 $g\cdot cm^{-3}$	1.343 $g\cdot cm^{-3}$
Absorption coefficient	0.053 mm^{-1}	0.086 mm^{-1}	0.864 mm^{-1}	0.463 mm^{-1}
F(000)	688	3052	2564	932
Theta range for data collection	1.5° to 28.0°	1.2° to 31.1°	1.3° to 30.0°	0.9° to 26.3°
Index ranges	-19 ≤ h ≤ 19,<br>-19 ≤ k ≤ 19,<br>-18 ≤ l ≤ 18	-29 ≤ h ≤ 29,<br>-29 ≤ k ≤ 29,<br>-38 ≤ l ≤ 37	-25 ≤ h ≤ 25,<br>-24 ≤ k ≤ 24,<br>-28 ≤ l ≤ 28	-14 ≤ h ≤ 14,<br>-15 ≤ k ≤ 15,<br>-29 ≤ l ≤ 29
Reflections collected	63464	188241	130020	54487
Independent reflections (data with $I>2\sigma(I)$)	12362 (5883)	34111 (16953)	21373 (12461)	13702 (7448)
Resolution	0.66 Å	0.60 Å	0.62 Å	0.70 Å
Data multiplicity (max resltn)	4.89 (2.63)	4.90 (2.80)	5.26 (3.80)	3.94 (3.52)
$I/\sigma(I)$ (max resltn)	4.24 (1.47)	9.25 (1.55)	7.74 (1.34)	5.52 (0.89)
R_{merge} (max resltn)	0.1241 (0.4138)	0.0697 (0.5111)	0.0677 (0.6625)	0.0498 (0.8141)
Data completeness (max resltn)	95.2% (89.0%)	89.7% (72.8%)	87.8% (75.1%)	99.0% (98.1%)
Refinement method	Full-matrix least-squares on F^2	Full-matrix least-squares on F^2	Full-matrix least-squares on F^2	Full-matrix least-squares on F^2
Data / restraints / parameters	12362 / 0 / 457	34111 / 0 / 913	21373 / 73 / 717	13702/ 6 / 553
Goodness-of-fit on F^2	0.958	1.016	1.044	1.035
Δ/σ_{max}	0.000	0.001	0.001	0.001
Final R indices [$I>2\sigma(I)$]	R_1 = 0.0684<br>wR_2 = 0.1479	R_1 = 0.0747<br>wR_2 = 0.2024	R_1 = 0.0773<br>wR_2 = 0.2517	R_1 = 0.0858<br>wR_2 = 0.2427
R indices (all data)	R_1 = 0.1607<br>wR_2 = 0.1862	R_1 = 0.1434<br>wR_2 = 0.2423	R_1 = 0.1101<br>wR_2 = 0.2913	R_1 = 0.1365<br>wR_2 = 0.2798
Largest diff. peak and hole	0.315 and -0.309 $eÅ^{-3}$	0.404 and -0.408 $eÅ^{-3}$	1.175 and -1.614 $eÅ^{-3}$	1.690 and -0.743 $eÅ^{-3}$
R.M.S. deviation from mean	0.062 $eÅ^{-3}$	0.057 $eÅ^{-3}$	0.136 $eÅ^{-3}$	0.078 $eÅ^{-3}$

$$R_1 = \Sigma \left|\left|Fo\right|-\left|Fc\right|\right| / \Sigma \left|Fo\right|,\ wR_2 = \{\Sigma [w(Fo^2 - F4\ )^2] / \Sigma [w(Fo^2\ )^2]\}^{½}$$

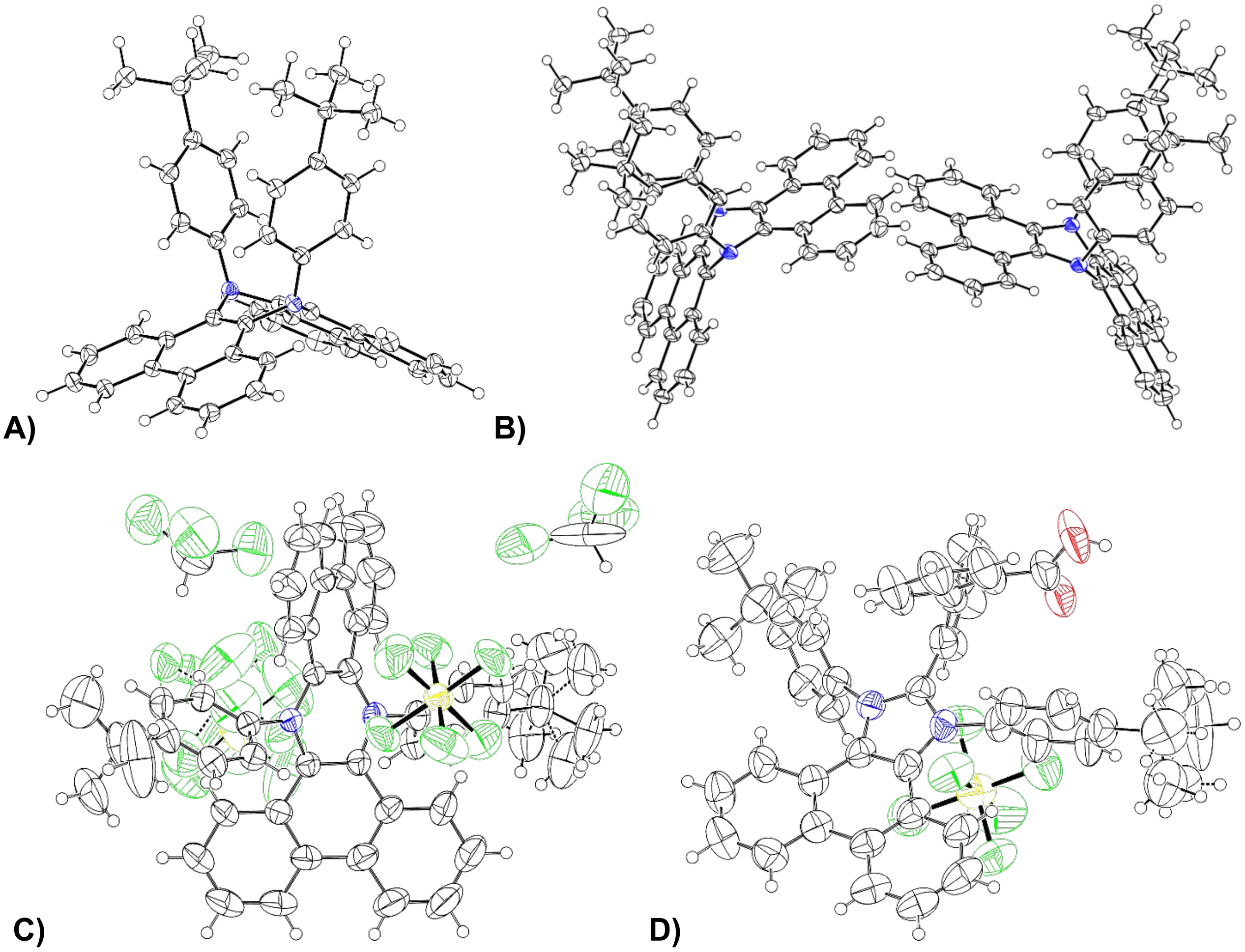

Figure S42. Ellipsoids representation of (A) **3a**, (B) **3b**, (C) **4**, and (D) **5** ASU contents (50% probability).

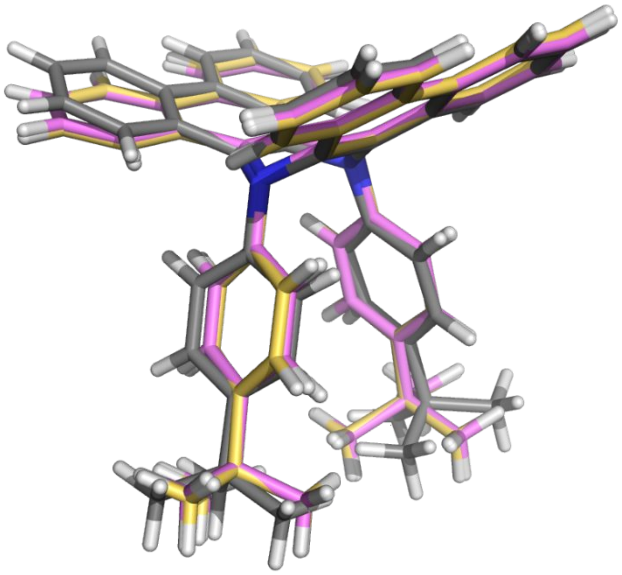

Figure S43. Stick representation for overlapped molecular models of **3a** (grey sticks) and **3b** (yellow and magenta sticks; R.M.S.D. ~0.93 Å).

11 References

(1) Lee, C.; Yang, W.; Parr, R. G. Development of the Colle-Salvetti correlation-energy formula into a functional electron density. *Phys. Rev. B* **1988**, *37* (2), 785–789.

(2) Vosko, S. H.; Wilk, L.; Nusair, M. Accurate spin-dependent electron liquid correlation energies for local spin density calculations: a critical analysis. *Can. J. Phys.* **1980**, *58* (8), 1200–1211.

(3) Stephen, P. J.; Devlin, F. J.; Chabalowski, C. F.; Frisch, M. J. Ab Initio Calculation of Vibrational Absorption. *J. Phys. Chem.* **1994**, *98* (45), 11623–11627.

(4) Becke, A. D. Density-functional thermochemistry. I. The effect of the exchange-only gradient correction. *J. Chem. Phys.* **1992**, *96* (3), 2155–2160.

(5) Frisch, M. J.; Trucks, G. W.; Schlegel, H. B.; Scuseria, G. E.; Robb, M. a.; Cheeseman, J. R.; Scalmani, G.; Barone, V.; Petersson, G. a.; Nakatsuji, H.; et al. Gaussian 16, Revision C.01, Gaussian, Inc., Wallin. 2016.

(6) Grimme, S.; Antony, J.; Ehrlich, S.; Krieg, H. A consistent and accurate ab initio parametrization of density functional dispersion correction (DFT-D) for the 94 elements H-Pu. *J. Chem. Phys.* **2010**, *132* (15).

(7) Grimme, S. Software News and Updates Gabedit — A Graphical User Interface for Computational Chemistry Softwares. *J. Comput. Chem.* **2006**, *27*, 174–182.

(8) Grimme, S.; Ehrlich, S.; Goerigk, L. Software News and Updates Gabedit — A Graphical User Interface for Computational Chemistry Softwares. *J. Comput. Chem.* **2011**, *32*, 1456–1465.

(9) Glendening, E. D.; Reed, A. E.; Carpenter, J. E.; Weinhold, F. scholar. *NBO Version 3.1. Gaussian Inc., Pittsburgh 2003.*

(10) Gauss, J. Calculation of NMR chemical shifts at second-order many-body perturbation theory using gauge-including atomic orbitals. *Chem. Phys. Lett.* **1992**, *191* (6), 614–620.

(11) Tomasi, J.; Mennucci, B.; Cammi, R. Quantum mechanical continuum solvation models. *Chem. Rev.* **2005**, *105* (8), 2999–3093.

(12) Miertuš, S.; Scrocco, E.; Tomasi, J. Electrostatic interaction of a solute with a continuum. A direct utilizaion of AB initio molecular potentials for the prevision of solvent effects. *Chem. Phys.* **1981**, *55* (1), 117–129.

(13) Wiberg, K. B. Application of the pople-santry-segal CNDO method to the cyclopropylcarbinyl and cyclobutyl cation and to bicyclobutane. *Tetrahedron* **1968**, *24* (3), 1083–1096.

(14) Reed, A. E.; Curtiss, L. A.; Weinhold, F. Intermolecular Interactions from a Natural Bond Orbital, Donor—Acceptor Viewpoint. *Chem. Rev.* **1988**, *88* (6), 899–926.

(15) Bird, C. W. A new aromaticity index and its application to five-membered ring heterocycles. *Tetrahedron* **1985**, *41* (7), 1409–1414.

(16) Bird, C. W. The application of a new aromaticity index to six-membered ring heterocycles. *Tetrahedron* **1986**, *42* (1), 89–92.

(17) Krygowski, T. M.; Cyrański, M. K. Structural aspects of aromaticity. *Chem. Rev.* **2001**, *101* (5), 1385–1419.

(18) Kruszewski, J.; Krygowski, T. M. Definition of aromaticity basing on the harmonic oscillator model. *Tetrahedron Lett.* **1972**, *13* (36), 3839–3842.

(19) Krygowski, T. M. Crystallographic Studies of Inter- and Intramolecular Interactions Reflected in Aromatic Character of π-Electron Systems. *J. Chem. Inf. Comput. Sci.* **1993**, *33* (1), 70–78.

(20) Andrzejak, M.; Kubisiak, P.; Zborowski, K. K. Avoiding pitfalls of a theoretical approach: The harmonic oscillator measure of aromaticity index from quantum chemistry calculations. *Struct. Chem.* **2013**, *24* (4), 1171–1184.

(21) Gershoni-Poranne, R.; Stanger, A. Magnetic criteria of aromaticity. *Chem. Soc. Rev.* **2015**, *44* (18), 6597–6615.

(22) Chen, Z.; Wannere, C. S.; Corminboeuf, C.; Puchta, R.; von Ragué Schleyer, P. Nucleus-independent chemical shifts (NICS) as an aromaticity criterion. *Chem. Rev.* **2005**, *105* (10), 3842–3888.

(23) Schleyer, P. V. R.; Maerker, C.; Dransfeld, A.; Jiao, H.; Van Eikema Hommes, N. J. R. Nucleus-independent chemical shifts: A simple and efficient aromaticity probe. *J. Am. Chem. Soc.* **1996**, *118* (26), 6317–6318.

(24) Morao, I.; Cossío, F. P. A simple ring current model for describing in-plane aromaticity in pericyclic reactions. *J. Org. Chem.* **1999**, *64* (6), 1868–1874.

(25) Cossío, F. P.; Morao, I.; Jiao, H.; Von Ragué Schleyer, P. In-plane aromaticity in 1,3-dipolar cycloadditions. Solvent effects, selectivity, and nucleus-independent chemical shifts. *J. Am. Chem. Soc.* **1999**, *121* (28), 6737–6746.

(26) Bock, H.; Rauschenbach, A.; Ruppert, K.; Havlas, Z. 2,3,6,7-Tetramethoxythianthrene Dication: An “Aromatic” π System “Gives Up the Ghost.” *Angew. Chemie Int. Ed.* **1991**, *30* (6), 714–716.

(27) Ishigaki, Y.; Harimoto, T.; Sugimoto, K.; Wu, L.; Zeng, W.; Ye, D.; Suzuki, T. Hexaarylbutadiene: A Versatile Scaffold with Tunable Redox Properties towards Organic Near-Infrared Electrochromic Material. *Chem. Asian J.* **2020**, *15* (7), 1147–1155.

(28) Mo, Y. The resonance energy of benzene: A revisit. *J. Phys. Chem. A* **2009**, *113* (17), 5163–5169.

(29) Connelly, N. G.; Geiger, W. E. Chemical redox agents for organometallic chemistry. *Chem. Rev.* **1996**, *96* (2), 877–910.

(30) Lausi, A.; Polentarutti, M.; Onesti, S.; Plaisier, J. R.; Busetto, E.; Bais, G.; Barba, L.; Cassetta, A.; Campi, G.; Lamba, D.; et al. Status of the crystallography beamlines at Elettra. *Eur. Phys. J. Plus* **2015**, *130* (3), 2–8.

(31) Kabsch, W. XDS. *Acta Crystallogr. Sect. D Biol. Crystallogr.* **2010**, *66* (2), 125–132.

(32) Winn, M. D.; Ballard, C. C.; Cowtan, K. D.; Dodson, E. J.; Emsley, P.; Evans, P. R.; Keegan, R. M.; Krissinel, E. B.; Leslie, A. G. W.; McCoy, A.; et al. Overview of the CCP4 suite and current developments. *Acta Crystallogr. Sect. D Biol. Crystallogr.* **2011**, *67* (4), 235–242.

(33) Evans, P. R.; Murshudov, G. N. How good are my data and what is the resolution? *Acta Crystallogr. Sect. D Biol. Crystallogr.* **2013**, *69* (7), 1204–1214.

(34) Sheldrick, G. M. SADABS Version 2014/15, University of Göttigen, Germany. **2014**.

(35) Sheldrick, G. M. SHELXT - Integrated space-group and crystal-structure determination. *Acta Crystallogr. Sect. A Found. Crystallogr.* **2015**, *71* (1), 3–8.

(36) Sheldrick, G. M. Crystal structure refinement with SHELXL. *Acta Crystallogr. Sect. C Struct. Chem.* **2015**, *71*, 3–8.

(37) Emsley, P.; Lohkamp, B.; Scott, W. G.; Cowtan, K. Features and development of Coot. *Acta Crystallogr. Sect. D Biol. Crystallogr.* **2010**, *66* (4), 486–501.

(38) Spek, A. L. PLATON SQUEEZE: A tool for the calculation of the disordered solvent contribution to the calculated structure factors. *Acta Crystallogr. Sect. C Struct. Chem.* **2015**, *71*, 9–18.

(39) Farrugia, L. J. WinGX and ORTEP for Windows: An update. *J. Appl. Crystallogr.* **2012**, *45* (4), 849–854.

(40) Schroedinger, L. the PyMOL molecular graphics system. Schrodinger, LLC. http://www.pymol.org. **2015**.
